# Kynurenic acid mediates epicardial fat-induced lymphatic metabolic dysfunction in atrial fibrillation

**DOI:** 10.1038/s41467-026-72974-9

**Published:** 2026-05-19

**Authors:** Masaki Takahashi, Ichitaro Abe, Naofumi Yoshida, Daisuke Katoh, Daishi Fujita, Shunsuke Goto, Hirochika Yamasaki, Toshiyuki Ko, Yue Jiang, Yu He, Shinji Ito, Taisuke Harada, Masayuki Takano, Hiroki Sato, Hidekazu Kondo, Akira Fukui, Yasushi Teshima, Hidenori Sako, Keisuke Ohta, Shinji Miyamoto, Toshimasa Yamauchi, Seitaro Nomura, Norihiko Takeda, Issei Komuro, Naohiko Takahashi

**Affiliations:** 1 Department of Cardiology and Clinical Examination, Oita University Faculty of Medicine, Oita, Japan; 2 Department of Cardiovascular Medicine, The University of Tokyo, Tokyo, Japan; 3 Department of Frontier Cardiovascular Science, The University of Tokyo Graduate School of Medicine, Tokyo, Japan; 4 Department of Advanced Medical Technologies, National Cerebral and Cardiovascular Center, Osaka, Japan; 5 Laboratory of Cardiovascular Mosaicism, National Cerebral and Cardiovascular Center, Osaka, Japan; 6 Division of Cardiovascular Medicine, Department of Internal Medicine, Kobe University Graduate School of Medicine, Kobe, Japan; 7 Department of Pathology and Matrix Biology, Mie University Graduate School of Medicine, Tsu, Mie Japan; 8 Institute for Integrated Cell-Material Sciences (iCeMS), Institute for Advanced Study, Kyoto University Kyoto, Japan; 9 Medical Research Support Center, Graduate School of Medicine, Kyoto University, Kyoto, Japan; 10 Department of Cardiovascular Surgery, Oka Hospital, Oita, Japan; 11 Division of Microscopic and Developmental Anatomy, Department of Anatomy, Kurume University School of Medicine, Kurume, Japan; 12 Advanced Imaging Research Center, Kurume University School of Medicine, Kurume, Japan; 13 Department of Cardiovascular Surgery, Oita University Faculty of Medicine, Oita, Japan; 14 Department of Diabetes and Metabolic Diseases, Graduate School of Medicine, The University of Tokyo, Tokyo, Japan; 15 International University of Health and Welfare, Tokyo, Japan

**Keywords:** Atrial fibrillation, Lipid signalling

## Abstract

Atrial fibrillation represents a prevalent cardiac arrhythmia whose pathogenic mechanisms remain incompletely understood. Here, we identify impaired atrial lymphangiogenesis as a critical determinant in atrial fibrillation pathogenesis. Analysis of human left atrial appendage specimens reveals decreased lymphatic vessel density in atrial fibrillation patients compared to those in sinus rhythm. Mechanistically, we demonstrate that epicardial adipose tissues from atrial fibrillation patients secrete kynurenic acid, which acts via GPR35 to disrupt lymphatic endothelial cell metabolism and mitochondrial homeostasis, ultimately promoting endothelial-to-mesenchymal transition. Using an organotypic culture system, we show that epicardial adipose tissue -derived factors directly impair lymphatic vessel formation. In vivo studies utilizing angiotensin II-induced and high-fat diet male mouse models confirm the critical role of lymphatic dysfunction in atrial fibrillation susceptibility. Therapeutic interventions promoting lymphangiogenesis, either through VEGFC administration or weight loss intervention by LY3437943 (the novel triple GIP, GLP-1, and glucagon receptor agonist), significantly attenuate atrial fibrillation inducibility. These findings establish lymphatic dysfunction as a novel pathogenic mechanism in atrial fibrillation and highlight lymphatic vessel formation as a promising therapeutic target.

## Abstract (web summary)

The role of cardiac lymphatics in atrial fibrillation, a common arrhythmia, remains unexplored. Here, the authors show that epicardial fat secretes kynurenic acid, which impairs atrial lymphatic endothelial function, and show that promoting lymphangiogenesis reduces atrial fibrillation susceptibility.

## Introduction

Atrial fibrillation (AFib) represents the most prevalent sustained cardiac arrhythmia, affecting millions worldwide and significantly contributing to cardiovascular morbidity and mortality^1^. In patients with AFib, the atria typically remodel and become fibrotic^2–4^. The distributed atrial fibrosis creates a substrate for abnormal electrical propagation by altering local cellular membrane kinetics, causing slow and disconnected signal conduction and giving rise to reentrant electrical waves^5,6^. Contemporary therapeutic approaches employ catheter-based ablation to attenuate the arrhythmogenic potential of fibrotic substrates through regional disruption of electrical conductivity. However, clinical outcomes remain suboptimal, with success rates ranging from 59% to 71% in persistent AFib cases^7,8^.

The lymphatic vascular system consists of network of thin-walled vessels encompassed by continuous endothelium, functioning as a unidirectional conduit for the retrograde transport of filtered arterial and tissue metabolites^9,10^. This vasculature maintains fluid homeostasis via the systematic removal and circulation of protein-rich lymph from the interstitial space^11^. In cardiac tissue, lymphatic drainage serves as a fundamental mechanism for maintaining physiological function and preventing maladaptive remodeling^12^. Emerging evidence has demonstrated the involvement of cardiac lymphatics in various cardiovascular pathologies, including myocardial infarction and heart failure, in both human and rodent models^13–18^. The identification of these contributions provides new opportunities for delineating the regulating processes of atrial lymphangiogenesis, *i.e*., how pathophysiological cues affect the processes of atrial lymphangiogenesis and maintenance in humans and rodents.

One of the current knowledge gaps in the field is our limited understanding of atrial lymphangiogenesis. This remains a significant area of research for the following reasons: *First*, an expansion of the atrial myocardial intercellular space—a process regulated by lymphatic fluid homeostasis—precedes atrial interstitial fibrosis^19^. Thus, delineating the mechanisms governing atrial lymphangiogenesis may facilitate the development of lymphatic-targeted therapeutic strategies for AFib management. *Second*, there is a long-standing debate as to what are the appropriate substrate targets for AFib ablation in patients with fibrosis^20^. Targeting atrial low-voltage areas and visualized atrial fibrosis with contrast-enhanced magnetic resonance imaging has not achieved superior outcomes^21–23^. The failure of these strategies to improve procedure success stems from the fact that there is currently no understanding as to what constitutes an appropriate target in the atrial fibrotic substrate. Hence, identifying the regulatory mechanisms for atrial lymphatics and understanding the various conditions that impair lymphangiogenesis may enable us to elucidate a suitable target for the treatment of AFib, other than atrial fibrosis. *Third*, the lymphatic system’s established role in lipid trafficking and inflammation positions it as a crucial mediator of adipose tissue function^24^. This relationship is particularly relevant to AFib pathogenesis, as epicardial adipose tissue (EAT) exerts pro-fibrotic effects through both its anatomical proximity to the atrial myocardium and paracrine signaling mechanisms^25,26^.

In this study, we demonstrate that EAT-derived kynurenic acid disrupts lymphatic endothelial cell function and promotes endothelial-to-mesenchymal transition, leading to reduced lymphatic vessel density. Furthermore, by using AFib mice models, we show dynamics of atrial lymphatics under conditions that predispose to AFib. The identification of these pathways and conditions advances our understanding of AFib pathophysiology and suggests potential new therapeutic targets.

## Results

### Impaired atrial lymphangiogenesis in human AFib

**Fig. 1:**
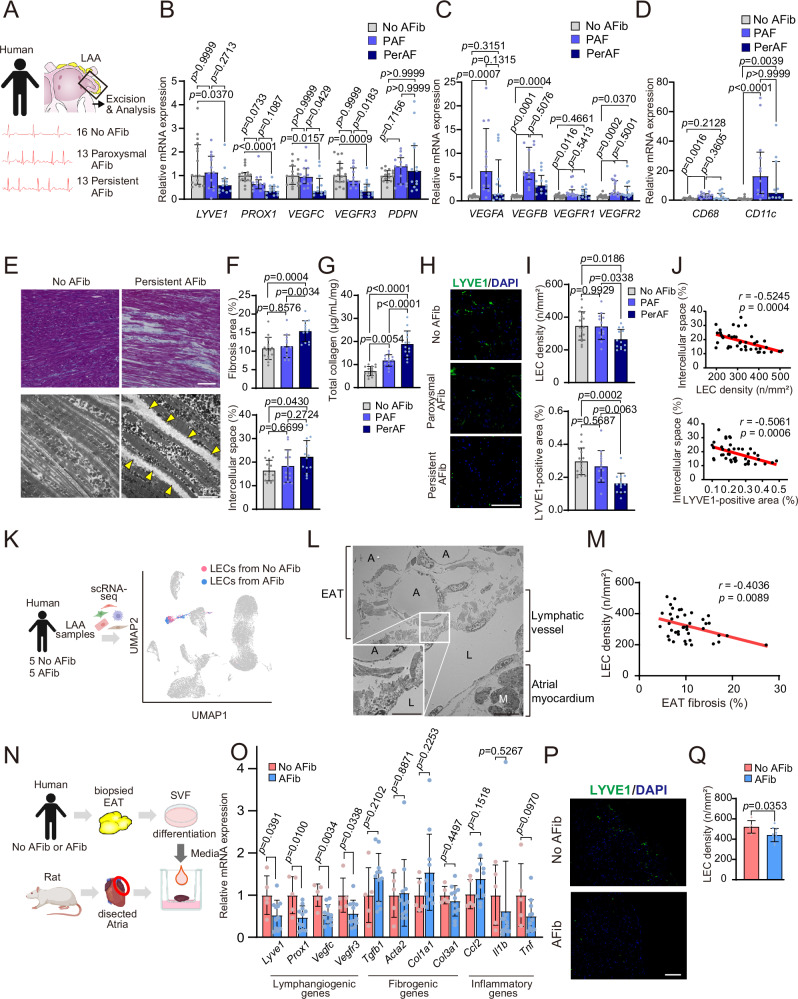
Impaired atrial lymphangiogenesis in human atrial fibrillation. **A** Schematic illustration of the experiments in human left atrial appendage (LAA). LAA excised during open cardiac surgery were analysed. *n* = 16 for No AFib patients, *n* = 13 each for PAF and PerAF patients. Created in BioRender. Takahashi, M. (https://BioRender.com/5uy66i9). **B** Relative mRNA levels of lymphangiogenic genes in LAA from the patients with No AFib, PAF and PerAF. *n* = 16 for No AFib, *n* = 13 each for PAF and PerAF, biologically independent samples. Data are median with IQR. Error bars indicate the IQR.; *p* value was determined by the Kruskal-Wallis test followed by the Dunn’s *post hoc* test. **C** Relative mRNA levels of angiogenic genes in LAA from the patients with No AFib, PAF and PerAF. *n* = 16 for No AFib, *n* = 13 each for PAF and PerAF, biologically independent samples. Data are median with IQR. Error bars indicate the IQR.; *p* value was determined by the Kruskal-Wallis test followed by the Dunn’s *post hoc* test. **D** Relative mRNA levels of inflammatory genes in LAA from the patients with No AFib, PAF and PerAF. *n* = 16 for No AFib, *n* = 13 each for PAF and PerAF, biologically independent samples. Data are median with IQR. Error bars indicate the IQR.; *p* value was determined by the Kruskal-Wallis test followed by the Dunn’s *post hoc* test. **E** Top: Representative Masson’s Trichrome staining in LAA from the patients with No AFib and PerAF. Scale bar, 100 μm. Bottom: Representative transmission electron microscopy image in LAA from the patients with No AFib and PerAF. Scale bar, 5 μm. Yellow arrowheads indicate the intercellular space. **F** Top: Quantification of fibrosis area in LAA. *n* = 16 for No AFib, *n* = 13 each for PAF and PerAF, biologically independent samples. Data are mean ± SD.; *p* value was determined by one-way ANOVA followed by the Tukey-Kramer’s *post hoc* test. ns, not significant. Bottom: Quantification of intercellular space in LAA. *n* = 16 for No AFib, *n* = 13 each for PAF and PerAF, biologically independent samples. Data are mean ± SD.; *p* value was determined by one-way ANOVA followed by the Tukey-Kramer’s *post hoc* test. **G** Quantification of total collagen contents in LAA. *n* = 16 for No AFib, *n* = 13 each for PAF and PerAF, biologically independent samples. Data are mean ± SD.; *p* value was determined by one-way ANOVA followed by the Tukey-Kramer’s *post hoc* test. **H** Representative immunofluorescent staining for LYVE1 (Green)/DAPI (Blue) in LAA from the patients with No AFib, PAF and PerAF. Scale bar, 100 μm. **I** Quantification of LEC density (Top) and LYVE1-positive area (Bottom) in LAA. *n* = 16 for No AFib, *n* = 13 for PAF or PerAF, biologically independent samples. Data are mean ± SD.; *p* value was determined by one-way ANOVA followed by the Tukey-Kramer’s *post hoc* test. **J** Top: Correlation between LEC density and intercellular space in LAA. *n* = 42. *p* value was determined using a two-tailed Pearson correlation test. Bottom: Correlation between LYVE1-positive area and intercellular space in LAA. *n* = 42. *p* value was determined using a two-tailed Pearson correlation test. **K** UMAP from published single-cell RNA sequencing datasets in human left atrial tissues from No AFib (*n* = 5) and persistent AFib (*n* = 5) patients^29^. LECs population: No AFib 5.0%, AFib 2.5%. Partially created in BioRender. Takahashi, M. (https://BioRender.com/f8lw313). **L** Representative transmission electron microscopy image for the lymphatic vessel, atrial cardiomyocytes and peri-atrial EAT in LAA. Scale bar,10 μm. Inset: magnified images. Scale bar, 5 μm. A, adipocyte. L, lymphatic vessel. M, atrial cardiomyocyte. **M** Correlation between EAT-fibrosis and LEC density in LAA. *n* = 42. *p* value was determined using a two-tailed Pearson correlation test. **N** Schematic illustration of the experiments using EAT conditioned media and the organo-cultured rat atria tissues. Created in BioRender. Takahashi, M. (https://BioRender.com/b0vf3en). **O** Relative mRNA levels of lymphangiogenic, fibrogenic and inflammatory genes in rat atrial tissues treated with EAT conditioned media from the patients with No AFib and AFib. *n* = 5 for No AFib, *n* = 11 for AFib, biologically independent samples. Data are mean ± SD.; *p* value was determined by two-tailed unpaired Student’s *t*-test. **P** Representative immunofluorescent staining for LYVE1 (Green)/DAPI (Blue) in rat atrial tissues treated with EAT conditioned media from the patients with No AFib and AFib. Scale bar, 100 μm. **Q** Quantification of LEC density in rat atrial tissues treated with EAT conditioned media from the patients with No AFib and AFib. *n* = 5 for No AFib, *n* = 11 for AFib, biologically independent samples. Data are mean ± SD.; *p* value was determined by two-tailed unpaired Student’s *t*-test.

To investigate the role of lymphangiogenesis in AFib, we analyzed human left atrial appendage (LAA) samples from patients in sinus rhythm (No AFib, *n* = 16), with paroxysmal AFib (PAF, *n* = 13), and with persistent AFib (PerAF, *n* = 13) undergoing heart surgery (Fig. 1A, and Supplementary Table S1). Quantitative RT-PCR analysis revealed significantly decreased expression of lymphangiogenesis-related genes (*lymphatic vessel endothelial hyaluronan receptor 1* (*LYVE1*), *prospero homeobox 1* (*PROX1*), *vascular endothelial growth factor c* (*VEGFC*), *vascular endothelial growth factor receptor 3* (*VEGFR3*)) in PerAF groups compared to No AFib (Fig. 1B). This decrease was progressive, with PerAF samples showing the lowest expression levels (Fig. 1B). Angiogenesis- and inflammation-related genes showed a biphasic pattern, with increased expression in PAF and decreased expression in PerAF (Fig. 1C, D), while NLRP3 inflammasome-related proteins were elevated in PerAF, consistent with previous findings (Supplementary Fig. S1A, B)^27,28^.

Histological analysis using Masson’s trichrome staining and transmission electron microscopy (TEM) revealed enlarged interstitial spaces and increased fibrosis in PerAF samples compared to No AFib (Fig. 1E, F), which was consistent with the quantitative analysis for total collagen (Fig. 1G). Immunohistochemical staining for LYVE1 confirmed reduced lymphatic vessel density in AFib patients, with a significant decrease observed in PerAF groups (Fig. 1H, I). In addition, intercellular space has a significant inverse correlation with lymphatic vessel density (Fig. 1J), suggesting its crucial role in atrial fluid homeostasis. Moreover, we searched for published single-cell RNA sequencing (scRNA-seq) datasets in human left atrial tissue from No AFib and persistent AFib patients (Fig. 1K)^29^. Consistent with our findings, the human lymphatic endothelial cells (LECs) cluster in AFib atria were reduced by half compared to No AFib atria (Fig. 1K, Supplementary Fig. S1C).

To further investigate the structural changes in atrial lymphatic vessels, we conducted detailed TEM analysis of the atrial tissue. Surprisingly, we found that lymphatic vessels were frequently located in close proximity to epicardial adipose tissue (EAT) (Fig. 1L). This unexpected observation led us to examine the relationship between EAT and atrial lymphatic vessels more closely. Analysis of EAT fibrosis, an established indicator of EAT inflammation^30,31^, revealed an inverse correlation with atrial lymphatic vessel density (Fig. 1M), suggesting a potential interaction between EAT remodeling and atrial lymphatic dysfunction. To explore the relationship between EAT and lymphangiogenesis, gene expression patterns were compared between paired EAT and subcutaneous adipose tissue (SAT) samples obtained from the same patients. qRT-PCR analysis revealed higher expression of lymphangiogenesis-related genes in EAT compared to SAT (Supplementary Fig. S1D), while angiogenesis markers remained unchanged (Supplementary Fig. S1E). These findings suggest a potential regulatory role of EAT in atrial lymphangiogenesis, opening new perspectives for understanding the pathophysiology of AFib.

### EAT-secreted factors impair lymphangiogenesis

To investigate the effect of EAT-derived factors on atrial lymphangiogenesis, an organo-culture system using rat atrial tissue was established (Fig. 1N, and Supplementary Table S2)^25,32^. *First*, we verified whether this organo-culture system is appropriate for evaluating lymphangiogenesis over time. The system validation demonstrated that angiotensin II (AngII) treatment increased the expression of *Lyve1*, *Vegfc*, and *Vegfr3* (Supplementary Fig. S2A-C), whereas *Prox1* expression decreased over time (Supplementary Fig. S2D), consistent with previous reports of AngII-induced lymphangiogenesis^18^. Based on these results, a one-week treatment period was determined appropriate for the experimental design and proceeded with our investigations accordingly.

*Second*, serum-free culture media was collected from differentiated human EAT stromal vascular fraction (SVF) derived from No AFib or AFib patients (Fig. 1N). Human EAT-SVF were differentiated in DMEM media containing an adipogenic cocktail^33,34^, whereas we did not find any difference in lipid accumulation or mRNA levels of adipogenic marker genes between the two groups (Supplementary Fig. S2E-G). *Third*, the collected serum-free culture media (No AFib- or AFib-EAT conditioned media) were applied to the epicardial side of the organ-cultured rat atrial tissue (Fig. 1N). After 7 days of culture, atrial tissue exposed to AFib-EAT conditioned media showed significantly reduced expression of lymphangiogenesis-related genes compared to those exposed to No AFib-EAT conditioned media (Fig. 1O). Notably, inflammation- and fibrosis-related genes remained unchanged (Fig. 1O), suggesting a direct effect on lymphatic endothelial cells rather than a general inflammatory response. Immunohistochemical analysis confirmed decreased LYVE1-positive lymphatic vessels in the AFib-EAT group (Fig. 1P, Q).

**Fig. 2:**
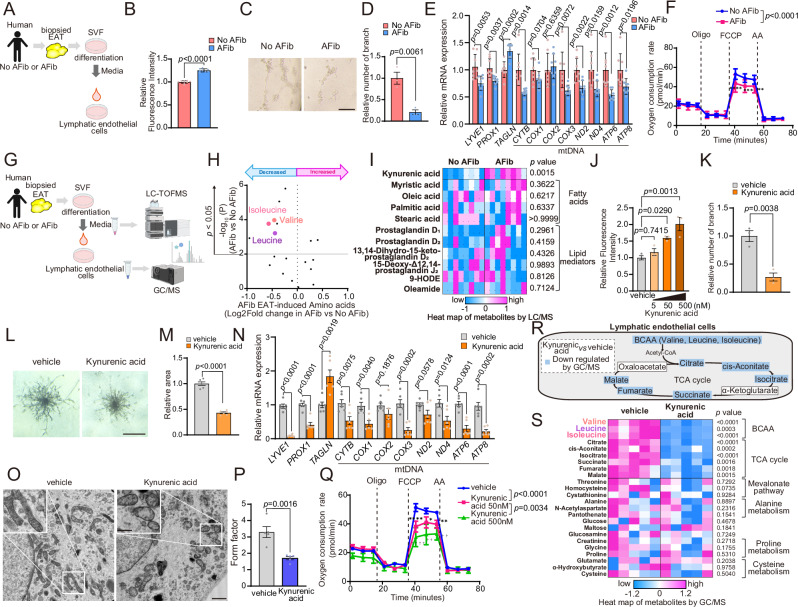
Kynurenic acid mediates EAT-induced lymphatic endothelial dysfunction. **A** Schematic illustration of experiments in LEC treated with EAT conditioned media from the patients with No AFib and AFib. Created in BioRender. Takahashi, M. (https://BioRender.com/v1w6u45). **B** Quantification of permeabilization for LECs treated with EAT conditioned media from the patients with No AFib and AFib. *n* = 8 for both groups, biologically independent samples. Data are mean ± SD.; *p* value was determined by two-tailed unpaired Student’s *t*-test. **C** Representative image of tube formation of LECs treated with EAT conditioned media from the patients with No AFib and AFib. Scale bar, 200 μm. **D** Relative number of branch formed by LEC treated with EAT conditioned media from the patients with No AFib and AFib. *n* = 3 for both groups, biologically independent samples. Data are mean ± SD.; *p* value was determined by two-tailed unpaired Student’s *t*-test. **E** Relative mRNA levels of lymphatic endothelial markers, mesenchymal markers, and mitochondria-encoded genes in LECs treated with EAT conditioned media from the patients with No AFib and AFib. *n* = 8 for both groups, biologically independent samples. Data are mean ± SD.; *p* value was determined by two-tailed unpaired Student’s *t*-test. **F** Changes in oxygen consumption rate (OCR) in LECs treated with No AFib or AFib-EAT conditioned media. Cells were treated with 2.5 μM oligomycin followed by phenylhydrazone (FCCP, 2 μM), and antimycin (0.5 μM). *n* = 9 for both groups, biologically independent samples. Data are mean ± SD.; *p* value was determined by two-way repeated-measures ANOVA followed by Sidak’s multiple comparison test. ** *p* < 0.01, *** *p* < 0.001, **** *p* < 0.0001 (vs No AFib). **G** Schematic illustration of metabolomics analysis. Metabolites contained in EAT conditioned media from the patients with No AFib and AFib were analysed by LC-TOFMS analysis. LECs treated with EAT conditioned media from the patients with No AFib and AFib were analysed by GC/MS analysis. Created in BioRender. Takahashi, M. (https://BioRender.com/kxkxn98). **H** GC/MS-based volcano plot for both increased and decreased intercellular amino acids from comparison of No AFib- or AFib-EAT treated LECs. **I** Heat-map of metabolites by LC-TOFMS in EAT conditioned media from the patients with No AFib and AFib. The color scale shows *Z*-scored signal intensity representing the signal intensity of each metabolite in the blue (low expression)-white-red (high expression) scheme. *n* = 8 for both groups, biologically independent samples. *p*-value was determined by two-tailed unpaired Student’s *t*-test. **J **Quantification of permeabilization for LECs treated with vehicle or Kynurenic acid (5 nM, 50 nM and 500 nM). *n* = 3 for each group, biologically independent samples. Data are mean ± SEM.; *p* value was determined by one-way ANOVA followed by the Dunnett’s *post hoc* test. **K** Relative number of branch formed by LECs treated with vehicle or Kynurenic acid (50 nM). *n* = 3 for both groups, biologically independent samples. Data are mean ± SEM.; *p* value was determined by two-tailed unpaired Student’s *t*-test. **L**. Representative bright-field images of LECs spheroids embedded in invasion matrix treated with vehicle or Kynurenic acid. Scale bar, 500 μm. **M** Quantitative analysis of area in (**L**). *n *= 6 for both groups, biologically independent samples. Data are mean ± SEM.; p value was determined by two-tailed unpaired Student’s *t*-test. **N**. Relative mRNA levels of lymphatic endothelial markers, mesenchymal markers, and mitochondria-encoded genes in LECs treated with vehicle or Kynurenic acid (50 nM). *n* = 6 for both groups, biologically independent samples. Data are mean ± SEM.; *p* value was determined by two-tailed unpaired Student’s *t*-test. **O** Representative transmission electron microscopy image for the LEC treated by vehicle or Kynurenic acid (50 nM). Scale bars: 1 μm. Inset: magnified images of the mitochondria. Scale bar, 0.5 μm. **P** Quantification of mitochondrial Form factor (perimeter^2^/4π area, combined measure of length and degree of branching) in LECs treated by vehicle or Kynurenic acid (50 nM). *n* = 5 for both groups, biologically independent samples. Data are mean ± SEM.; *p* value was determined by two-tailed unpaired Student’s *t*-test. **Q** Changes in OCR in LECs treated with vehicle or Kynurenic acid (50, and 500 nM). Cells were treated with 2.5 μM oligomycin followed by phenylhydrazone (FCCP, 2 μM), and antimycin (0.5 μM). *n* = 3 for both groups, biologically independent samples. Data are mean ± SEM.; *p* value was determined by two-way repeated-measures ANOVA followed by Sidak’s multiple comparison test. * *p* < 0.05, ** *p* < 0.01, **** *p* < 0.0001 (*vs* vehicle), and ^††^*p* < 0.01, ^††††^*p* < 0.0001 (*vs* vehicle). **R** Schematic illustration of the TCA cycle related metabolites in LECs analysed by GC/MS. Metabolites downregulated by Kynurenic acid were represented in blue. BCAA, branched chain amino acid. **S** Heat-map of metabolites by GC/MS in LECs treated by vehicle or Kynurenic acid. The color scale shows *Z*-scored signal intensity representing the signal intensity of each metabolite in the blue (low expression)-white-red (high expression) scheme. *n* = 5 for both groups, biologically independent samples. *p*-value was determined by two-tailed unpaired Student’s *t*-test.

To further elucidate the mechanisms underlying EAT-mediated lymphatic impairment, in vitro experiments were performed using human LECs (Fig. 2A, and Supplementary Table S3). Using the dextran assay protocol, we attempted to measure paracellular permeability in LECs upon treatment with No AFib- or AFib-EAT conditioned media. Enhanced endothelial permeability is frequently marked by an increase in transfer of dextran across the cells (Supplementary Fig. S3A). After 4 days treatment of LECs with EAT conditioned media, paracellular permeability measured by exposing cells to FITC-dextran indicated that AFib-EAT conditioned media increased LECs permeability compared to No AFib-EAT conditioned media (Fig. 2B), suggesting impaired barrier function of lymphatic vessels. To further assess the functional impairment of LECs, tube formation capacity, a characteristic feature of endothelial cells^35^, was evaluated. Using tube formation assay protocol, the tube forming ability of LECs was analyzed upon treatment with No AFib or AFib-EAT conditioned media. AFib-EAT conditioned media decreased the tube forming ability of LECs, compared to No AFib conditioned media (Fig. 2C, D). Additionally, AFib-EAT conditioned media impaired LEC proliferation, reduced invasive capacity, and decreased migratory properties compared to No AFib-EAT conditioned media (Supplementary Fig. S3B-G). qRT-PCR analysis revealed that LECs exposed to AFib-EAT conditioned media showed reduced expression of lymphatic endothelial markers, *LYVE1*, *PROX1*, and increased expression of mesenchymal marker, *transgelin* (*TAGLN*), compared to those exposed to No AFib-EAT conditioned media, suggesting the loss of LEC identity and endothelial-to-mesenchymal transition (EndMT) (Fig. 2E). These findings were further validated by immunofluorescence staining and western blot analysis, which confirmed decreased PROX1 protein expression and increased TAGLN protein expression in LECs treated with AFib-EAT conditioned media (Supplementary Fig. S3H–K). These results are consistent with previous reports of LECs EndMT^35^. Taken together, we have concluded that LECs undergo EndMT upon AFib-EAT conditioned media treatment with loss of endothelial and gain of mesenchymal characteristics, leading to compromised lymphatic vessel integrity and function.

### Kynurenic acid mediates EAT-induced lymphatic dysfunction

Notably, the qRT-PCR analysis also found that LECs exposed to AFib-EAT conditioned media expressed significantly lower levels of mitochondria-encoded genes, such as *cytochrome b* (*CYTB*), and *cytochrome c oxidase subunit 1* (*COX1*), *nadh dehydrogenase subunit 2* (*ND2*)*, nadh dehydrogenase subunit 4* (*ND4*)*, atp synthase f0 subunit 6* (*ATP6*), *atp synthase f0 subunit 8* (*ATP8*), compared to those exposed to No AFib-EAT conditioned media (Fig. 2E). These molecular findings were corroborated by functional assessments showing that AFib-EAT conditioned media significantly reduced oxygen consumption rate in LECs (Fig. 2F). Additionally, mitochondrial membrane potential was decreased and mitochondrial ROS production was increased in AFib-EAT conditioned media-treated LECs compared to controls (Supplementary Fig. S3L), confirming compromised mitochondrial homeostasis. Thus, to uncover the mechanism by which AFib-EAT conditioned media impaired LECs function, metabolomic analysis using gas chromatography-mass spectrometry (GC/MS) was performed to examine primary metabolites in LECs (Fig. 2G). This analysis uncovered a significant decrease in branched-chain amino acids (BCAA; valine, leucine and isoleucine) in LECs exposed to AFib-EAT media, suggesting a disruption in cellular energy metabolism, which could contribute to lymphatic endothelial cell dysfunction. (Fig. 2H). To identify the specific mediators of EAT-induced lymphatic dysfunction, we next conducted a comprehensive metabolite profiling of No AFib-EAT and AFib-EAT conditioned media using liquid chromatography time-of-flight mass spectrometry (LC-TOFMS) (Fig. 2I). This analysis revealed significantly higher levels of kynurenic acid in AFib-EAT media compared to No AFib-EAT media (Fig. 2I), while fatty acids and lipid mediators showed no significant differences between the two conditions. To confirm the role of kynurenic acid, LECs were treated with kynurenic acid at concentrations centered around 50 nM, which corresponds to physiological blood levels^36–38^. Remarkably, kynurenic acid treatment recapitulated the effects observed with AFib-EAT conditioned media. Kynurenic acid treatment increased LECs permeability in a concentration-dependent manner (Fig. 2J). In addition, kynurenic acid treatment decreased the tube forming ability of LEC (Fig. 2K), impaired LEC proliferation, reduced invasive capacity, and decreased migratory properties (Fig. 2L, M, Supplementary Fig. S4A–C). LECs exposed to kynurenic acid showed altered gene expression of *LYVE1*, *PROX1*, and *TAGLN*, which was consistent with EndMT (Fig. 2N). Protein-level analysis corroborated these findings, with immunofluorescence staining and western blot demonstrating increased TAGLN and decreased PROX1 expression in kynurenic acid-treated LECs (Supplementary Fig. S4D–G). Mechanistically, kynurenic acid treatment increased phosphorylation of SMAD2/3, TAK1, ERK1/2, and p38MAPK, and SMAD2/3 inhibition effectively prevented kynurenic acid-induced EndMT marker changes, whereas TAK1 inhibition had no effect (Supplementary Fig. S4H-J). These findings suggest that the canonical TGF-β/SMAD2/3 signaling pathway may be the predominant mechanism by which kynurenic acid promotes EndMT. Notably, kynurenic acid treatment did not affect cardiomyocyte and cardiac fibroblast genes expression (Supplementary Fig. S4K, L).

Of note, kynurenic acid treatment significantly decreased mRNA levels of mitochondria-encoded genes, such as *CYTB*, and *COX1*, *cytochrome c oxidase subunit 3* (*COX3*)*, ND2, ND4, ATP6*, *ATP8* (Fig. 2N). TEM analysis found that LECs harbored numerous mitochondria with dense cristae (Fig. 2O, left panel). In contrast, mitochondria in kynurenic acid-treated LECs were generally shortened, swollen and less dense, further supporting the notion of mitochondrial dysfunction (Fig. 2O, right panel). Quantitative analysis of mitochondrial shape complexity using the form factor F^39,40^ revealed that kynurenic acid-treated LECs displayed significantly lower values, indicating simpler shapes, compared to vehicle-treated controls (Fig. 2 P). Consistent with these morphological changes, kynurenic acid treatment resulted in reduced oxygen consumption rate, decreased mitochondrial membrane potential, and increased mitochondrial ROS production (Fig. 2Q, and Supplementary Fig. S4M), confirming functional impairment of mitochondrial homeostasis. Thus, we next performed a metabolomic analysis using GC/MS. Mechanistically, kynurenic acid treatment led to decreased intracellular levels of branched-chain amino acids (BCAA; valine, leucine and isoleucine) and tricarboxylic acid (TCA) cycle metabolites (Citrate, cis-Aconitate, Isocitrate, Succinate, Fumarate and Malate) in LECs, as determined by GC/MS analysis (Fig. 2R, S). On the other hand, other metabolites including threonine, alanine, glycine, proline, glutamate, cysteine remained unchanged (Fig. 2S). To directly investigate whether metabolic dysfunction promotes EndMT, we cultured LECs in BCAA-depleted medium and found that BCAA depletion progressively downregulated *LYVE1* and *PROX1* expression while upregulating *TAGLN* expression in a time-dependent manner (Supplementary Fig. S5A). These findings strongly suggest that the metabolic effects of kynurenic acid are specifically targeted towards BCAA and TCA cycle intermediates and mitochondrial energy metabolism, rather than inducing a global alteration in cellular metabolism, and that this targeted metabolic dysfunction directly promotes EndMT, leading to lymphatic endothelial cell dysfunction.

The elevated kynurenic acid was intriguing as a previous study demonstrated that kynurenic acid via G protein-coupled receptor Gpr35 pathway mediated energy expenditure in mice^41,42^. Notably, immunohistochemical staining for LYVE1/GPR35 confirmed increased GPR35-positive lymphatic vessel in AFib patients (Supplementary Fig. S5B, C), AFib-EAT conditioned media significantly upregulated *GPR35* mRNA expression in LECs compared to No AFib-EAT conditioned media, and chronic kynurenic acid treatment (50 and 500 nM for 4 days) further enhanced *GPR35* expression (Supplementary Fig. S5D, E). Accordingly, LECs were incubated with kynurenic acid in the presence or absence of Gpr35 inhibitors, ML145 or CID2745687. Subsequently, we employed GC/MS and quantified BCAA; valine, leucine and isoleucine contents in LECs. GC/MS analysis revealed that kynurenic acid clearly reduced intracellular levels of BCAA; valine, leucine and isoleucine in a dose-dependent manner (Supplementary Fig. S5F). This effect was abrogated by both Gpr35 inhibitors (ML145 and CID2745687) (Supplementary Fig. S5F), suggesting that kynurenic acid alters cellular energy metabolism via Gpr35 in LECs.

### AngII-induced impaired lymphangiogenesis promotes AFib in vivo

Building upon our human LAA samples, organo-culture, and in vitro findings suggesting a role for lymphatic dysfunction in AFib susceptibility, subsequent investigations examined this hypothesis in vivo.

**Fig. 3:**
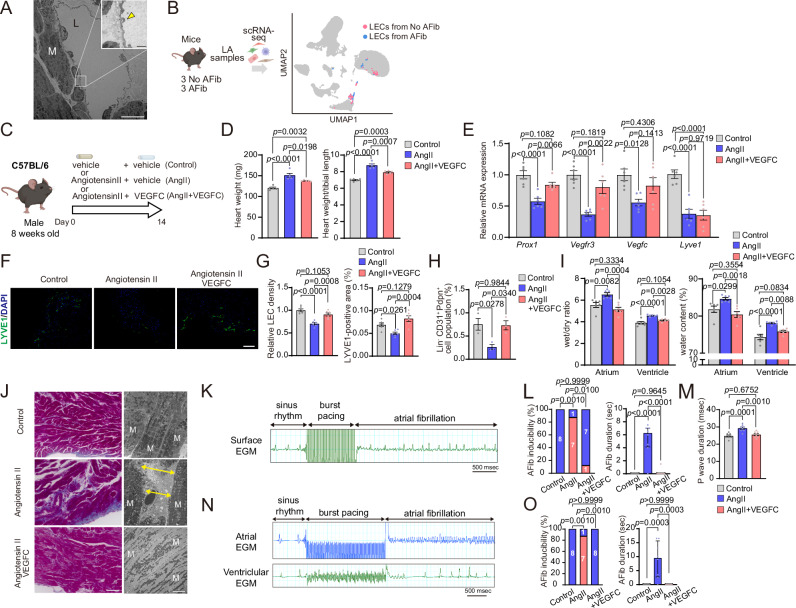
Angiotensin II impairs atrial lymphangiogenesis and increases atrial fibrillation susceptibility. **A** Representative transmission electron microscopy image for the mice LECs. Similar ultrastructural features were observed in five biologically independent mice. Scale bars: 10 μm. Inset: magnified image. Scale bar, 1 μm. Yellow arrowheads indicate the button-like junction. L, lymphatic vessel. M, atrial cardiomyocyte. **B** UMAP from published single-cell RNA sequencing datasets in mice left atrial tissues from No AFib (*n* = 3) and AFib (*n* = 3, combining of angiotensin II, high-fat diet, and mitral valve regurgitation) models^29^. LECs population: No AFib 1.2%, AFib 0.5%. Partially created in BioRender. Takahashi, M. (https://BioRender.com/81qzk8d). **C** Schematic illustration of the experiment procedure in mice. Male C57BL/6J mice at 8 weeks old received vehicle, Angiotensin II and/or VEGFC by osmotic pump for 14 days. Created in BioRender. Takahashi, M. (https://BioRender.com/kmfm2vr). **D** Changes in heart weight (left) and Heart weight/Tibial length (right) treated with Angiotensin II and/or VEGFC for 14 days. *n* = 6 *per* group, biologically independent mice. Data are mean ± SEM.; *p* value was determined by one-way ANOVA followed by the Tukey-Kramer’s *post hoc* test. **E** Relative mRNA levels of lymphangiogenic genes in left atria from vehicle, Angiotensin II and/or VEGFC treated mice. *n* = 6 *per* group, biologically independent mice. Data are mean ± SEM.; *p* value was determined by one-way ANOVA followed by the Tukey-Kramer’s *post hoc* test. **F** Representative immunofluorescent staining for LYVE1 (Green)/DAPI (Blue) in left atria from vehicle, Angiotensin II and/or VEGFC treated mice. Scale bar, 100 μm. **G** Quantification of relative LEC density (left) and LYVE1-positive area (right) in left atria from vehicle, Angiotensin II and/or VEGFC treated mice. *n* = 6 *per* group, biologically independent mice. Data are mean ± SEM.; *p* value was determined by one-way ANOVA followed by the Tukey-Kramer’s *post hoc* test. **H** FACS-based quantification of LECs (Lin^-^: Cd31^+^: Pdpn^+^ cells) in mice atria treated with vehicle, Angiotensin II and/or VEGFC. *n* = 3 *per* group, biologically independent mice. Data are mean ± SEM.; *p* value was determined by one-way ANOVA followed by the Tukey-Kramer’s *post hoc* test. **I** Wet/Dry ratio (left) and % water contents of mice atrium and ventricle. *n* = 6 *per* group, biologically independent mice. Data are mean ± SEM.; *p* value was determined by one-way ANOVA followed by the Tukey-Kramer’s *post hoc* test. **J** Left: Representative Masson’s Trichrome staining for atrium from vehicle, AngiotensinII and/or VEGFC treated mice. Scale bar, 50 μm. Right: Representative transmission electron microscopy image for atrium from vehicle, Angiotensin II and/or VEGFC treated mice. Similar ultrastructural features were observed in at least three biologically independent mice per group. Scale bar, 5 μm. Yellow double-headed arrows indicate the widened intercellular space. **K** Representative electrocardiogram of atrial fibrillation induced in Angiotensin II treated mice by transesophageal burst pacing. Scale bar, 500 msec. **L** Left: Atrial fibrillation inducibility by transesophageal burst pacing. *n* = 8 *per* group, biologically independent mice, analysed by two-tailed Fisher’s exact test. Right: Atrial fibrillation duration induced by transesophageal pacing. *n* = 8 *per* group, biologically independent mice. Data are median with IQR. Error bars indicate the IQR.; *p* value was determined by the Kruskal-Wallis test followed by the Dunn’s *post hoc* test. **M** *P* wave duration during sinus rhythm. *n* = 8 *per* group, biologically independent mice. Data are mean ± SEM.; *p* value was determined by one-way ANOVA followed by the Tukey-Kramer’s *post hoc* test. **N** Representative atrial electrogram (Top) and ventricle electrogram (Bottom) of atrial fibrillation induced in Angiotensin II treated mice by burst pacing. Scale bar, 500 msec. **O** Left: Atrial fibrillation inducibility by burst pacing. *n* = 8 *per* group, biologically independent mice, analysed by two-tailed Fisher’s exact test. Right: Atrial fibrillation duration induced by transesophageal pacing. *n* = 8 *per* group, biologically independent mice. Data are median with IQR. Error bars indicate the IQR.; *p* value was determined by the Kruskal–Wallis test followed by the Dunn’s *post hoc* test.

*First*, TEM analysis confirmed that the structure of atrial lymphatic vessels in mice, including their characteristic button-like junctions^43,44^, closely resembles that observed in humans (Fig. 3A). *Second*, we searched for published scRNA-seq datasets in mice left atrial tissue from No AFib and AFib models (Fig. 3B)^29^. In AFib atria, the LECs cluster were reduced by half compared to No AFib atria (Fig. 3B, and Supplementary Fig. S6A). Since this published model combined angiotensin II (AngII) treatment and high-fat diet, further investigation aimed to dissect the individual contributions of these stressors to better understand the relationship between lymphatic endothelial cells and AFib, as well as to identify potential therapeutic targets. To this end, we utilized two well-established models known to increase AFib susceptibility: AngII infusion and high-fat diet-induced obesity. These models allowed us to examine the dynamics of atrial lymphatics under conditions that predispose to AFib and to explore potential therapeutic interventions.

An AngII infusion two-week model was employed to induce a pro-arrhythmic state (Fig. 3C, and Supplementary Table S4). AngII infusion increased heart weight and heart weight/tibial length (Fig. 3D). Analysis of atrial mRNA expression revealed a significant downregulation of lymphangiogenesis-related genes, including *Prox1*, *Vegfr3*, *Vegfc* and *Lyve1* after one to two weeks of AngII infusion, while CaMKII signaling and calcium handling remained unchanged at one week (Fig. 3E, and Supplementary Fig S6B–D). To investigate whether promoting lymphangiogenesis could mitigate the AngII-induced changes, vascular endothelial growth factor C (VEGFC) was administered to a subset of AngII-infused mice (Fig. 3C). VEGFC treatment for two weeks led to decreased heart weight and heart weight/tibial length (Fig. 3D), increased expression of lymphangiogenesis-related genes (Fig. 3E). Immunohistochemical analysis confirmed decreased lymphatic vessel density by AngII infusion, and increased lymphatic vessel density by VEGFC treatment (Fig. 3F, G). Flow cytometry analysis of LEC population (Lin^-^: Cd31^+^: Pdpn^+^ cells) confirmed a decrease with AngII infusion and an increase with VEGFC administration (Fig. 3H, Supplementary Fig S6E). Concomitantly, an increased heart wet-to-dry weight ratio and water content were observed in AngII-infused hearts, which was significantly reduced with VEGFC treatment, indicating improved regulation of cardiac fluid balance in VEGFC-treated mice (Fig. 3I), which was consistent with histological analysis and TEM (Fig. 3J). While these alterations were observed in both atrial and ventricular tissues, the wet-to-dry weight ratio and water content were significantly elevated in the atrium relative to the ventricle (Fig. 3I). This disparity suggests that lymphangiogenesis plays a more critical role in fluid homeostasis within atrial tissue compared to ventricular tissue. In addition, VEGFC treatment reduced the AngII-induced inflammation and fibrosis, as evidenced by qRT-PCR analysis and histological analysis (Supplementary Fig. S7A–C), while CaMKII signaling, calcium handling, and NLRP3 inflammasome markers remained unchanged (Supplementary Fig. S7D–I).

Importantly, comprehensive electrophysiological studies were conducted to assess the impact of VEGFC treatment on AFib susceptibility and duration. In vivo studies were performed using transesophageal catheterization under light anesthesia, and induced AFib by burst pacing (Fig. 3K). In vivo electrophysiological studies revealed that AngII-infused mice exhibited significantly increased AFib inducibility compared to control mice (Fig. 3L). Strikingly, VEGFC treatment markedly reduced AFib inducibility in AngII-infused mice to near-control levels (Fig. 3L). Moreover, when AFib was successfully induced, its duration was substantially prolonged in AngII-infused mice, and VEGFC treatment significantly reduced AFib duration (Fig. 3L). P wave duration, which serve as a surrogate for atrial conduction velocity^45^, was also increased by AngII infusion, and decreased by VEGFC treatment (Fig. 3M).

Thus, we next asked if an increased susceptibility to AFib occurs in langendorff-perfused hearts, a condition in which potential confounding systemic factors were eliminated (Fig. 3N). To this end, ex vivo studies in Langendorff-perfused hearts corroborated these findings, demonstrating similar trends in AFib inducibility and duration across all groups (Fig. 3O). This consistency between in vivo and ex vivo results strongly suggests that the observed anti-arrhythmic effects of VEGFC are primarily mediated through local cardiac mechanisms rather than systemic factors.

Collectively, these comprehensive electrophysiological findings provide robust evidence that VEGFC treatment significantly reduces AFib susceptibility and duration in the setting of AngII-induced atrial remodeling. The consistency of results across in vivo and ex vivo studies strongly support the therapeutic potential of targeting lymphangiogenesis in AFib prevention and treatment.

**Fig. 4:**
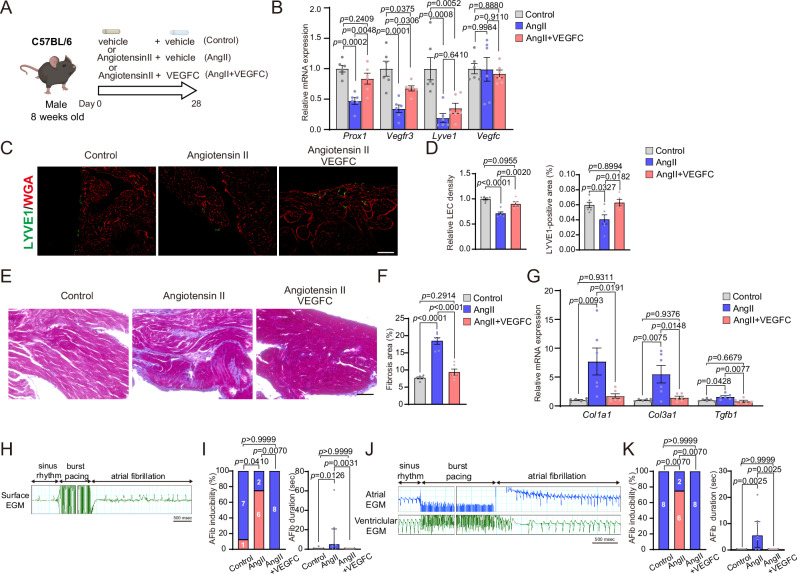
VEGFC restores lymphangiogenesis and suppresses atrial fibrillation. **A** Schematic illustration of the experiment procedure in mice. Male C57BL/6J mice at 8 weeks old received vehicle, Angiotensin II and/or VEGFC by osmotic pump for 28 days. Created in BioRender. Takahashi, M. (https://BioRender.com/2s31w47). **B** Relative mRNA levels of lymphangiogenic genes in left atria from vehicle, Angiotensin II and/or VEGFC treated mice. *n* = 6 *per* group, biologically independent mice. Data are mean ± SEM.; *p* value was determined by one-way ANOVA followed by the Tukey-Kramer’s *post hoc* test. **C** Representative immunofluorescent staining for LYVE1 (Green)/WGA (Red) in left atria from vehicle, Angiotensin II and/or VEGFC treated mice. Scale bar, 100 μm. **D** Quantification of relative LEC density (Left) and LYVE1-positive area (Right) in left atria from vehicle, Angiotensin II and/or VEGFC treated mice. *n* = 6 *per* group, biologically independent mice. Data are mean ± SEM.; *p* value was determined by one-way ANOVA followed by the Tukey-Kramer’s *post hoc* test. **E** Representative Masson’s Trichrome staining for atrium from vehicle, Angiotensin II and/or VEGFC treated mice. Scale bar, 50 μm. **F** Quantification of the atrial fibrosis area in left atria from vehicle, Angiotensin II and/or VEGFC treated mice. *n* = 6 *per* group, biologically independent mice. Data are mean ± SEM.; *p* value was determined by one-way ANOVA followed by the Tukey-Kramer’s *post hoc* test. **G** Relative mRNA levels of fibrogenic genes in left atria from vehicle, Angiotensin II and/or VEGFC treated mice. *n* = 6 *per* group, biologically independent mice. Data are mean ± SEM.; *p* value was determined by one-way ANOVA followed by the Tukey-Kramer’s *post hoc* test. **H** Representative electrocardiogram of atrial fibrillation induced in Angiotensin II treated mice by transesophageal burst pacing. Scale bar, 500 msec. **I** Left: Atrial fibrillation inducibility by transesophageal burst pacing. *n* = 8 *per* group, biologically independent mice, analysed by two-tailed Fisher’s exact test. Right: Atrial fibrillation duration induced by transesophageal pacing. *n* = 8 *per* group, biologically independent mice. Data are median with IQR. Error bars indicate the IQR.; *p* value was determined by the Kruskal-Wallis test followed by the Dunn’s *post hoc* test. **J** Representative atrial electrogram (Top) and ventricle electrogram (Bottom) of atrial fibrillation induced in Angiotensin II treated mice by burst pacing. Scale bar, 500 msec. **K** Left: Atrial fibrillation inducibility by burst pacing. *n* = 8 *per* group, biologically independent mice, analysed by two-tailed Fisher’s exact test. Right: Atrial fibrillation duration induced by transesophageal pacing. *n* = 8 *per* group. Data are median with IQR. Error bars indicate the IQR.; *p* value was determined by the Kruskal-Wallis test followed by the Dunn’s *post hoc* test.

To further elucidate the temporal dynamics of AngII-induced changes and to investigate whether the observed effects were sustained or exacerbated over time, we extended the AngII infusion period to four weeks in a subset of animals (Fig. 4A). Consistent with our findings at the two-week timepoint, prolonged AngII infusion for four weeks resulted in persistent downregulation of lymphangiogenesis-related genes in the atrial myocardium (Fig. 4B). These molecular changes were corroborated by immunohistochemical analysis, which demonstrated a significant reduction in LYVE1-positive lymphatic endothelial cell density compared to control mice (Fig. 4C, D). We also assessed the progression of fibrosis at this extended timepoint. Masson’s trichrome staining revealed a marked increase in fibrosis in the atria of AngII-infused mice at four weeks, mirroring our observations at the two-week timepoint (Fig. 4E). Quantitative analysis of the stained sections showed a significant expansion of fibrotic areas compared to control (Fig. 4F). Concurrently, qRT-PCR analysis of fibrosis-related genes, including *collagen type I alpha 1* (*Col1a1*), *collagen type III alpha 1* (*Col3a1*), and *transforming growth factor beta* (*Tgfb*), demonstrated sustained upregulation in the AngII-infused group, indicating ongoing fibrotic remodeling (Fig. 4G).

Notably, the administration of VEGFC during this extended infusion period continued to exert protective effects across multiple parameters. VEGFC treatment led to a significant upregulation of lymphangiogenesis-related gene expression (Fig. 4B), and a concomitant increase in lymphatic endothelial cell numbers, as evidenced by LYVE1 immunostaining (Fig. 4C, D). Furthermore, VEGFC administration substantially attenuated the AngII-induced fibrotic response. Masson’s trichrome staining showed a marked reduction in fibrosis in VEGFC-treated mice compared to those receiving AngII alone (Fig. 4E, F). This was accompanied by a significant downregulation of fibrosis-related gene expression (Fig. 4G). These findings suggest that the lymphangiogenic and anti-fibrotic responses to VEGFC remain robust even in the face of prolonged AngII-induced stress. To assess the functional implications of these structural and molecular changes, we evaluated AFib inducibility at the four-week timepoint using both in vivo and ex vivo protocols. In line with our earlier observations, prolonged AngII infusion resulted in a marked increase in AFib susceptibility (Fig. 4H–K). Importantly, VEGFC administration significantly attenuated this pro-arrhythmic effect, reducing AFib inducibility to levels comparable to those observed in control mice (Fig. 4I, K).

Taken together, these results demonstrate that the detrimental effects of AngII on atrial lymphatics and AFib susceptibility persist and potentially intensify with prolonged exposure. Furthermore, our findings underscore the sustained efficacy of VEGFC in counteracting these effects, even in the context of extended AngII-induced stress. These observations not only reinforce the link between lymphatic dysfunction and AFib pathogenesis but also highlight the potential long-term benefits of lymphangiogenic therapy in AFib prevention.

### Obesity-induced impaired lymphangiogenesis promotes AFib in vivo

Following our investigations with the AngII model, we sought to explore the relationship between atrial lymphatics and AFib susceptibility in a clinically relevant model of obesity-induced AFib. The association between obesity and increased AFib risk is well-established in humans^46,47^, as is the potential for weight loss to reduce AFib burden^48^. To this end, a high-fat diet (HFD) model was employed to induce obesity, followed by a weight loss intervention.

**Fig. 5:**
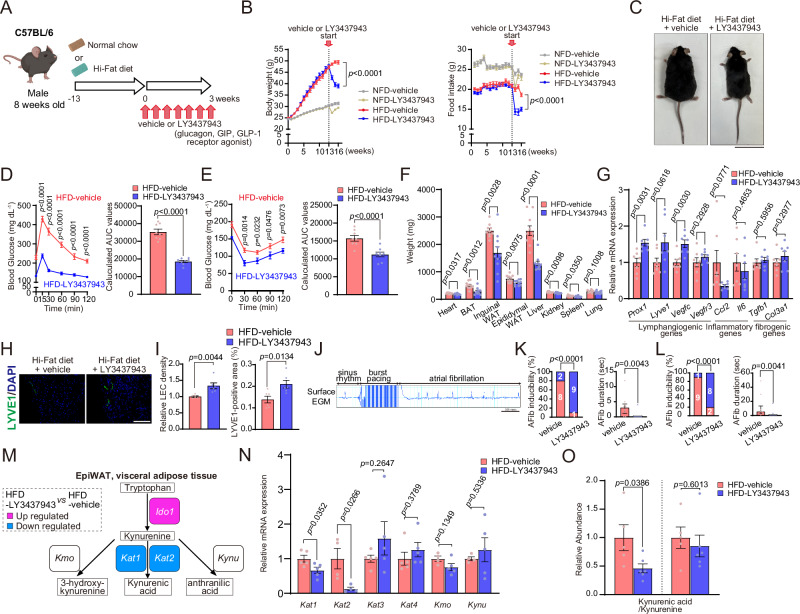
Weight loss improves lymphangiogenesis and reduces atrial fibrillation susceptibility. **A** Schematic illustration of the experiment for HFD-induced obese mice followed by administration of LY3437943. Male C57BL/6J mice at 8 weeks old were given access to normal chow or High-fat diet (60 % fat) for 13 weeks. A subset of mice was then injected LY3437943 intraperitoneally every third day for 3 weeks. Created in BioRender. Takahashi, M. (https://BioRender.com/kc11e0c). **B** Changes in body weight and food intake in each group. Body weight sample sizes were *n* = 20 (NFD-vehicle), *n* = 18 (HFD- vehicle), *n* = 19 (NFD-LY3437943), and *n* = 20 (HFD- LY3437943), biologically independent mice. Food intake sample sizes were *n* = 20 (NFD-vehicle), *n* = 19 (HFD- vehicle), *n* = 20 (NFD-LY3437943), and *n* = 20 (HFD- LY3437943), biologically independent mice.Data are mean ± SEM.; *p* value was determined by two-way repeated-measures ANOVA followed by two-tailed unpaired Student’s *t*-test. **C** Representative image of mice fed High-Fat diet with or without LY3437943 treatment. Scale bar, 5 cm **D** Left: Glucose tolerance test in HFD-induced obese mice with or without LY3437943 treatment. After 6 h of fasting, mice received i.p. injection of glucose at 1.0 g kg^−1^ body weight. *n* = 10 *per* group, biologically independent mice. Data are mean ± SEM.; *** *p* < 0.001, **** *p* < 0.0001, by two-tailed unpaired Student’s *t*-test. Right: Area under the curve (AUC) was determined by Prism software. *p* value was determined by two-tailed unpaired Student’s t test. **E** Left: Insulin tolerance test in HFD-induced obese mice with or without LY3437943 treatment. After 3 hours of fasting, mice received i.p. injection of insulin at1.0 U kg^−1^ body weight. *n* = 10 *per* group, biologically independent mice. Data are mean ± SEM.; *** *p* < 0.001, by two-tailed unpaired Student’s *t*-test. Right: Area under the curve (AUC) was determined by Prism software followed by two-tailed unpaired Student’s t test. **F** Changes in tissue weight in HFD-induced obese mice with or without LY3437943 treatment. *n* = 10 *per* group, biologically independent mice. Data are mean ± SEM.; *p* value was determined by two-tailed unpaired Student’s *t*-test. **G**. Relative mRNA levels of genes in left atria from obese mice with or without LY3437943 treatment. *n* = 7 *per* group, biologically independent mice. Data are mean ± SEM.; *p* value was determined by two-tailed unpaired Student’s *t*-test. **H** Representative immunofluorescent staining for LYVE1 (Green)/DAPI (Blue) in left atria from HFD-induced obese mice with or without LY3437943 treatment. Scale bar, 100 μm. **I** Quantification of relative LEC density (left) and LYVE1-positive area (right) in left atria from HFD-induced obese mice with or without LY3437943. *n* = 6 *per* group, biologically independent mice. Data are mean ± SEM.; *p* value was determined by two-tailed unpaired Student’s *t*-test. **J** Representative electrocardiogram of atrial fibrillation induced in obese mice by transesophageal burst pacing. Scale bar, 500 msec. **K** Left: Atrial fibrillation inducibility by transesophageal burst pacing. *n* = 10 *per* group, biologically independent mice, analysed by Fisher’s exact test. Right: Atrial fibrillation duration induced by transesophageal pacing. *n* = 10 *per* group, biologically independent mice. Data are median with IQR. Error bars indicate the IQR.; *p* value was determined by two-tailed Mann Whitney U test. **L** Left: Atrial fibrillation inducibility by burst pacing. *n* = 10 *per* group, biologically independent mice, analysed by Fisher’s exact test. Right: Atrial fibrillation duration induced by burst pacing. *n* = 10 *per* group, biologically independent mice. Data are median with IQR. Error bars indicate the IQR.; *p* value was determined by two-tailed Mann Whitney U test. **M** Schematic illustration of RT-PCR analysis related to kynurenic acid metabolism in EpiWAT, visceral adipose tissue. Upregulated genes in HFD-LY3437943 adipose tissue relative to HFD-vehicle adipose tissue were shown in red, and downregulated genes were shown in blue. EpiWAT, epididymal white adipose tissue. **N** Relative mRNA levels of kynurenic acid metabolism related genes in EpiWAT from obese mice with or without LY3437943 treatment. Sample sizes for each gene were as follows (HFD-vehicle/HFD-LY3437943): *Kat1, n* = 5/5; *Kat2*, *n* = 4/4; *Kat3*, *n* = 5/5; *Kat4*, *n* = 5/5; *Kmo, n* = 4/5; and *Kynu, n* = 4/5 biologically independent mice. Data are mean ± SEM.; *p* value was determined by two-tailed unpaired Student’s *t*-test. **O** Relative abundance of Kynurenic acid/Kynurenine ratio in EpiWAT, and IngWAT, subcutaneous adipose tissue from obese mice with or without LY3437943 treatment. *n* = 5 for HFD-vehicle, *n* = 6 for HFD-LY3437943, biologically independent mice. Data are mean ± SEM.; *p* value was determined by two-tailed unpaired Student’s *t*-test.

Mice were fed a HFD for 13 weeks to induce obesity. Subsequently, a 3-week intervention period was initiated where mice received either vehicle or LY3437943, the novel triple GIP, GLP-1, and glucagon receptor agonist, a compound designed to induce weight loss (Fig. 5A)^49^. HFD for 13 weeks significantly increased body weight, and LY3437943 i.p. injection for 3 weeks significantly decrease body weight and food intake even in the HFD group, without affecting echocardiographic parameters (Fig. 5B, C, and Supplementary Table S5). This approach allowed us to examine the effects of both obesity induction and subsequent weight loss on atrial lymphatics and AFib susceptibility. To assess the systemic effects of our interventions, we performed comprehensive metabolic phenotyping. Glucose tolerance tests (GTT) and insulin tolerance tests (ITT) were conducted to evaluate glucose homeostasis and insulin sensitivity (Fig. 5D, E). Notably, the LY3437943-induced weight loss significantly improved these metabolic parameters (Fig. 5D, E). Measurements of various tissue weights, including heart, liver, and adipose depots, demonstrated that the weight loss intervention with LY3437943 effectively reduced body weight and adipose tissue mass (Fig. 5F).

Remarkably, the weight loss intervention led to a significant upregulation of lymphangiogenesis-related genes (Fig. 5G). Immunohistochemical analysis using LYVE1 staining corroborated these molecular findings (Fig. 5H). The weight loss intervention significantly increased the number of LYVE1-positive cells, suggesting a restoration of atrial lymphatic networks (Fig. 5I). Interestingly, unlike in the AngII model, we did not observe significant differences in expression of inflammation- or fibrosis-related genes between groups (Fig. 5G). Furthermore, the weight loss intervention did not affect NLRP3 inflammasome protein levels (Supplementary Fig. S8A–C). To examine the temporal relationship between lymphangiogenesis and inflammatory signaling, we analyzed gene expression at an earlier time point (1 week of LY3437943 treatment) and found that lymphangiogenesis-related genes were already upregulated while NLRP3-related genes remained unchanged (Supplementary Fig. S8D–E). Additionally, in early obesity (4 weeks HFD), lymphangiogenesis-related genes were decreased while NLRP3 markers showed no changes (Supplementary Fig. S8F–I). These temporal analyses suggests that the lymphatic changes by LY3437943-induced weight loss intervention may precede or occur independently of overt inflammatory or fibrotic remodeling. And also, these findings indicate a direct role of lymphangiogenesis in modulating AFib susceptibility. To assess the functional consequences of these obesity-induced changes, we evaluated AFib inducibility using both in vivo and ex vivo protocols, mirroring our approach in the AngII model. Strikingly, the weight loss intervention substantially reduced AFib inducibility and AFib duration (Fig. 5J–L). These findings align closely with our observations in the AngII model, reinforcing the association between impaired lymphangiogenesis and increased AFib susceptibility across different pathological contexts.

The weight loss intervention also led to a significant upregulation of *indoleamine 2,3-dioxygenase 1* (*Ido1*) gene in adipose tissue (Supplementary Fig. S9A). As the rate-limiting enzyme catalyzing the initial step of tryptophan catabolism to N-formylkynurenine in the kynurenine pathway, *Ido1* plays a pivotal role in the generation of downstream metabolites including kynurenic acid^50^. Notably, the upregulation of *Ido1* expression was markedly more pronounced in epididymal white adipose tissue (EpiWAT, visceral adipose tissue), exhibiting an ~three-fold higher induction compared to inguinal white adipose tissue (IngWAT, subcutaneous adipose tissue). This depot-specific differential regulation suggests that EpiWAT may more robustly engage the kynurenic acid pathway in response to LY3437943 treatment^50^. Interestingly, LY3437943 treatment led to a significant downregulation of *kynurenine aminotransferases 1* (*Kat1*) and *kynurenine aminotransferases 2* (*Kat2*) specifically in EpiWAT, visceral adipose tissue, the enzymes responsible for converting kynurenine to kynurenic acid (Fig. 5M, N, Supplementary Fig. S9B, C). In contrast, the expression of other kynurenine pathway enzymes, including *kynurenine 3-monooxygenase* (*Kmo*) and *kynureninase* (*Kynu*), remained unchanged (Fig. 5M, N). Additionally, while *Kat3* and *Kat4* expression was unaffected in EpiWAT, both were significantly upregulated in IngWAT (Supplementary Fig. S9B, C). Notably, these alterations in gene expression were not observed in IngWAT for *Kat1* and *Kat2*, highlighting the depot-specific regulation of the kynurenic acid pathway (Fig. 5M, N, and Supplementary Fig. S9B, C). To validate these transcriptional changes, mass spectrometry analysis revealed that LY3437943 treatment significantly decreased the kynurenic acid/kynurenine ratio specifically in EpiWAT, while the ratio in IngWAT remained unchanged despite *Kat3* and *Kat4* upregulation (Fig. 5O). The selective modulation of kynurenine metabolism in visceral adipose tissue, characterized by increased *Ido1* expression, decreased *Kat1* and *Kat2* expression, and reduced kynurenic acid production, underscores both the tissue-specific importance of the kynurenic acid pathway and its potential role in the pathogenesis of atrial fibrillation. The heightened metabolic response in EpiWAT indicates that the visceral adipose depot, such as EAT in human, may serve as a preferential therapeutic target for LY3437943-mediated metabolic modulation through the kynurenic acid pathway.

## Discussion

Our results propose the following mechanistic model for AFib development: EAT-derived kynurenic acid acts through GPR35 to fundamentally disrupt lymphatic endothelial cell metabolism, characterized by reduced branched-chain amino acid levels and impaired TCA cycle flux. These metabolic perturbations manifest as compromised mitochondrial homeostasis, evidenced by shortened, swollen mitochondria with reduced cristae density. This metabolic dysfunction, in turn, promotes endothelial-to-mesenchymal transition and impairs lymphatic vessel formation, ultimately leading to reduced lymphatic vessel density. The resultant lymphatic dysfunction contributes to structural and electrical remodeling of the atria, increasing susceptibility to AFib. Notably, we demonstrate that this pathogenic cascade can be therapeutically targeted through two distinct approaches: direct promotion of lymphangiogenesis via VEGFC administration and metabolic modulation through weight loss intervention using LY3437943. Both interventions effectively restored lymphatic vessel density and attenuated AFib susceptibility, establishing lymphatic dysfunction as both a crucial pathogenic mechanism and a promising therapeutic target in AFib. This model integrates both cellular metabolism and tissue-level remodeling, providing a comprehensive framework for understanding AFib pathogenesis and identifying therapeutic interventions.

The present work highlighted the critical role of EAT-lymphatic interaction in AFib pathogenesis. Our in vitro studies reveal that EAT-secreted factors, particularly kynurenic acid, directly impair lymphatic endothelial cell function and induce EndMT. This finding adds to the growing body of evidence implicating EAT in AFib pathogenesis^26^ and identifies a novel mechanism by which adipose tissue influences atrial structure and function. The role of kynurenic acid in mediating these effects is particularly intriguing, as this metabolite has been previously implicated in various physiological and pathological processes^41,51^, but its role in lymphatic biology was hitherto unknown. The metabolomic analyses uncovered significant alterations in BCAA and TCA cycle metabolites in LECs exposed to kynurenic acid. These metabolic changes, coupled with the observed mitochondrial abnormalities, suggest that disruption of cellular energy metabolism may be a key mechanism by which kynurenic acid impairs lymphatic function. This finding aligns with growing evidence linking metabolic dysfunction to AFib pathogenesis^52–54^. The identification of GPR35 as the primary receptor mediating kynurenic acid’s effects on lymphatic endothelial cells provides a potential pharmacological target for intervention. Mass spectrometry analysis revealed significantly elevated kynurenic acid concentrations in both AFib-EAT tissue and conditioned media compared to No AFib controls (Supplementary Figs. S10A, B), with levels comparable to physiological blood concentrations reported in the literature^36^. Other kynurenic acid pathway metabolites including tryptophan, kynurenine, 3-hydroxykynurenine, and quinolinic acid remained unchanged (Supplementary Figs. S10C, D). Importantly, AFib-EAT conditioned media upregulated GPR35 expression in LECs, and chronic kynurenic acid exposure further enhanced GPR35 expression, potentially creating a positive feedback loop that amplifies signaling sensitivity (Supplementary Fig. S5B, C). While the measured kynurenic acid concentrations appear lower than reported EC50 values for GPR35 activation, the combination of prolonged exposure and enhanced receptor expression may have cumulative effects on GPR35 signaling. Notably, CXCL17, another endogenous GPR35 ligand, showed no differences between AFib and No AFib groups (Supplementary Fig. S10E, F). GPR35 has been implicated in various cardiovascular pathologies^55–58^, and our study extends its role to lymphatic dysfunction in AFib. The dual mechanism of elevated kynurenic acid levels and enhanced GPR35 expression in AFib conditions suggests that therapeutic interventions targeting this pathway may be particularly effective. Evaluation of the therapeutic potential of GPR35 antagonists in preventing or reversing AFib-related lymphatic impairment awaits future investigation.

Our in vivo studies demonstrate that promoting lymphangiogenesis through VEGFC administration or weight loss can attenuate AFib susceptibility in both AngII-induced and obesity-related AFib models. These findings suggest that targeting lymphangiogenesis may represent a promising therapeutic strategy for AFib prevention and treatment. Moreover, the observation that lymphangiogenesis-mediated protection occurs independently of changes in inflammatory or fibrotic gene expression highlights the unique contribution of lymphatic function to atrial health.

In conclusion, our findings reveal a novel pathogenic mechanism in AFib involving EAT-mediated impairment of atrial lymphangiogenesis. This work not only advances our understanding of AFib pathophysiology but also identifies potential therapeutic targets.

### Limitations of the work

Several limitations should be acknowledged in this study. First, both AFib groups were dominated by valve disease patients, whereas controls consisted mostly of coronary artery disease (CAD) patients, raising the possibility that underlying valve disease may have influenced the observed lymphangiogenesis changes. The timing from last documented paroxysmal AFib episodes to sample collection could potentially affect tissue characteristics, but accurate determination was challenging due to the frequent asymptomatic nature of episodes. PerAF patients also had lower estimated glomerular filtration rate (eGFR) and larger LA size, which may confound lymphangiogenesis changes through inflammatory pathway activation. Our small sample size precluded reliable assessment of interaction effects. Second, while we cannot completely exclude the influence of ventricular dysfunction on the observed atrial phenotype in our mouse models, temporal analyses at early time points demonstrate that alterations in lymphangiogenesis consistently precede ventricular dysfunction (Supplementary Fig. S11A-F, and Supplementary Table S6, S7), supporting the concept that impaired lymphangiogenesis is a primary mechanism rather than a downstream consequence of diastolic dysfunction in AngII- and obesity-induced AFib pathophysiology. Third, while measured kynurenic acid concentrations were comparable to physiological blood levels, they appeared lower than reported GPR35 EC50 values. Our findings suggest prolonged exposure and enhanced receptor expression may have cumulative signaling effects, but determining the precise mechanisms is of paramount importance for future investigation. Besides, kynurenic acid accumulation mechanisms may differ between tissue types and species, warranting further study. Notably, however, kynurenic acid concentrations were relatively consistent between human and mouse adipose tissue: human EAT showed median 5.35 (IQR 4.72–6.49) ng/g tissue, while mouse visceral adipose tissue showed comparable concentrations across models (AngII model EpiWAT: median 7.13, IQR 4.91–15.08 ng/g tissue; HFD model EpiWAT: median 11.04, IQR 8.58–13.35 ng/g tissue), supporting the translational relevance of our mouse findings to human pathophysiology. Fourth, the adipocyte differentiation protocol employed supraphysiological concentrations of insulin and PPARγ agonist that may not fully recapitulate physiological conditions, potentially affecting cellular metabolic profiles.

## Methods

### Human subjects

The study protocol was approved by the Ethics Committee of Oita University Hospital (approval number: 794 and 1797) and was conducted in accordance with the tenets of the Declaration of Helsinki. This study was registered in the University Hospital Medical Information Network Clinical Trials Registry (UMIN000042229). Left atrial appendage (LAA), subcutaneous adipose tissue (SAT) and epicardial adipose tissue (EAT) were collected under the Oita University Committee on Clinical Investigations. Potential subjects were recruited randomly from the operating room rosters of Oita University Hospital and Oita Oka Hospital. Subjects undergoing elective open-heart surgery were included and provided written informed consent preoperatively. Human SAT and EAT segments were obtained from the surgical site and the atrioventricular/interventricular groove of the heart during surgery. The tissues were placed in ice-cold Krebs-HEPES buffer (NaCl 99 mmol/L, KCl 4.7 mmol/L, MgSO4 1.2 mmol/L, KH2PO4 1 mmol/L, CaCl2 1.9 mmol/L, NaHCO3 25 mmol/L, glucose 25 mmol/L, HEPES 20 mmol/L; pH 7.35) and immediately transferred to the laboratory.

### Extraction of stromal vascular fraction and cell culture

Fresh EAT from the patients with or without AFib was minced and digested with collagenase II solution (2 mg/mL) for 15 min at 37 °C to degrade fibrous tissue. The digested samples were centrifuged at 300 × *g* for 5 min and resuspended in Dulbecco’s Modified Eagle Medium Nutrient Mixture F-12 (Catalog no. 11320-033, Gibco/Thermo Fisher Scientific) supplemented with 10% fetal bovine serum and 0.2 ng/mL human epidermal growth factor (Thermo Fisher Scientific). When the isolated cells reached 100% confluence, the medium was replaced with a preadipocyte differentiation medium (Catalog no. C-27437, PromoCell) and cultured for 5 days. They were then cultured in an adipocyte nutrition medium (Catalog no. C-27439, PromoCell) for 14 days, which successfully resulted in mature/differentiated adipocytes as described previously^33,34^. After the maturation, cell culture medium was switched to M199 culture medium (Gibco, Invitrogen) for 24 hours. Then, cell culture supernatant was collected and stored at −80 °C.

### Animals

All animal experiments were approved by the Oita University Animal Ethics Committee, Japan (approval no. 223201, 243201, and 243204), for the care and use of laboratory animals, which follow the guidelines established by the US National Institutes of Health. C57BL/6J wild-type mice and Sprague-Dawley rats were obtained from KBT Oriental at 8 weeks of age and were kept in an animal room, one animal in each cage, with a chow and water at ambient temperature (22°C) and controlled humidity under a 12 hr:12 hr light-dark cycle. For isolation of the heart, mice and rats were euthanized by an intraperitoneal injection of sodium pentobarbital with a dosage of 150 mg/kg.

### Organo-culture

Hearts from eight-week-old male Sprague-Dawley rats were rapidly excised under anesthesia. Isolated left atrial tissue samples were transferred into cold CO_2_-independent medium. Inserts containing polyester porous membranes (pore size, 0.4 µm; Millicell, Merck Millipore) were filled with 2 mL of M199 culture medium containing insulin, transferrin, and sodium selenite (ITS, 1/1000, Sigma-Aldrich, St. Louis, MO, USA), 5% FBS, 10 mM glucose, 1 nM isoproterenol, and 100 U/mL of penicillin/streptomycin. Isolated atria were placed on the porous membranes with the endothelial side facing toward the membrane. Then, a drop of culture medium was placed onto the atria, which were removed and incubated for 15 min at 37 °C (5% CO2). After preincubation, 20 µL of the collected cell culture supernatant or vehicle medium (this medium consisted entirely of M199 culture medium) was dropped onto the epicardial side of the atria once a day and incubated for 7 days (37 °C, 5% CO2). For the administration of angiotensin II, 20 µL of M199 culture medium with angiotensin II (100 nM) was dropped onto the epicardial side of the atrium once a day and incubated for 14 days.

### Cell culture

Human lymphatic endothelial cells (Promo cell, Catalog no. C-12217) were purchased and used to validate the effects of EAT-secreted factors and kynurenic acid on human lymphatic endothelial cells (LECs). Human atrial cardiac fibroblasts (Lonza, Catalog no. CC-2903) were also used to validate the direct influences of kynurenic acid on fibroblasts. Neonatal rat cardiomyocytes were prepared from Sprague–Dawley rats and cultured as previously described^59^, and used to validate the direct influences of kynurenic acid on cardiomyocytes. LECs, cardiac fibroblasts, and cardiomyocytes were cultured using Endothelial Cell Growth medium MV2 (Promo Cell, Catalog no. C-22022) or Cardiac Fibroblast Growth Medium-3 (Lonza, Catalog no. CC-4526) or Dulbecco’s modified Eagle’s medium (DMEM). After reaching 100% confluence, the cell culture supernatant from mature/differentiated adipocytes was administered to the Endothelial Cell Growth medium at a 1:4 dilution for 4 days. For the kynurenic acid administration, 100% confluent LECs, cardiac fibroblasts, and cardiomyocytes were cultured with the Endothelial Cell Growth medium or Cardiac Fibroblast Growth Medium-3 or DMEM containing kynurenic acid (0 nM, 5 nM, 50 nM or 500 nM) for 4 days. ML145 (50 nM) and CID2745687 (50 nM) were added with kynurenic acid to inhibit GPR35. To assess signaling pathways involved in endothelial-to-mesenchymal transition, LECs were cultured in Endothelial Cell Growth medium under the following conditions: control (vehicle only), kynurenic acid (50 nM) alone, kynurenic acid plus SB431542 (100 nM; Catalog No. S1067, Selleck), and kynurenic acid plus Takinib (20 nM; Catalog No. S8663, Selleck). Cells were incubated for 24 hours. To investigate the effects of branched chain amino acid (BCAA) deprivation in LECs, cells were cultured in either complete medium or BCAA-free medium for 12 or 24 hours. The BCAA-free medium was prepared by supplementing DMEM (Catalog No. 048-33575, FUJIFILM Wako) with the following amino acids: L-Arginine HCl (84 mg/L), L-Cystine (48.34 mg/L), L-Glutamine (584 mg/L), Glycine (30 mg/L), L-Histidine HCl·H₂O (42 mg/L), L-Lysine HCl (146 mg/L), L-Methionine (30 mg/L), L-Phenylalanine (66 mg/L), L-Serine (42 mg/L), L-Threonine (95 mg/L), L-Tryptophan (16 mg/L), and L-Tyrosine (71.59 mg/L). The complete medium was prepared by additionally supplementing the above formulation with L-Isoleucine (105 mg/L), L-Leucine (105 mg/L), and L-Valine (94 mg/L).

### Tube formation assay

A 12-well plate was pre-coated with Matrigel (BD Biosciences, San Jose, USA) (0.4 mL/well). After polymerization of Matrigel at 37˚C for 1 h, LECs were seeded in each well at a density of 1.75×10^5^ cells/400 μL/well. After 3 h of incubation in Endothelial Cell Growth medium MV2 medium containing the cell culture supernatant from mature/differentiated adipocytes or kynurenic acid (0 nM, or 50 nM), tube-like structures were photographed under a microscopy (BZ-9000, KEYENCE) with 10× objective lenses.

### Dextran assay for measurement of cell permeability

LECs were cultured on 24-well plate with 3.0 μm pore size transwell inserts (Fisher Scientific, Corning Inc., Catalog no. 353096) for 7 days (to confluence). After the confluence, the medium was replaced with an Endothelial Cell Growth medium MV2 medium containing the cell culture supernatant from mature/differentiated adipocytes or kynurenic acid (0 nM, 5 nM, 50 nM or 500 nM), and cultured for 4 days. Each insert was washed once with 1x HBSS, and transferred to fresh 24-well plate, then, 200 μL of HBSS containing 1 μg/mL FITC-Dextran was added to the upper chamber and 600 μL of HBSS to the lower chamber. The 24-well plate with transwell inserts was incubated for 1 hour at 37˚C in 5% CO2 with gentle shaking. The concentration of FITC-Dextran transferred to the lower chamber was determined using the Microplate Reader (Biotek, Catalog no. 258632) with excitation and emission wavelengths of 492 nm and 520 nm, respectively.

### Oxygen consumption assays

Cellular OCR was measured using the Seahorse XFe Extracellular Flux Analyzer (Agilent) as previously described^60^. Cells were seeded at 10,000 cells per well in a Seahorse XFp Cell Culture plate. For kynurenic acid experiments, cells were stimulated with 50 nM or 500 nM kynurenic acid for 48 hrs. For stimulation with human adipose supernatant, cells were cultured in the supernatant that was suspended 1:3 with culture medium for 48 hrs. For the measurement of uncoupled respiration, cells were treated with 2.5 μM oligomycin, followed by carbonyl cyanide 4-(trifluoromethoxy) phenylhydrazone (FCCP, 2 μM), and antimycin A (0.5 μM).

### 3D spheroid invasion assay

A total of 3000 LECs were resuspended in 1× Spheroid Formation ECM (Trevigen) and seeded into a 3D Culture Qualified 96 Well Spheroid Formation Plate. After spheroid formation, Invasion Matrix (Trevigen) was added to each well. Following incubation at 37 °C for 1 h to allow gel formation, Endothelial Cell Growth medium MV2 with No AFib or AFib-EAT conditioned media, and with vehicle or kynurenic acid was added. Spheroids were cultured for 6 days at 37 °C and imaged using a Keyence BZ-X700 microscope. Invasive sprouting was quantified using ImageJ software (NIH) by measuring changes in the total area.

### Cell migration assay

Cell migration was assessed using the CytoSelect™ 96-Well Cell Migration Assay Kit (Cell Biolabs). LECs were resuspended at 0.1 × 10⁶ cells/ml in serum-free medium and seeded into the migration plate. Chemoattractants included Endothelial Cell Growth medium MV2 with No AFib or AFib-EAT conditioned media, with vehicle or kynurenic acid. After incubation, migrated cells were lysed with 4× Lysis Buffer containing CyQuant® GR Dye, and fluorescence was measured at 480 nm/520 nm. Migration was quantified as relative fluorescence units (RFU).

### Cell proliferation assays

LECs were treated with No AFib or AFib-EAT conditioned media, with vehicle or kynurenic acid for 2 days, then incubated with 10 μM EdU (Click-iT Plus EdU Flow Cytometry Assay Kit, Thermo Fisher Scientific) for 2 h in serum-free medium. Subsequently, cells were fixed and processed according to the manufacturer’s instructions. Cell proliferation (%) was calculated as the frequency of EdU-positive cells. All cells were analyzed using a FACS Melody and FlowJo software (version 10.8.1) was used for data analysis.

### Mitochondrial membrane potential and superoxide detection

Mitochondrial membrane potential and superoxide generation were assessed simultaneously using JC-1 MitoMP Detection Kit (Dojindo, MT09) and MitoBright ROS Deep Red (Dojindo, MT16). LECs were treated with No AFib or AFib-EAT conditioned media, with vehicle or kynurenic acid for 2 days. Cells were incubated with JC-1 working solution (4 μmol/L) for 30 min at 37 °C. Then, cells were incubated with MitoBright ROS Deep Red working solution (10 μmol/L) for 30 min at 37 °C. Cells were resuspended in Imaging Buffer solution and fluorescence was measured using a microplate reader. Mitochondrial membrane potential was calculated as the red/green fluorescence ratio.

### LC-TOFMS analysis

Cultured medium from mature/differentiated adipocytes was centrifuged to remove debris. Then, a 1000 µL sample of the collected supernatant was evaporated to dryness, and the dried residue was reconstituted in 200 µL of Milli-Q water. Subsequently, 100 µL of the reconstituted solution was mixed with 300 µL of methanol containing internal standards (H3304-1002, Human Metabolome Technologies (HMT), Tsuruoka, Yamagata, Japan). The mixture was centrifuged at 2300 × *g*, 4 °C for 5 min. The supernatant was then evaporated to dryness under nitrogen and reconstituted in 100 µL of 50% isopropanol (v/v) for metabolome analysis. Metabolome analysis was conducted by using liquid chromatography time-of-flight mass spectrometry (LC-TOFMS) based on the methods described previously^61^. Briefly, LC-TOFMS analysis was carried out by an Agilent 1260 HPLC system with an Agilent 6230 time-of-flight mass spectrometer (Agilent Technologies, Inc., Santa Clara, CA, USA). The systems were controlled by Agilent MassHunter Workstation Data Acquisition (Agilent Technologies) and connected by an ODS column (2 mm i.d. × 50 mm, 2 μm). The spectrometer was scanned from m/z 100 to 1,700 and peaks were extracted using MasterHands, automatic integration software (Keio University, Tsuruoka, Yamagata, Japan) in order to obtain peak information including m/z, peak area, and retention time (RT). Signal peaks corresponding to isotopomers, adduct ions, and other product ions of known metabolites were excluded, and the remaining peaks were annotated according to metabolite database based on their m/z values and RTs. Areas of the annotated peaks were then normalized to internal standards and sample amount in order to obtain relative levels of each metabolite.

### GC/MS analysis

The GC-MS/MS analysis was performed on a GCMS-TQ8040 system (Shimadzu Corporation, Kyoto, Japan) equipped with a DB-5 capillary column (30 m × 0.25 mm inner diameter, film thickness 1 µm; Agilent, Santa Clara, CA, USA). Each 1 µm aliquot of the derivatized sample solution was automatically injected in splitless mode into the gas-liquid chromatography column using an auto-injector (AOC-20i; Shimadzu Corporation). During the GCMS-TQ8040 analysis, the injector temperature was kept at 280 °C, and helium was used as a carrier gas at a constant flow rate of 39.0 cm/s. The GC column temperature was programmed to remain at 100 °C for 4 min and then to rise to 320 °C at a rate of 10 °C/min, holding at 320 °C for a further 11 min. The ionization voltage was 70 eV. Argon was used for collision-induced dissociation. Metabolite detection was performed using the Smart Metabolites Database Ver. 3 software program (Shimadzu Corporation) using the method described in a previous study with some modifications^62^. The 2-isopropylmalic acid contained in the extraction solution was also used to evaluate the stability of our GC-MS/MS analysis system. Peak identification was performed automatically and then confirmed manually based on the specific precursor and product ions as well as the retention time.

### LC-MS/MS analysis

Adipose tissue samples were homogenized in LC/MS-grade methanol (50 µL per 10 mg tissue) and centrifuged at 20,000 × g, 4 °C for 10 min. Supernatants were collected and used for analysis. Cultured medium from mature/differentiated adipocytes was centrifuged to remove debris. Then, a 1000 µL sample of the collected supernatant was evaporated to dryness, and the dried residue was reconstituted in 200 µL of Milli-Q water. Subsequently, 100 µL of the reconstituted solution was mixed with 300 µL of methanol. The mixture was centrifuged at 2300 × g, 4 °C for 5 min. The supernatant was then evaporated to dryness under nitrogen and reconstituted in 100 µL of 50% isopropanol (v/v) for analysis. LC-MS/MS analysis was performed using a Shimadzu LCMS-8040 system with a Discovery HS5-3 column (2.1 mm × 150 mm, 3 µm; Merck). Mobile phases consisted of 0.1% formic acid in water (A) and acetonitrile (B) with gradient elution at 0.25 mL/min flow rate. Mass spectrometric detection was performed using electrospray ionization in positive ion mode. Concentrations were calculated using standard curves.

### Bioinformatics for single cell RNA-seq data

Single-cell RNA sequencing data analysis was performed by using published datasets in human left atrial tissue from No AFib and persistent AFib patients, and in mice left atrial tissue from No AFib and AFib models^29^. The barcode/Unique Molecular Identifier (UMI) data matrix were obtained from GEO website. The UMI counts were loaded into R (v4.4.1) with Seurat package (v5.1.0)^63^. We performed quality check and filtered out low quality cells. The counts data was normalized with LogNormalize function of Seurat and all samples were integrated with CCA method and clustered with PCA and UMAP algorithm.

### AngiotensinII and VEGFC administration

Eight-weeks-old C57BL/6J wild-type mice were randomly assigned to infusion of either AngiotensinII (AngII) or vehicle (VEH). After anaesthetized by an intraperitoneal injection of sodium pentobarbital with a dosage of 150 mg/kg, an Alzet 1002 or 2004 mini-pump was implanted subcutaneously for continuous infusion of AngII (2.0 mg/kg/day) or an equal volume of vehicle (1%PBS) for 2 or 4 weeks. Recombinant Human VEGFC (Cys156Ser) Protein (50 μg/kg/day, R&D system) was also administrated by the Alzet mini-pump for 2 or 4 weeks. Blood Pressure and heart rate were measured by the tail-cuff (Softron, Tokyo, Japan) method at 2 weeks and 4 weeks. Transthoracic Echocardiography was performed to evaluate cardiac structure and function, including both systolic and diastolic function, using a high-resolution ultrasound imaging system (Vevo 3100, VisualSonics, Fujifilm, Toronto, Canada). Mice were anesthetized with inhalation of isoflurane (2% for induction and 1.0–1.5% for maintenance) in 100% oxygen at a flow rate of 0.8–1.2 L/min and placed on a temperature-controlled platform to maintain body temperature. Echocardiographic analysis was performed in a blinded manner. Wet heart weights were measured immediately after euthanasia. Then hearts were dried using a Concentrator plus (Eppendorf) since the weight became constant. Dry weights were measured after desiccation. The wet-to-dry weight ratio was calculated by dividing the wet weight over the dry weight for each animal. The percentage of water content was calculated by the formula of (wet heart weight - dry heart weight)/wet heart weight.

### Treatment with high fat diet and Retatrutide, LY3437943

Eight-weeks-old C57BL/6J wild-type mice were randomly assigned to be fed by normal-fat diet (NFD: 20% fat, 56% carbohydrate, 24% protein; Diet Research) or HFD (60% fat, 20% carbohydrate, 20% protein; Diet Research) for 16 weeks. At 13 weeks, treatment with a retatrutide, LY3437943 (MCE) at 10 nmol/kg body weight or vehicle, was started and injected intraperitoneally every third day for 3 weeks. Body weight and food intake were measured every week. For glucose tolerance tests, mice fasted for 6 h from 9:00 to 15:00, were administered glucose intraperitoneally (1.5 g kg^−1^ body weight for the NFD group, and 1.0 g kg^−1^ body weight for the HFD group). For insulin tolerance tests, mice on a HFD fasted for 3 h from 9:00 to 12:00, were injected intraperitoneally with insulin (1 U kg^−1^ body weight). Blood samples were collected before injection, and glucose levels were measured using blood glucose test strips (SUGL1, ForaCare Japan). Transthoracic echocardiography was performed as described above.

### qRT-PCR

Total RNA was extracted from tissues and cells using a spin column-based RNA purification method with a QIA shredder and RNeasy Mini Kit (QIAGEN). The RNA concentration and integrity were assessed spectrophotometrically using the NanoDrop 2000 spectrophotometer. RNA was reverse transcribed to complementary deoxyribonucleic acid (cDNA) using SuperScript VILO Master Mix (Thermo Fisher Scientific) with the ezDNaseTM Enzyme Kit (Invitrogen/Thermo Fisher Scientific) according to the manufacturer’s protocols. qPCR was performed with TaqMan chemistry using the standard universal TaqMan protocol according to the manufacturer’s instructions using the LightCycler 96 (Roche Diagnostics). All samples were duplicated using 5 ng of cDNA as the starting mass. The relative mRNA expression in each sample was normalized to the GAPDH by determining by the ΔΔ Ct method and normalized to an internal calibrator specific to each gene using the formula 2^−ΔΔCT^. The IDs of the TaqMan probes (Thermo Fisher Scientific) used are *GAPDH*: Hs02786624_g1, *Gapdh*: Rn 01775763_g1, *Gapdh*: Mm99999915_g1, *LYVE1*: Hs00272659_m1, *Lyve1*: Rn01510421_m1, *Lyve1*: Mm00475056_m1, *PROX1*: Hs00896293_m1, *Prox1:* Rn02103824_s1, *Prox*1: Mm00435969_m1, *VEGFC*: Hs01099203_m1, *Vegfc*: Rn01488076_m1, *Vegfc*: Mm00437310_m1, *VEGFR3*: Hs01047677_m1, *Vegfr3*: Rn00677893_m1, *Vegfr3*: Mm01292604_m1, *VEGFA*: Hs00900055_m1, *VEGFB*: Hs00173634_m1, *VEGFR1*: Hs01052961_m1, *VEGFR2*: Hs00911700_m1, *CD68*: Hs02836816_g1, *CD11c*: Hs00174217_m1, *FABP4*: Hs01086177_m1, *CEBPA*: Hs00269972_s1, *PPARG*: Hs01115513_m1, *PLIN1*: Hs00160173_m1, *TGFb1*: Hs00998133_m1,*Tgfb1*: Rn00572010_m1, *Tgfb1*: Mm01178820_m1, *TGFb2*: Hs00234244_m1, *CTGF*: Hs00170014_m1, *ACTA2*: Hs00426835_g1, *Acta2*: Rn01759928_g1, COL1A1: Hs00164004_m1, *Col1a1*: Rn01463848_m1, *Col1a1*: Mm00801666_g1, *COL3A1*: Hs00943809_m1, *Col3a1*: Rn01437681_m1, *Col3a1*: Mm00802300_m1, *Ccl2*: Rn00580555_m1, *Ccl2*: Mm00441242_m1, *Il1b*: Rn00580432_m1, *Tnf*: Rn99999017_m1, *TAGLN*: Hs01038777_g1, *CYTB*: Hs02596867_s1, *COX1*: Hs00377726_m1, *COX2*: Hs00153133_m1, *COX3*: Hs02596866_g1, *ND2*: Hs02596874_g1, *ND4*: Hs02596876_g1, *ATP6*: Hs02596862_g1, *ATP8*: Hs02596863_g1, *Il6*: Mm00446190_m1, *F4/80*: Mm00802529_m1, *Nppa*: Mm01255747_g1, *Nppb*: Mm01255770_g1, *Myh7*: Mm00600555_m1, *Ido1*: Mm00492590_m1, *Kat1*: Mm00549584_m1, *Kat2*: Mm00496169_m1, *Kat3*: Mm00620553_m1, *Kat4*: Mm00494703_m1, *Kmo*: Mm01321343_m1, *Kynu*: Mm00551012_m1, *Nlrp3*: Mm00840904_m1, *Asc*: Mm00445747_g1, *Casp1*: Mm00438023_m1, *Il1b*: Mm00434228_m1, *GPR35*: Hs00271114_s1, and *Gpr35*: Mm01973686_s1.

### Tissue histology and immunohistochemistry

Isolated heart tissue was fixed in 4% paraformaldehyde, embedded in paraffin, cut into 5 μm serial sections, mounted, and processed for tissue histology and immunochemical staining. Masson’s trichrome staining was used to assess interstitial fibrosis. Images were captured using a BZ-9000 Biolevo epifluorescence microscope (Keyence) with an attached digital camera. Myocardial tissues were evaluated at 400× magnification using Keyence software. The percentage of fibrosis and of intercellular space was measured as previously described. Briefly, to validate the visually assessed extent of fibrosis and intercellular space, 3-5 images were analyzed at 400× magnification in each individual per section were analyzed to obtain mean values. The percentage of fibrosis and of intercellular space were determined by calculating the ratio of areas of fibrosis or intercellular space to total tissue area. For immunostaining, paraffin-embedded tissues were deparaffinized three times in xylene and subsequently rehydrated. After heat-induced epitope retrieval using target retrieval solution (Dako), the tissues were blocked in PBS containing 10% goat serum with 0.1% Tween 20 for 60 min. After washing in PBS, slides were incubated with goat anti LYVE-1 antibody (3 µg/mL Catalog no. AF2089 R&D Systems) and rabbit anti GPR35 antibody (1:100 Catalog no. 55248-1-AP Proteintech) for the tissues from humans, rabbit anti LYVE-1 antibody (1:100 Catalog no. PA1-16635 Invitrogen, Carlsbad, California), goat anti PROX1 antibody (5 µg/mL Catalog no. AF2727 R&D Systems), rabbit anti TAGLN antibody (1:100 Catalog no. #40471 Cell Signaling Technology) and rat anti F4/80 antibody (1:100 Catalog no. ab16911 Abcam) for mouse and rat tissues overnight at 4 °C, followed by incubation with Alexa Fluor 488 antibody (1:1000, Thermo Fisher), Alexa Fluor 594 antibody (1:1000, Thermo Fisher), or bright conjugates of wheat germ agglutinin (WGA). After washing, the sections were stained with DAPI for nuclei. Images of tissue samples were captured using a BZ-9000 Biolevo epifluorescence microscope (Keyence) with an attached digital camera. The LEC density and LYVE1 positive area were evaluated in 3-5 randomly selected fields at 100× magnification for human samples and at 400× magnification for mice and rats sample. The proportion of LYVE1 and GPR35 double-positive area was evaluated at 200× magnification and calculated as the number of LYVE1 and GPR35 double-positive area divided by the total number of LYVE1-positive area. PROX1 positive cells and TAGLN positive cells were counted in 3-5 randomly selected fields at 400× magnification for mice. Blinded automated image analysis was performed with ImageJ software (version 1.53a, National Institutes of Health), by pre-defined homogeneous thresholding criteria and the “analyze particles” function, as previously described^59^.

### Immunocytochemistry

LECs were fixed in 4% paraformaldehyde, embedded in paraffin, mounted, and processed for immunochemical staining paraffin-embedded cells were deparaffinized three times in xylene and subsequently rehydrated. After heat-induced epitope retrieval using target retrieval solution (Dako) and permeabilization using 0.2% Triton X-100, the cells were blocked in PBS containing 10% goat serum with 0.1% Tween 20 for 60 min. After washing in PBS, slides were incubated with goat PROX1 (10 µg/mL Catalog no. AF2727 R&D Systems) and rabbit TAGLN (1:100 Catalog no. #40471 Cell Signaling Technology) overnight at 4 °C, followed by incubation with Alexa Fluor 488 antibody (1:1000, Thermo Fisher), Alexa Fluor 594 antibody (1:1000, Thermo Fisher). After washing, the cells were stained with DAPI for nuclei. Images of samples were captured using a BZ-9000 Biolevo epifluorescence microscope (Keyence) with an attached digital camera. The mean fluorescence intensity of each sample was evaluated in 3-5 randomly selected fields at 200× magnification using ImageJ software (version 1.53a). To eliminate nonspecific signal and account for background autofluorescence, background fluorescence intensity was subtracted from each measurement.

### Oil Red O staining

Differentiated SVF were rinsed with PBS and fixed with 4% paraformaldehyde. Staining was performed with Oil Red O (Fujifilm) stock solution (0.3% Oil Red O in isopropanol) for 20 min. After washing with water, images were obtained using an inverted phase-contrast microscope. Then, the cells were harvested with 60% isopropanol and centrifuged to measure the Oil Red O dye eluted in the solvent using a spectrophotometer with an absorbance of 520 nm.

### DHE staining

Frozen sections of mouse atrial tissue were incubated with dihydroethidium (DHE; 10 μM Catalog no. D7008 Sigma-Aldrich) at 37 °C for 30 min in the dark. After washing, sections were mounted and images of tissue samples were captured using a BZ-9000 Biolevo epifluorescence microscope (Keyence). The mean fluorescence intensity of each section was evaluated in 3–5 randomly selected fields at 200× magnification using ImageJ software (version 1.53a). To eliminate nonspecific signal and account for background autofluorescence, background fluorescence intensity was subtracted from each measurement.

### Immunoblotting

Tissues and cells were homogenized and lysed in sample buffer solution (0.125 mol/l Tris-HCl, 4 w/v% SDS, 20 w/v% Glycerol, 0.002 w/v% BPB, 10 vol% 2-Mercaptoethanol pH6.8). Total protein lysates were boiled and loaded on SDS-PAGE. Subsequently, separated proteins were transferred onto PVDF membranes. PVDF membrane blots were blocked in 3% BSA/PBS for 30 min at room temperature and incubated overnight at 4 °C with a primary antibody. The primary antibody used were rabbit β-actin (1:1000 Catalog no. #4967 Cell Signaling Technology), rabbit NLRP3 (1:250 Catalog no. NBP2-12446 Novas Biologicals), mouse ASC (1:250 Catalog no. SC-514414 Santa Cruz), mouse Caspase1 (1:500 Catalog no. SC-56036 Santa Cruz), rabbit IL-1β (1:500 Catalog no. ab9722 Abcam), rabbit Malondialdehyde (1:500 Catalog no. ab27642 Abcam), rabbit oxidized-CaMKII (Met281/282) (1:1000 Catalog no. 07-1387 Sigma-Aldrich), rabbit CaMKII (1:1000 Catalog no. ab52476 Abcam), rabbit p-RyR2 (Ser2814) (1:250 Catalog no. A010-31AP Badrilla), rabbit RyR2 (1:500 Catalog no. PA5-104444 Thermo Fisher), goat PROX1 (1:250 Catalog no. AF2727 R&D Systems), rabbit α/β-Tubulin (1:1000 Catalog no. #2148 Cell Signaling Technology), rabbit TAGLN (1:500 Catalog no. #40471 Cell Signaling Technology), rabbit p-SMAD2 (Ser465/467)/SMAD3 (Ser423/425) (1:500 Catalog no. #8828 Cell Signaling Technology), rabbit SMAD2/3 (1:1000 Catalog no. #8685 Cell Signaling Technology), mouse SMAD4 (1:200 Catalog no. SC-7966 Santa Cruz), rabbit p-TAK1 (Thr184/187) (1:500 Catalog no. #4508 Cell Signaling Technology), rabbit TAK1 (1:500 Catalog no. #5206 Cell Signaling Technology), rabbit p-Erk1/2 (1:500 Catalog no. #9101 Cell Signaling Technology), rabbit Erk1/2 (1:1000 Catalog no. #4695 Cell Signaling Technology), rabbit p-p38MAPK (1:1000 Catalog no. #4511 Cell Signaling Technology), and rabbit p38MAPK (1:1000 Catalog no. #8690 Cell Signaling Technology). Then, PVDF membrane blots were washed and incubated for 60 min at room temperature. Anti-rabbit IgG (1:2000 Catalog no. NA9340 Cytiva), Anti-mouse IgG (1:2000 Catalog no. NA9310 Cytiva), and Anti-goat IgG (1:2000 Catalog no. AF109 R&D Systems) were used as a second antibody. Protein bands were visualized using ECLTM Prime (GE Healthcare), and arbitrary optical densities were measured using ImageJ software (version 1.53a).

### Enzyme-linked immunosorbent assay (ELISA)

The protein levels of CXCL17 and the concentrations of quinolinic acid in adipose tissues and EAT-conditioned media were measured with Human CXCL17 ELISA kit (Catalog no. ELH-CXCL17-1, RayBiotech), and Quinolinic Acid ELISA kit (Catalog no. IS I-0100R, ImmuSmol SAS) in accordance with the manufacturers’ instructions.

### Hydroxyproline assay

Collagen content in human LAA was evaluated by quantification of hydroxyproline using a hydroxyproline assay kit (BioVision, Mountain View) according to the manufacturer’s protocol.

### Electron microscopy

Left atrial tissues and LECs were examined by transmission electron microscopy (TEM). Specimens were fixed in a mixture of 2.0% paraformaldehyde and 2.5% glutaraldehyde. They were then postfixed in 2% osmium tetroxide for 2 h at 4 °C, dehydrated in a graded series of ethanol solutions, and embedded in epoxy resin. Semi-thin sections (1.0-µm thick) were stained with 1% toluidine blue for light microscopy. Then, ultrathin sections (80 nm thick) were stained with uranyl acetate and lead citrate and examined with a transmission electron microscope (JEM-1200EXII, JEOL, Tokyo, Japan). The form factor F was used to assess the complexity of mitochondrial shape in LECs, following the method described previously^39,40^. The form factor, calculated as (perimeter^2^/4π area), yields a higher value for more complex shapes and a lower value for simpler, circular shapes, independent of size. Ten mitochondria in every five cells in each group were observed for analysis, and the average values of form factor F were obtained and used for comparison. The perimeter and area of each mitochondrion were measured by manually tracing the outer membranes of the mitochondria using ImageJ software (version 1.53a).

### Cell isolation and sorting

Lymphatic endothelial cells were isolated from mouse atria using MACS and FACS methods. Mouse atria were digested by collagenase D (1.5 U ml^−1^) and dispase II (2.5 U ml^−1^) according to the procedure^64^. MACS CD45 Micro beads for mice (1:10, Catalog no. 130-052-301, Miltenyi Biotec) and MACS LS columns (Miltenyi Biotec) were used to deplete lineage^+^ (Lin^+^) cells. The following antibodies were used for the isolation of mouse atrial lymphatic endothelial cells (Lin^-^: CD31^+^: Podoplanin^+^): Podoplanin-PE (1:80, Catalog no.127407, Biolegend) and CD31-APC (1:300, Catalog no.160209, Biolegend) in autoMACS Rising Solution (Miltenyi Biotec) containing 0.5% BSA in the dark at 4°C for 15 minutes. Cell population (%) was calculated as the frequency of parent. All the cells were isolated and analyzed using a FACS Aria II equipped with 100 mm nozzle diameter and CytoFLEX. FlowJo software (version 10.8.1) was used for data analysis.

### Transesophageal Burst Pacing In Vivo

The mice were anaesthetized by an intraperitoneal injection of sodium pentobarbital at a dosage of 150 mg/kg, and their body temperature was maintained at 37 °C. A 4-French catheter electrode (Japan Lifeline, Tokyo, Japan) was placed at the esophageal position dorsal to the LA. Surface ECG was recorded simultaneously with electrodes in a lead-II configuration. Inducibility of AFib was tested by applying 3 trains of a 1-second burst pacing with the automated stimulator. The first 1-second burst had a cycle length of 30 ms (pulse duration=5 ms). After 3 minutes of stabilization, a second 1-second burst with a cycle length of 20 ms (pulse duration=10 ms) was applied. After 3 minutes of stabilization, the last 1-second burst was applied with a cycle length of 10 ms and a pulse duration of 15 ms. AFib was defined as a rapid irregular atrial rhythm with irregular R–R intervals lasting at least 1 second. The duration of AFib was measured from the end of burst pacing to the first P wave detected after the rapid irregular atrial rhythm.

### Ex vivo electrophysiological studies

Electrophysiological studies in isolated perfused hearts were conducted using the Langendorff apparatus with Krebs–Henseleit buffer equilibrated with a 95% O2/5% CO_2_ gas mixture at 37 °C and at a constant pressure of 60 mm Hg to evaluate the induction of AFib as previously described. Langendorff-perfused hearts were stimulated with a pair of electrodes placed on the right atrium. All isolated hearts were stabilized for 5 min by perfusion at a constant flow before programmed electric stimulation. Teflon-coated (except for the tips) silver bipolar electrodes were placed on the appendages of the right atrium, LA, and left ventricle. AFib inducibility was tested by burst pacing methods. In burst pacing, we used the same protocol as transesophageal burst pacing, except for applying 2-second burst pacing. AFib was defined as rapid, irregular atrial excitations lasting at least 1 second.

### Statistics

Statistical analyses were performed using GraphPad Prism 9 (GraphPad Software) and SPSS statistical software (version 26.0). All data were presented as mean ± SD or SEM, and Median (IQR) after the Shapiro–Wilk test. For continuous variables, Student’s t-test or Mann-Whitney U test was used for 2 groups, as appropriate. One-way ANOVA followed by the Tukey-Kramer’s post hoc test was used for multiple group comparisons except where noted. The Fisher exact test was used to analyze categorical variables. The correlation of 2 continuous variables was evaluated with Pearson’s r, as appropriate. Two-way repeated-measures ANOVA was used for data in body weight. *p* < 0.05 was considered significant in all the experiments. Statistical parameters and number of animals per experiment are given in the figure legends.

### Reporting summary

Further information on research design is available in the Nature Portfolio Reporting Summary linked to this article.

## Supplementary information

Supplementary Information

Reporting summary

Transparent Peer Review file

## Source data

Source data

## Data Availability

All data generated during the current study are included in this manuscript and/or its supplementary information files. The Source Data file included in this paper contains data from individual experiments used to generate the final figures. Source data are provided with this paper. Metabolomics datasets have been deposited in the Metabolomics Workbench^65^ under Study ID ST004739. The data can be accessed via Project 10.21228/M8085J. Source data are provided with this paper.
