# Supplementary material for: Kynurenic acid mediates epicardial fat-induced lymphatic metabolic dysfunction in atrial fibrillation
Source: Nat Commun. 2026 May 19;17:6616. doi: 10.1038/s41467-026-72974-9 (PMC13381822; doi:10.1038/s41467-026-72974-9)
Supplement: Supplementary file 1 — Supplementary Information [file 41467_2026_72974_MOESM1_ESM.pdf]

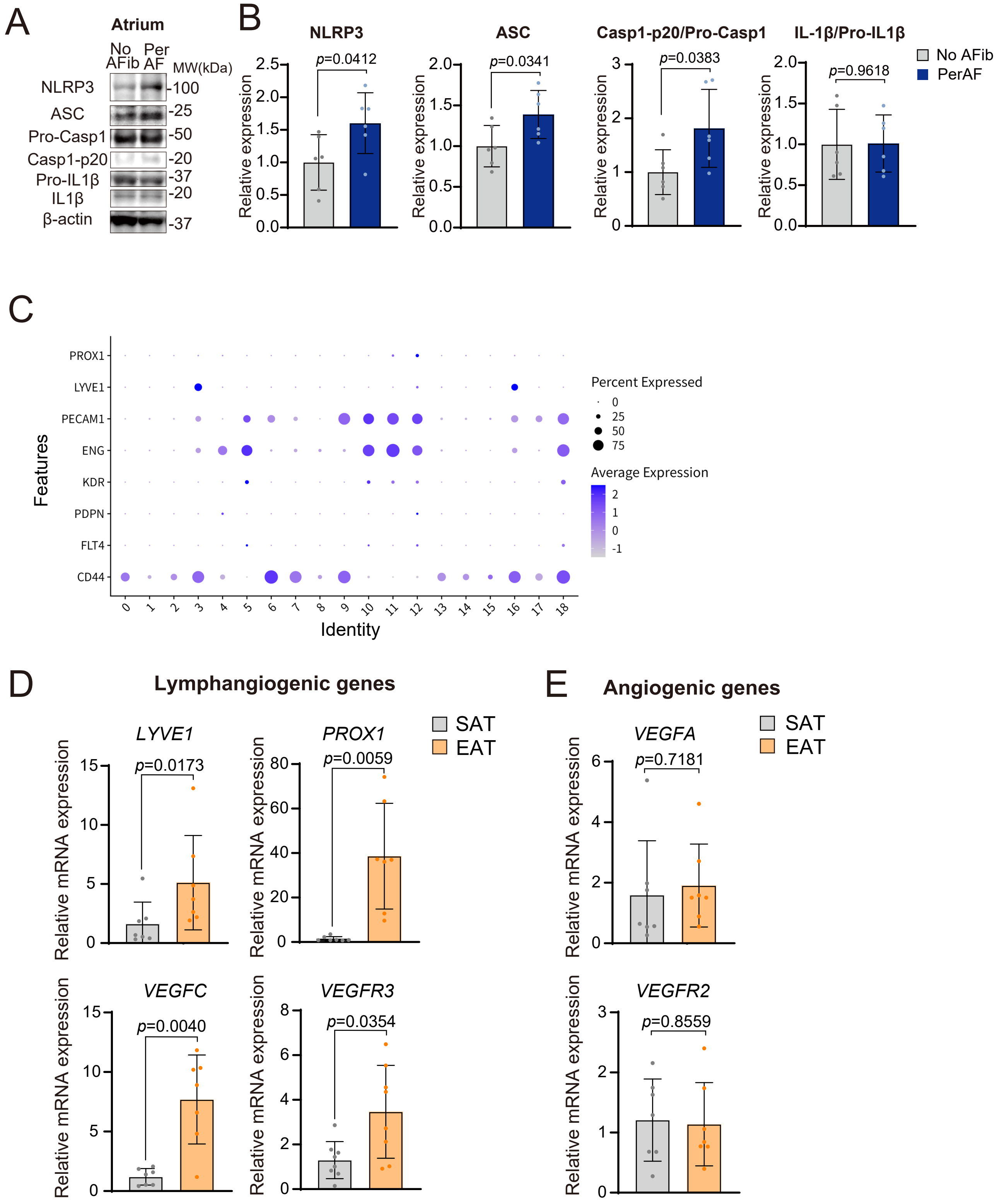


Supplemental Figure 1

**Supplemental Figure 1.**

**A.** Immunoblotting of NLRP3, ASC, Casp1-p20, Pro-Casp1, IL1β, and Pro-IL1β in the LAA of patients with No AFib and PerAF. β-actin was used as a loading control. Molecular weight (kDa) is shown on the right.

**B.** Quantification of NLRP3, ASC, Casp1-p20, Pro-Casp1, IL1β, and Pro-IL1β protein in (A). $n = 6$ for No AFib or PerAF, biologically independent samples. Data are mean ± SD.; $p$ value was determined by two-tailed unpaired Student's $t$-test.

**C.** Dot plot of mean expression of canonical lymphatic endothelial cell marker genes for each cluster from published single-cell RNA sequencing datasets in human left atrial tissues (Hulsmans et al., 2023), as indicated (Cluster 11 and 12: LECs cluster).

**D.** Relative mRNA levels of lymphangiogenic genes in the human SAT and EAT. Sample sizes for each gene were as follows (SAT/EAT): *LYVE1, n* = 7/7; *PROX1, n* = 7/7; *VEGFC, n* = 7/7; *VEGFR3*, *n* = 8/8, biologically independent samples. Data are mean ± SD.; $p$ value was determined by paired sample's $t$-test.

**E.** Relative mRNA levels of angiogenic genes in the human SAT and EAT. $n = 7$, biologically independent samples. Data are mean ± SD.; $p$ value was determined by paired sample's $t$-test.

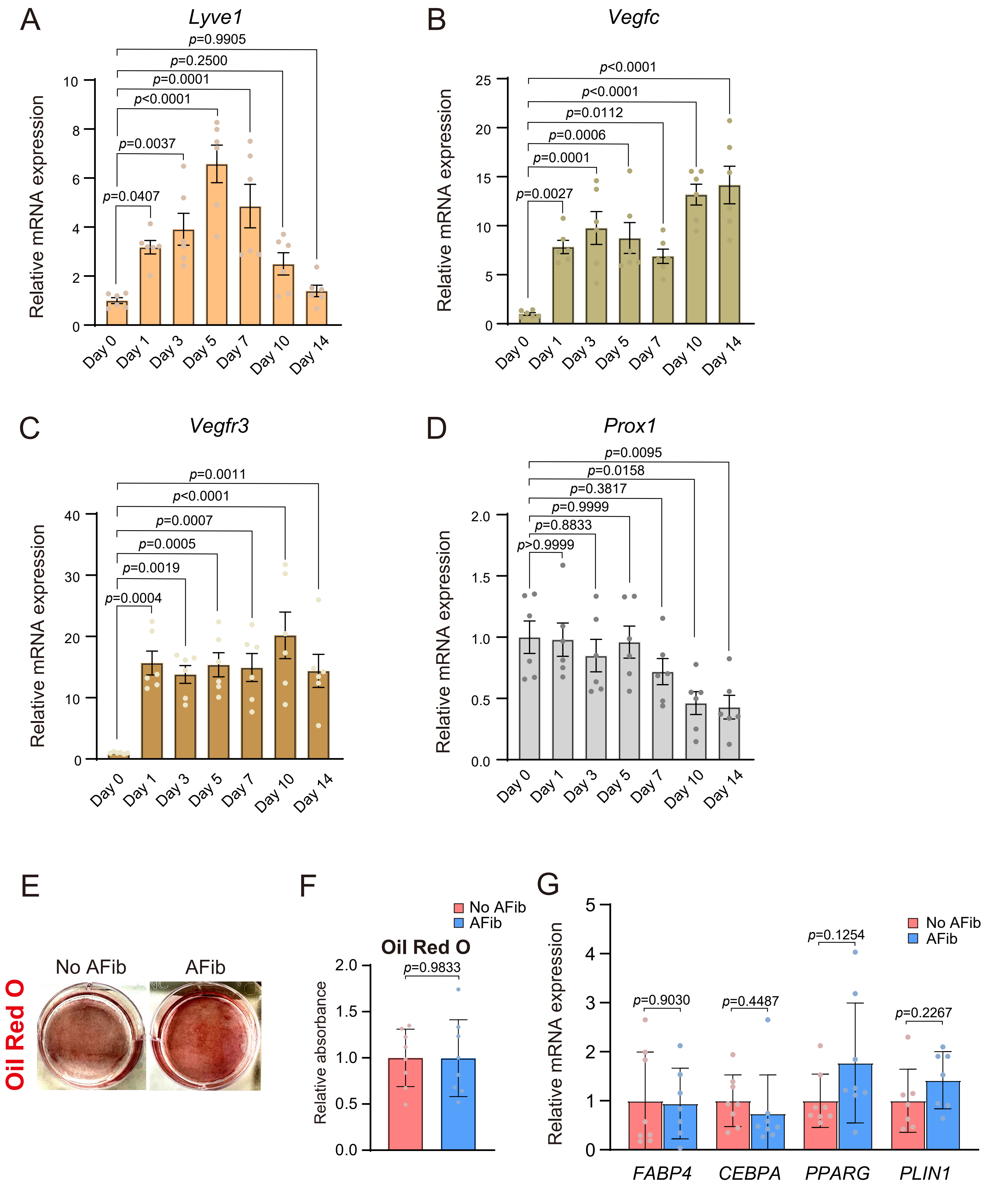


Supplemental Figure 2

**Supplemental Figure 2.**

**A.** Relative mRNA levels of *Lyve1* in the rat atria treated with Angiotensin II for 0, 1, 3, 5, 7, 10, 14 days. *n* = 6, biologically independent samples. Data are mean ± SEM.; *p* value was determined by one-way ANOVA followed by the Dunnett's *post hoc* test.

**B.** Relative mRNA levels of *Vegfc* in the rat atria treated with Angiotensin II for 0, 1, 3, 5, 7, 10, 14 days. *n* = 6, biologically independent samples. Data are mean ± SEM.; *p* value was determined by one-way ANOVA followed by the Dunnett's *post hoc* test..

**C.** Relative mRNA levels of *Vegfr3* in the rat atria treated with Angiotensin II for 0, 1, 3, 5, 7, 10, 14 days. *n* = 6, biologically independent samples. Data are mean ± SEM.; *p* value was determined by one-way ANOVA followed by the Dunnett's *post hoc* test.

**D.** Relative mRNA levels of *Prox1* in the rat atria treated with Angiotensin II for 0, 1, 3, 5, 7, 10, 14 days. *n* = 6, biologically independent samples. Data are mean ± SEM.; *p* value was determined by one-way ANOVA followed by the Dunnett's *post hoc* test..

**E.** Representative Oil Red O staining images of differentiated SVF from patients with No AFib and AFib.

**F.** Quantitative analysis of lipid accumulation in differentiated SVF from the patients with No AFib and AFib. Optical density was measured by using a spectrophotometer. *n* = 8 for both groups, biologically independent samples. Data are mean ± SD.; *p* value was determined by two-tailed unpaired Student's *t*-test.

**G.** Relative mRNA levels of adipogenic marker genes of differentiated human EAT SVF derived from No AFib or AFib patients. Sample sizes for each gene were as follows (No AFib/AFib): *FABP4, n* = 8/7; *CEBPA, n* = 8/8; *PPARG, n* = 8/8, *PLIN1, n* = 7/7, biologically independent samples. Data are mean ± SD.; *p* value was determined by two-tailed unpaired Student's *t*-test.

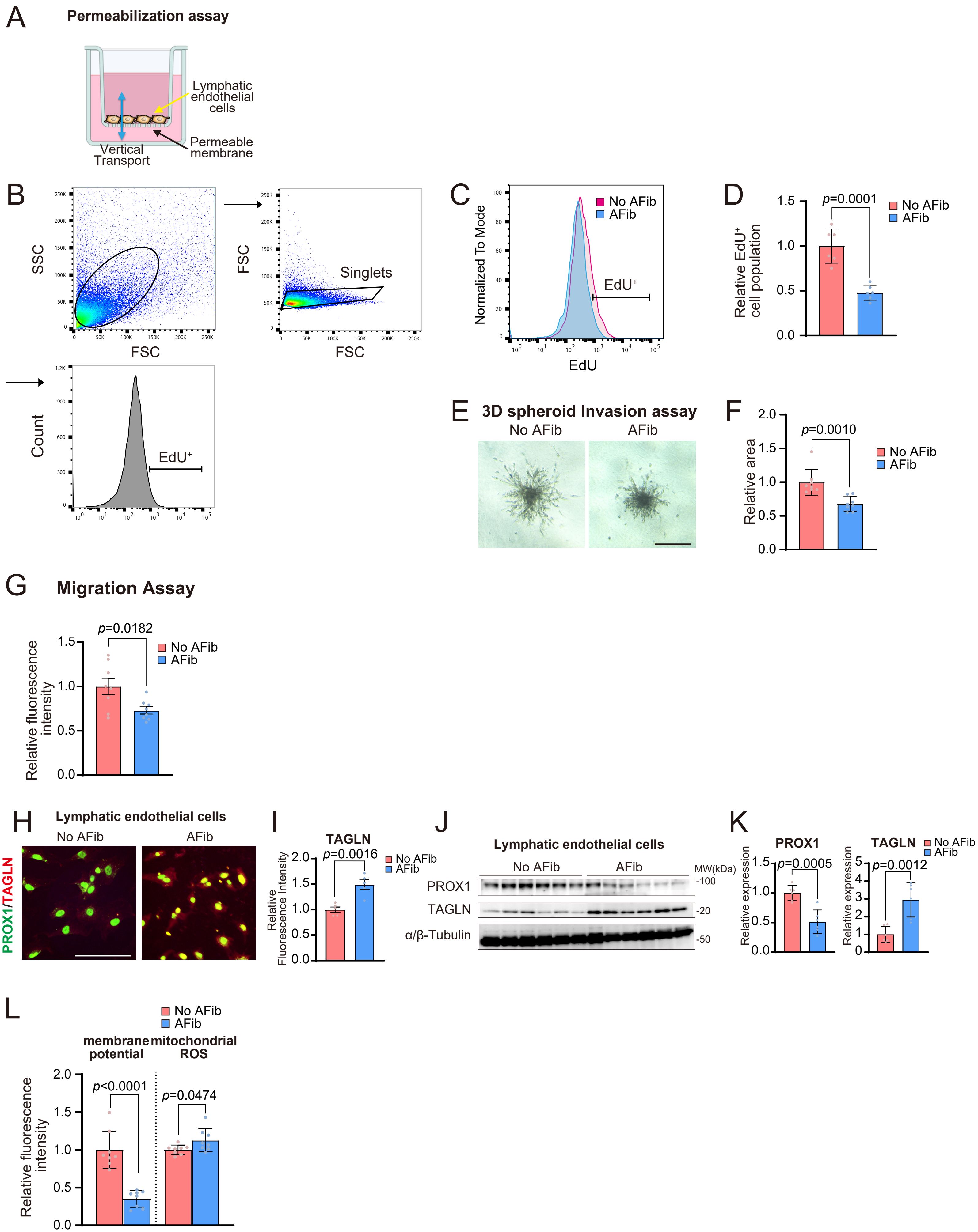


Supplemental Figure 3

**Supplemental Figure 3.**

**A.** Schematic illustration of permeabilization assay for LECs treated with EAT conditioned media. Created in BioRender. Takahashi, M. (2026) https://BioRender.com/viyx44r

**B.** Sequential gating strategy to discriminate EdU-positive LECs in vitro. We examined the proliferation of LECs based on the signal of EdU incorporation.

**C.** Representative FACS histogram images of EdU-positive cell population in LECs treated with No AFib or AFib-EAT conditioned media.

**D.** FACS-based quantification of EdU-positive cell population in LECs treated with No AFib or AFib-EAT conditioned media. $n = 6$ for each group, biologically independent samples. Data are mean ± SD.; $p$ value was determined by two-tailed unpaired Student's $t$-test.

**E.** Representative bright-field images of LECs spheroids embedded in invasion matrix treated with No AFib or AFib-EAT conditioned media. Scale bar, 500 µm

**F.** Quantitative analysis of area in (E). $n = 8$ for each group, biologically independent samples. Data are mean ± SD.; $p$ value was determined by two-tailed unpaired Student's $t$-test.

**G.** Quantitative analysis of migrated LECs treated with No AFib or AFib-EAT conditioned media. Migrated cells were quantified using CyQuant® GR dye and measured by fluorescence (RFU). $n = 8$ for each group, biologically independent samples. Data are mean ± SD.; $p$ value was determined by two-tailed unpaired Student's $t$-test.

**H.** Representative immunofluorescent staining for PROX1 (Green)/TAGLN (Red) of LECs treated with No AFib or AFib-EAT conditioned media. Scale bar, 100 µm.

**I.** Quantification of fluorescence intensity of TAGLN in LECs treated with No AFib or AFib-EAT conditioned media. $n = 5$ per group. Data are mean ± SD.; $p$ value was determined by two-tailed unpaired Student's $t$-test.

**J.** Immunoblotting of PROX1, and TAGLN in LECs treated with No AFib or AFib-EAT conditioned media. α/β-Tubulin was used as a loading control. Molecular weight (kDa) is shown on the right.

**K.** Quantification of PROX1, and TAGLN protein in (J). $n = 6$ for each group, biologically independent samples. Data are mean ± SD.; *p value* was determined by two-tailed unpaired Student's $t$-test.

**L.** Quantification of mitochondrial membrane potential measured as JC-1 red/green fluorescence intensity ratio, and mitochondrial superoxide levels measured as MitoBright ROS Deep Red fluorescence intensity in LECs treated with No AFib or AFib-EAT conditioned media. $n = 8$ for each group, biologically independent samples. Data are mean ± SD.; *p value* was determined by two-tailed unpaired Student's $t$-test.

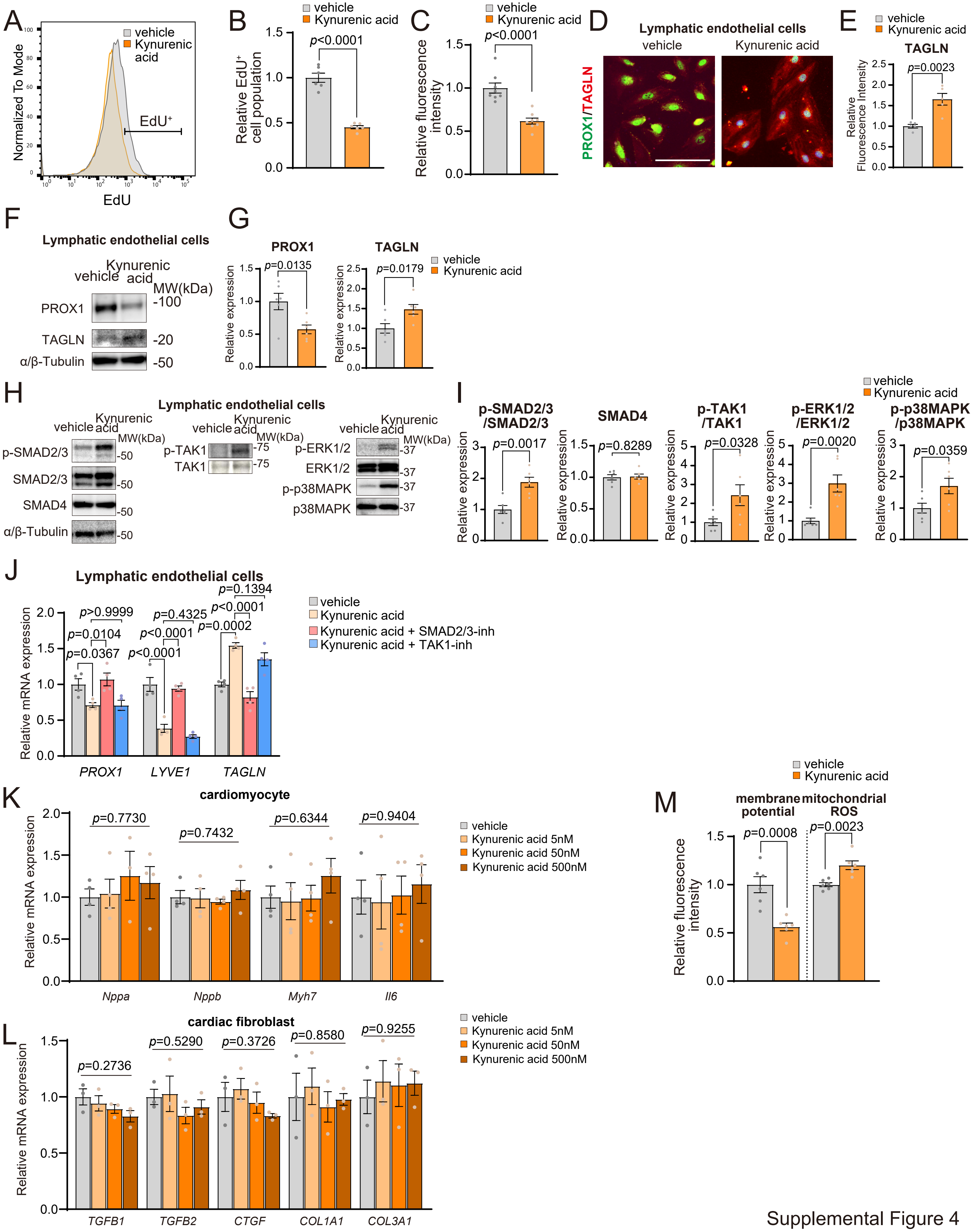


Supplemental Figure 4

**Supplemental Figure 4.**

**A.** Representative FACS histogram images of EdU-positive cell population in LECs treated with vehicle or Kynurenic acid.

**B.** FACS-based quantification of EdU-positive cell population in LECs treated with vehicle or Kynurenic acid. $n = 6$ *per* group, biologically independent samples. Data are mean ± SEM.; *p value* was determined by two-tailed unpaired Student's *t*-test.

**C.** Quantitative analysis of migrated LECs treated with vehicle or Kynurenic acid. Migrated cells were quantified using CyQuant® GR dye and measured by fluorescence (RFU). $n = 8$ *per* group, biologically independent samples. Data are mean ± SEM.; *p value* was determined by two-tailed unpaired Student's *t*-test.

**D.** Representative immunofluorescent staining for PROX1 (Green)/TAGLN (Red) of LECs treated with vehicle or Kynurenic acid. Scale bar, 100 µm. *p value*

**E.** Quantification of fluorescence intensity of TAGLN in LECs treated with vehicle or Kynurenic acid. n = 5 *per* group. Data are mean ± SEM.; *p value* was determined by two-tailed unpaired Student's *t*-test. *p value*

**F.** Immunoblotting of PROX1, and TAGLN in LECs treated with vehicle or Kynurenic acid. α/β-Tubulin was used as a loading control. Molecular weight (kDa) is shown on the right.

**G.** Quantification of PROX1, and TAGLN protein in (F). $n = 6$ *per* group, biologically independent samples. Data are mean ± SEM.; *p value* was determined by two-tailed unpaired Student's *t*-test.

**H.** Immunoblotting of p-SMAD2/3, SMAD2/3, SMAD4, p-TAK1, TAK1, p-ERK1/2, ERK1/2, p-p38MAPK, and p38MAPK in LECs treated with vehicle or Kynurenic acid. α/β-Tubulin was used as a loading control. Molecular weight (kDa) is shown on the right.

**I.** Quantification of p-SMAD2/3, SMAD2/3, SMAD4, p-TAK1, TAK1, p-ERK1/2, ERK1/2, p-p38MAPK, and p38MAPK protein in (A). $n = 6$ *per* group, biologically independent samples. Data are mean ± SEM.; *p* value was determined by two-tailed unpaired Student's *t*-test.

**J.** Relative mRNA levels of lymphatic endothelial marker, and mesenchymal marker genes in LECs treated by vehicle and kynurenic acid with SMAD2/3-inhibitor (SB431542) or TAK1-inhibitor (Takinib). $n = 4$ *per* group, biologically independent samples. Data are mean ± SEM.; *p* value was determined by one-way ANOVA followed by the Dunnett's *post hoc* test.

**K.** Relative mRNA levels of *Nppa, Nppb, Myh7,* and *Il6* in rat cardiomyocytes treated with Kynurenic acid. $n = 4$ *per* group, biologically independent samples. Data are mean ± SEM.; *p* value was determined by one-way ANOVA.

**L.** Relative mRNA levels of fibrogenic genes in human cardiac fibroblasts treated with Kynurenic acid. $n = 3$ *per* group, biologically independent samples. Data are mean ± SEM.; *p* value was determined by one-way ANOVA.

**M.** Quantification of mitochondrial membrane potential measured as JC-1 red/green fluorescence intensity ratio, and mitochondrial superoxide levels measured as MitoBright ROS Deep Red fluorescence intensity in LECs treated with vehicle or Kynurenic acid. $n = 6$ *per* group, biologically independent samples. Data are mean ± SEM.; *p* value was determined by two-tailed unpaired Student's *t*-test.

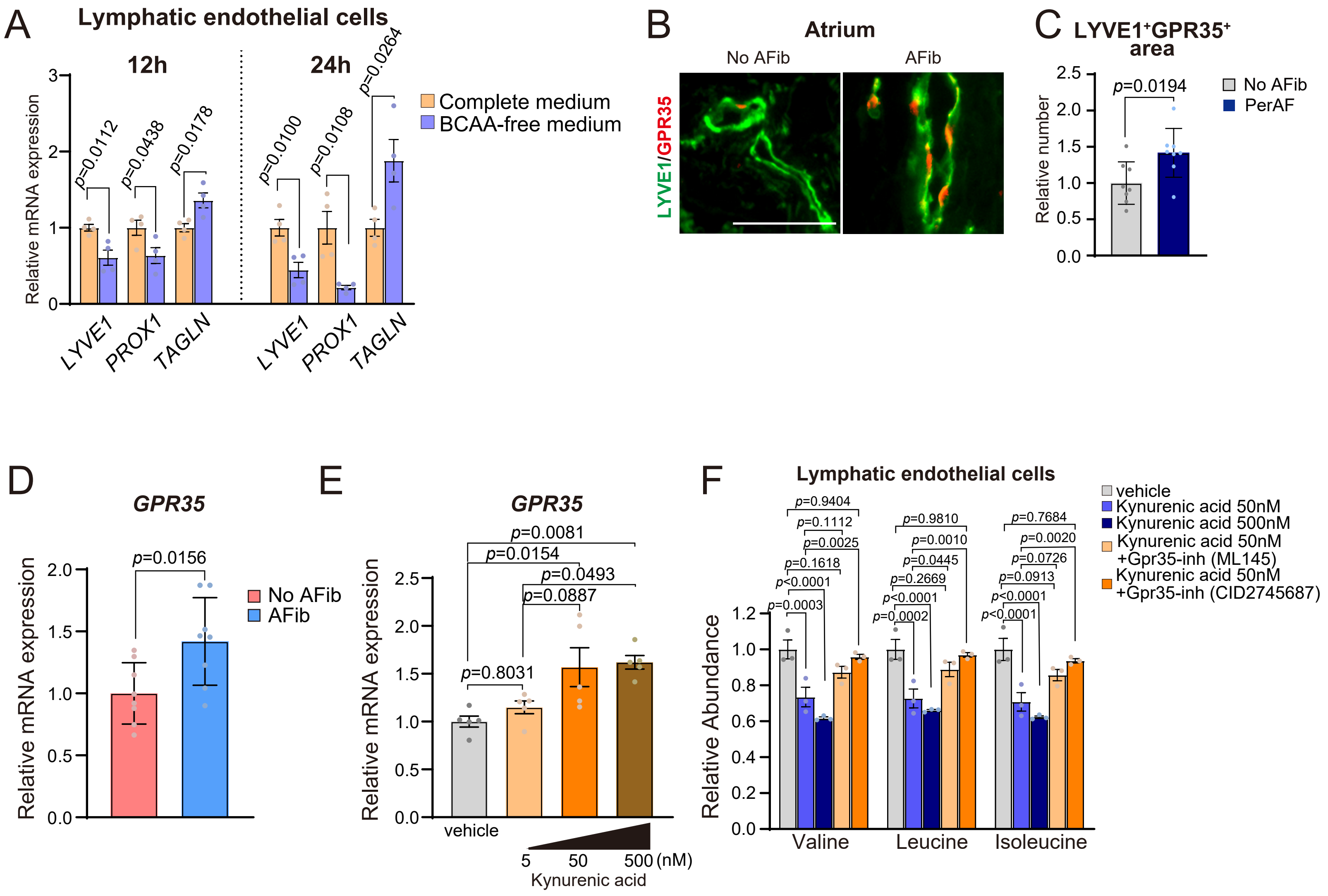


Supplemental Figure 5

**Supplemental Figure 5.**

- **A.** Relative mRNA levels of lymphatic endothelial marker, and mesenchymal marker genes in LECs cultured in complete medium or branched chain amino acid (BCAA)-free medium for 12 or 24 hours. $n = 4$ *per* group, biologically independent samples. Data are mean ± SEM.; *p* value was determined by two-tailed unpaired Student's *t*-test.
- **B.** Representative immunofluorescent staining for LYVE1 (Green)/GPR35 (Red) in LAA from the patients with No AFib or PerAF. Scale bar, 100 µm.
- **C.** Quantification of $LYVE1^{+}$ $GPR35^{+}$ area in (B). $n = 8$ for both groups. Data are mean ± SD.; *p* value was determined by two-tailed unpaired Student's *t*-test.
- **D.** Relative mRNA levels of *GPR35* in LECs treated with No AFIb or AFib-EAT conditioned media. $n = 8$ per group, biologically independent samples. Data are mean ± SD.; *p* value was determined by two-tailed unpaired Student's *t*-test.
- **E.** Relative mRNA levels of GPR35 in LECs treated with vehicle or Kynurenic acid (5 nM, 50 nM, and 500 nM). $n = 5$ *per* group, biologically independent samples. Data are mean ± SEM.; *p* value was determined by one-way ANOVA followed by the Tukey-Kramer's *post hoc* test.
- **F.** Relative abundance of BCAA in LEC treated with Kynurenic acid and with or without GPR35 inhibitors (ML145 at 50 nM and CID2745687 at 50 nM). $n = 3$ *per* group, biologically independent samples. Data are mean ± SEM.; *p* value was determined by two-way ANOVA followed by the Tukey-Kramer's *post hoc* test.

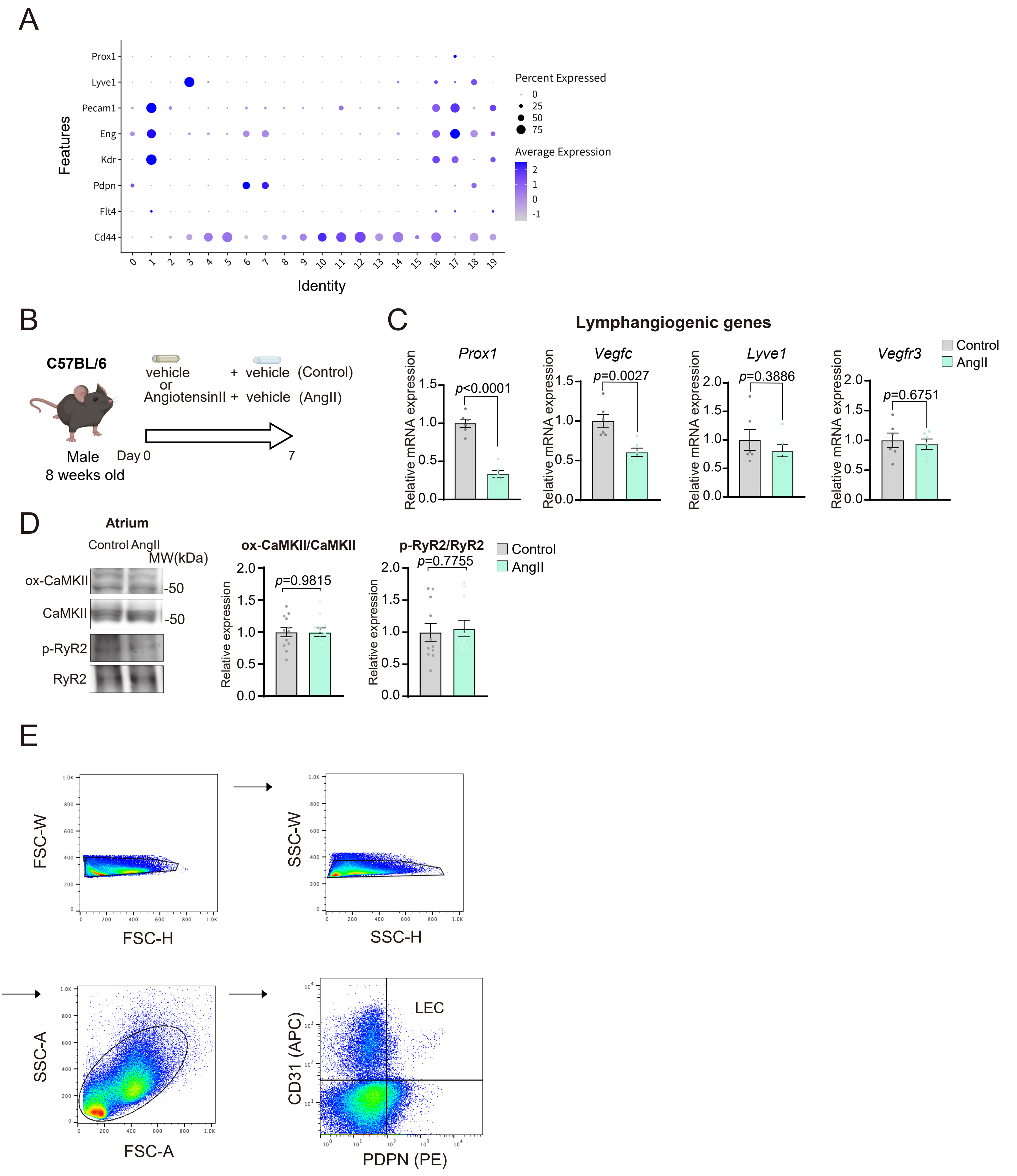


Supplemental Figure 6

**Supplemental Figure 6.**

**A.** Dot plot of mean expression of canonical lymphatic endothelial cell marker genes for each cluster from published single-cell RNA sequencing datasets in mice left atrial tissues (Hulsmans et al., 2023), as indicated (Cluster 17: LECs cluster).

**B.** Schematic illustration of the experiment procedure in mice. Male C57BL/6J mice at 8 weeks old received vehicle and/or Angiotensin II by osmotic pump for 7 days. Created in BioRender. Takahashi, M. (2026) https://BioRender.com/hcj216v

**C.** Relative mRNA levels of lymphangiogenic genes in left atria from vehicle and/or Angiotensin II treated mice for 7 days. $n = 6$ *per* group, biologically independent mice. Data are mean ± SEM.; *p value* was determined by two-tailed unpaired Student's *t*-test.

**D.** Left: Immunoblotting of oxidized-CaMKII, CaMKII, p-RyR2 and RyR2 in left atria from vehicle or Angiotensin II treated mice. Molecular weight (kDa) is shown on the right. Right: Quantification of oxidized-CaMKII, CaMKII, p-RyR2 and RyR2 protein. $n = 11$ for ox-CaMKII/CaMKII,n = 12 for p-RyR2/RyR2, biologically independent mice. Data are mean ± SEM.; *p* value was determined by two-tailed unpaired Student's *t*-test.

**E.** A sequential gating strategy to characterize lymphatic endothelial cells ($Lin^-$: $Cd31^+$: $Pdpn^+$ cells) from mice atrial tissues. Cell population (%) was calculated as frequency of parent.

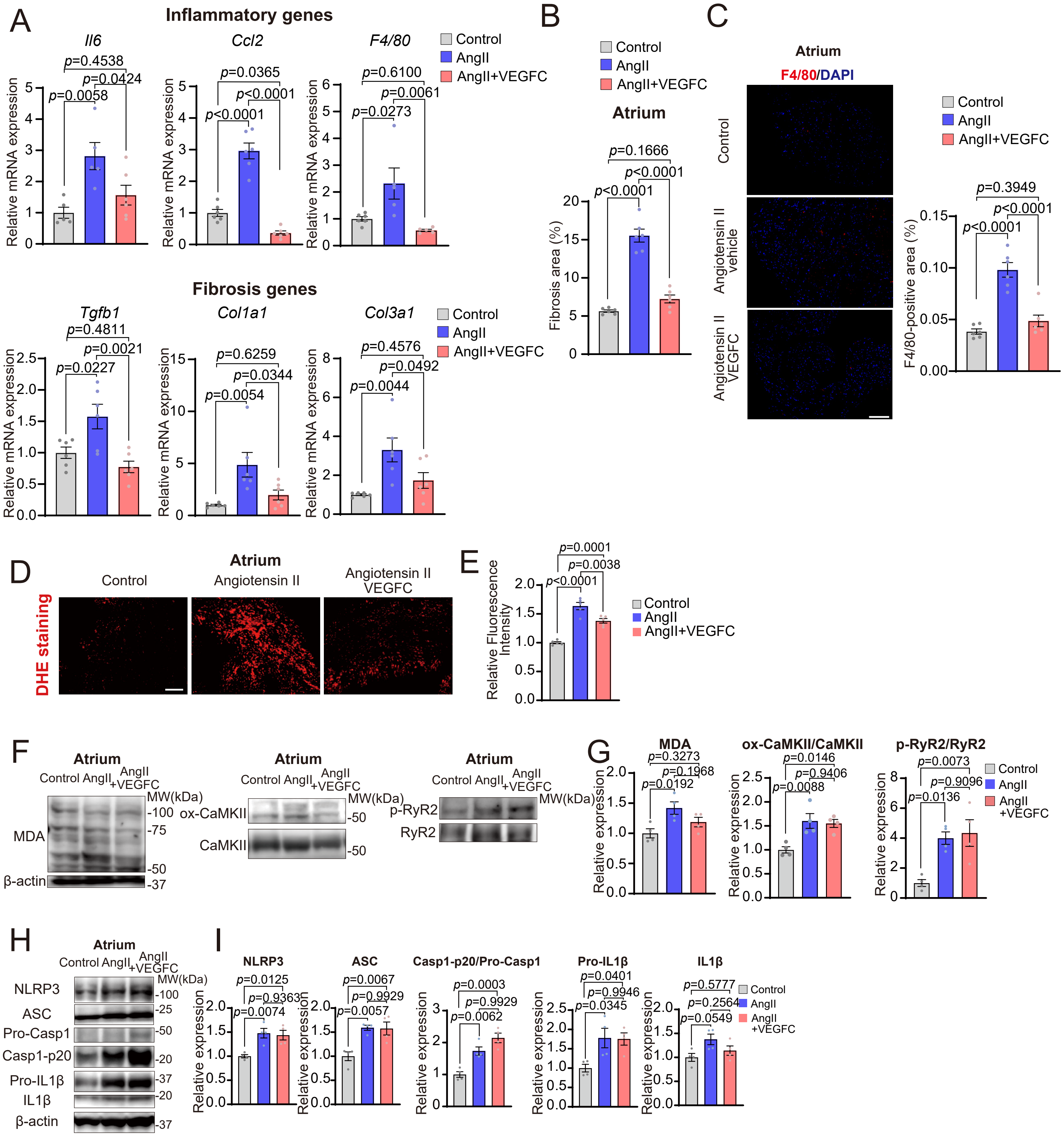


Supplemental Figure 7

**Supplemental Figure 7.**

**A.** Relative mRNA levels of inflammatory and fibrogenic genes in left atria from vehicle, AngiotensinII and/or VEGFC treated mice. Sample sizes for each gene were as follows (Control/AngII/AngII+VEGFC): Il6, n = 5/5/6; Ccl2, n = 6/6/6, F4/80, n=6/5/5, biologically independent mice. Data are mean ± SEM.; *p value* was determined by one-way ANOVA followed by the Tukey-Kramer's *post hoc* test.

**B.** Quantification of the atrial fibrosis area in left atria from vehicle, AngiotensinII and/or VEGFC treated mice. *n* = 6 *per* group, biologically independent mice. Data are mean ± SEM.; *p value* was determined by one-way ANOVA followed by the Tukey-Kramer's *post hoc* test.

**C.** Left: Representative immunofluorescent staining for F4/80 (Red)/DAPI (Blue) in left atria from vehicle, AngiotensinII and/or VEGFC treated mice. Scale bar, 100 µm. Right: Quantification of the F4/80-positive area. *n* = 6 *per* group. Data are mean ± SEM.; *p value* was determined by one-way ANOVA followed by the Tukey-Kramer's *post hoc* test.

**D.** Representative DHE fluorescent staining in left atria from vehicle, Angiotensin II and/or VEGFC treated mice. Scale bar, 100 µm.

**E.** Quantification of fluorescence intensity of DHE in left atria from vehicle, Angiotensin II and/or VEGFC treated mice. *n* = 5 *per* group, biologically independent mice. Data are mean ± SEM.; *p* value was determined by one-way ANOVA followed by the Tukey-Kramer's *post hoc* test.

**F.** Immunoblotting of MDA, oxidized-CaMKII, CaMKII, p-RyR2 and RyR2 in left atria from vehicle, Angiotensin II and/or VEGFC treated mice. β-actin was used as a loading control. Molecular weight (kDa) is shown on the right.

**G.** Quantification of MDA, oxidized-CaMKII, CaMKII, p-RyR2 and RyR2 protein in (F). *n* = 4 *per* group, biologically independent mice. Data are mean ± SEM.; p value was determined by one-way ANOVA followed by the Tukey-Kramer's *post hoc* test.

**H.** Immunoblotting of NLRP3, ASC, Casp1-p20, Pro-Casp1, IL1β, and Pro-IL1β in left atria from vehicle, Angiotensin II and/or VEGFC treated mice. β-actin was used as a loading control. Molecular weight (kDa) is shown on the right.

**I.** Quantification of NLRP3, ASC, Casp1-p20, Pro-Casp1, IL1β, and Pro-IL1β protein in (H). *n* = 4 *per* group, biologically independent mice. Data are mean ± SEM.; *p* value was determined by one-way ANOVA followed by the Tukey-Kramer's *post hoc* test.

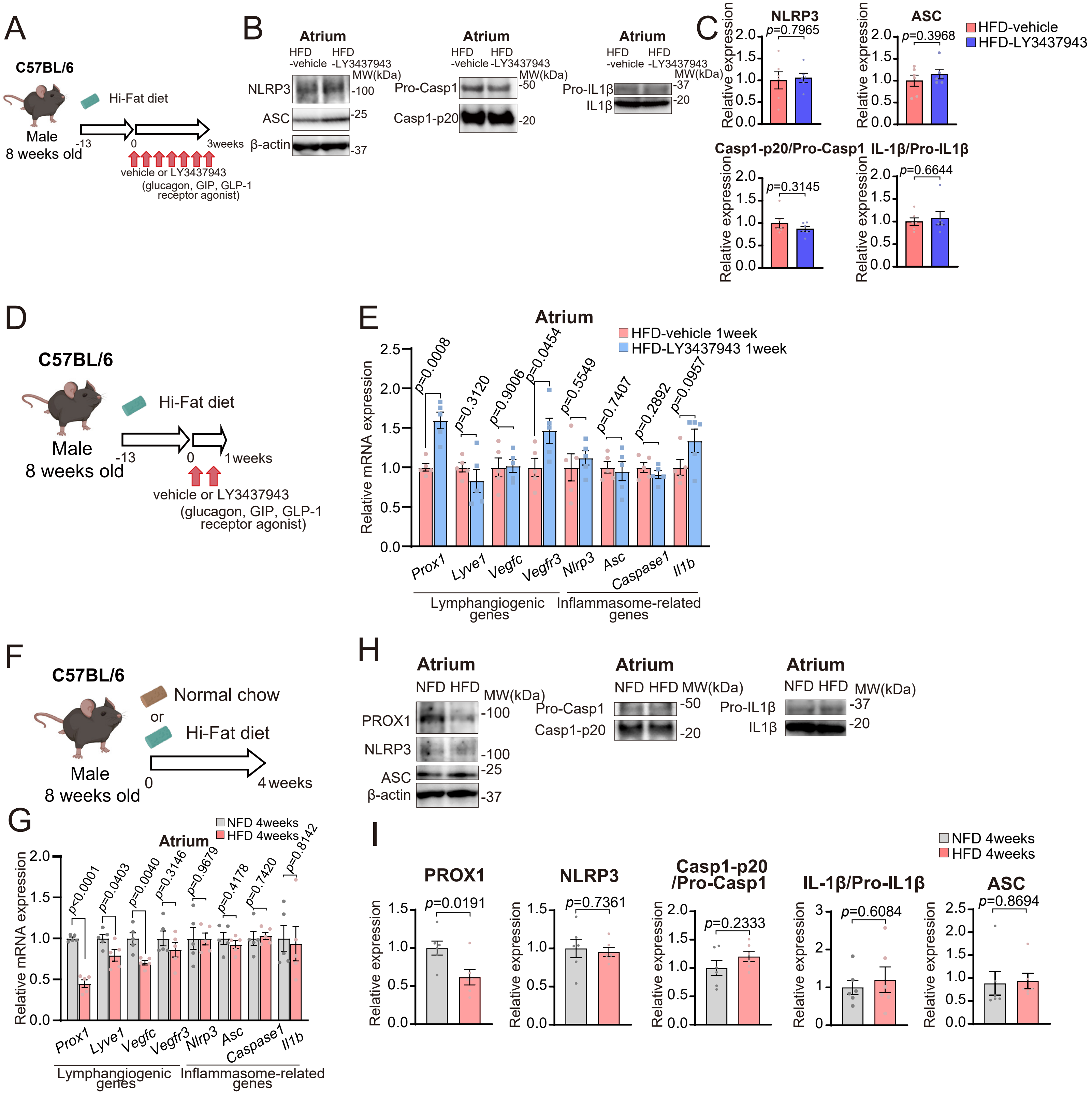


Supplemental Figure 8

**Supplemental Figure 8.**

**A.** Schematic illustration of the experiment for HFD-induced obese mice followed by administration of LY3437943. Male C57BL/6J mice at 8 weeks old were given access to Hi-fat diet (60 % fat) for 13 weeks. A subset of mice was then injected LY3437943 intraperitoneally every third day for 3 weeks. Created in BioRender. Takahashi, M. (2026) https://BioRender.com/cbmtw1j

**B.** Immunoblotting of NLRP3, ASC, Casp1-p20, Pro-Casp1, IL1β, and Pro-IL1β in left atria from HFD-induced obese mice with or without LY3437943 treatment. β-actin was used as a loading control. Molecular weight (kDa) is shown on the right.

**C.** Quantification of NLRP3, ASC, Casp1-p20, Pro-Casp1, IL1β, and Pro-IL1β protein in (B). $n = 6$ *per* group, biologically independent mice. Data are mean ± SEM.; $p$ value was determined by two-tailed unpaired Student's $t$-test.

**D.** Schematic illustration of the experiment for HFD-induced obese mice followed by administration of LY3437943. Male C57BL/6J mice at 8 weeks old were given access to Hi-fat diet (60 % fat) for 13 weeks. A subset of mice was then injected LY3437943 intraperitoneally every third day for 1 week. Created in BioRender. Takahashi, M. (2026) https://BioRender.com/noxi13s

**E.** Relative mRNA levels of lymphangiogenic and inflammasome-related genes in left atria from HFD-induced obese mice with or without LY3437943 treatment. $n = 5$ *per* group, biologically independent mice. Data are mean ± SEM.; $p$ value was determined by two-tailed unpaired Student's $t$-test.

**F.** Schematic illustration of the experiment for HFD-induced obese mice. Male C57BL/6J mice at 8 weeks old were given access to normal chow or Hi-fat diet (60 % fat) for 4 weeks. Created in BioRender. Takahashi, M. (2026) https://BioRender.com/bxquoem

**G.** Relative mRNA levels of lymphangiogenic and inflammasome-related genes in left atria from mice fed normal chow (NFD) or hi-fat diet (HFD). $n = 5$ *per* group, biologically independent mice. Data are mean ± SEM.; $p$ value was determined by two-tailed unpaired Student's $t$-test.

**H.** Immunoblotting of PROX1, NLRP3, ASC, Casp1-p20, Pro-Casp1, IL1β, and Pro-IL1β in left atria from mice fed NFD or HFD. β-actin was used as a loading control. Molecular weight (kDa) is shown on the right.

**I.** Quantification of PROX1, NLRP3, ASC, Casp1-p20, Pro-Casp1, IL1β, and Pro-IL1β protein in (H). $n = 6$ *per* group, biologically independent mice. Data are mean ± SEM.; p value was determined by two-tailed unpaired Student's $t$-test.

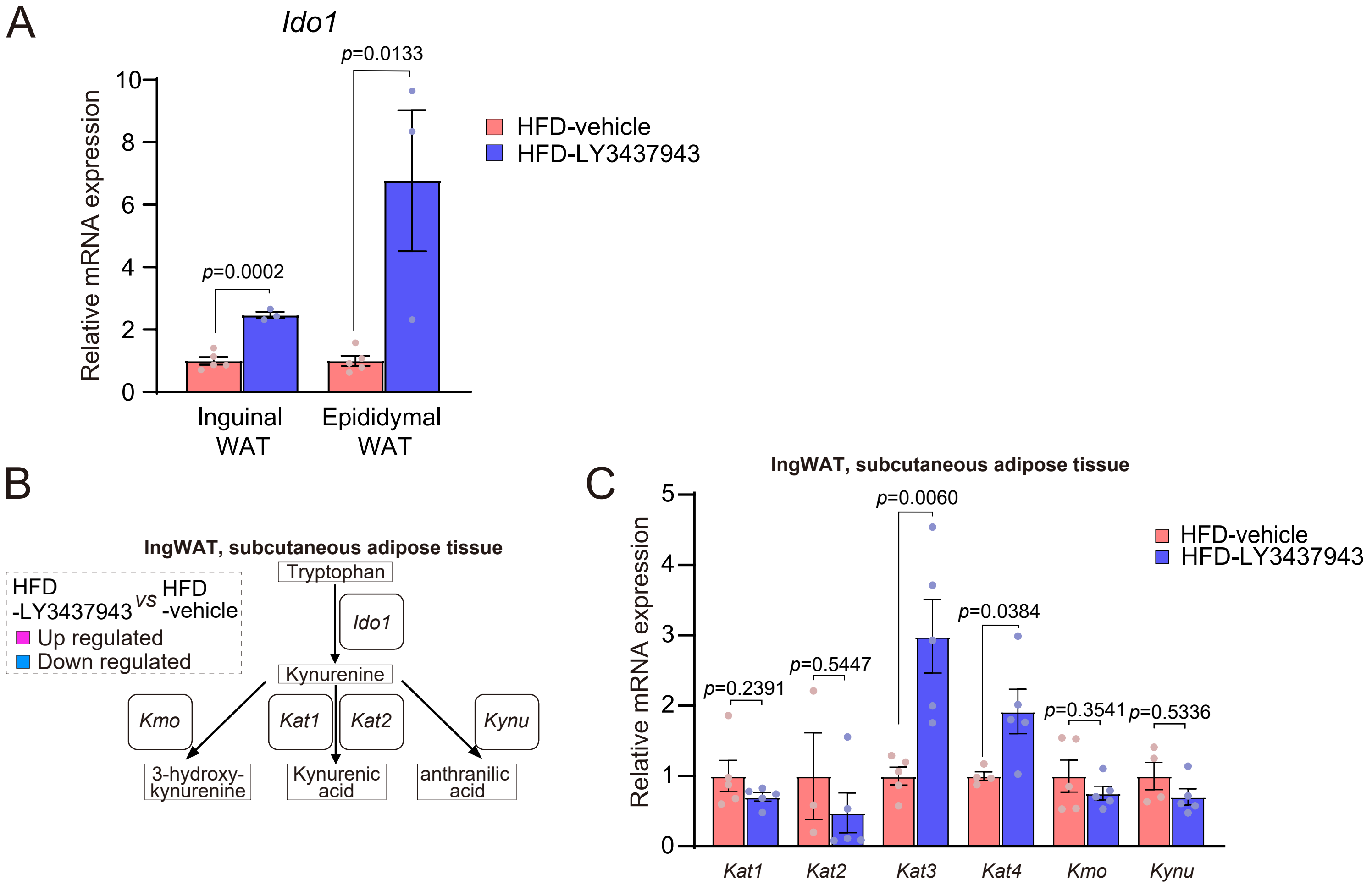
A
Ido1
Relative mRNA expression
p=0.0002
p=0.0133
10
8
6
4
2
0
Inguinal WAT
Epididymal WAT
HFD-vehicle
HFD-LY3437943
B
IngWAT, subcutaneous adipose tissue
HFD -LY3437943 vs HFD -vehicle
Up regulated
Down regulated
Tryptophan
Ido1
Kynurenine
Kmo
Kat1
Kat2
Kynu
3-hydroxy-kynurenine
Kynurenic acid
anthranilic acid
C
IngWAT, subcutaneous adipose tissue
Relative mRNA expression
5
4
3
2
1
0
p=0.2391
p=0.5447
p=0.0060
p=0.0384
p=0.3541
p=0.5336
Kat1
Kat2
Kat3
Kat4
Kmo
Kynu
HFD-vehicle
HFD-LY3437943

**Supplemental Figure 9.**

**A.** Relative mRNA levels of Ido1 in white adipose tissues from HFD-induced obese mice with or without LY3437943 treatment. *n* =5 for HFD-vehicle, n = 3 for HFD-LY3437943, biologically independent mice. Data are mean ± SEM.; p value was determined by two-tailed unpaired Student's *t*-test.

**B.** Schematic illustration of RT-PCR analysis related to kynurenic acid metabolism in IngWAT,

**C.** Relative mRNA levels of kynurenic acid metabolism related genes in IngWAT from obese mice with or without LY3437943 treatment. Sample sizes for each gene were as follows (HFD-vehicle/HFD-LY3437943): *Kat1, n* = 5/5; *Kat2, n* = 3/5; *Kat3, n* = 5/5; *Kat4, n* = 4/5; *Kmo, n* = 5/5; and *Kynu, n*= 4/5, biologically independent mice. Data are mean ± SEM.; *p* value was determined by two-tailed unpaired Student's *t*-test.

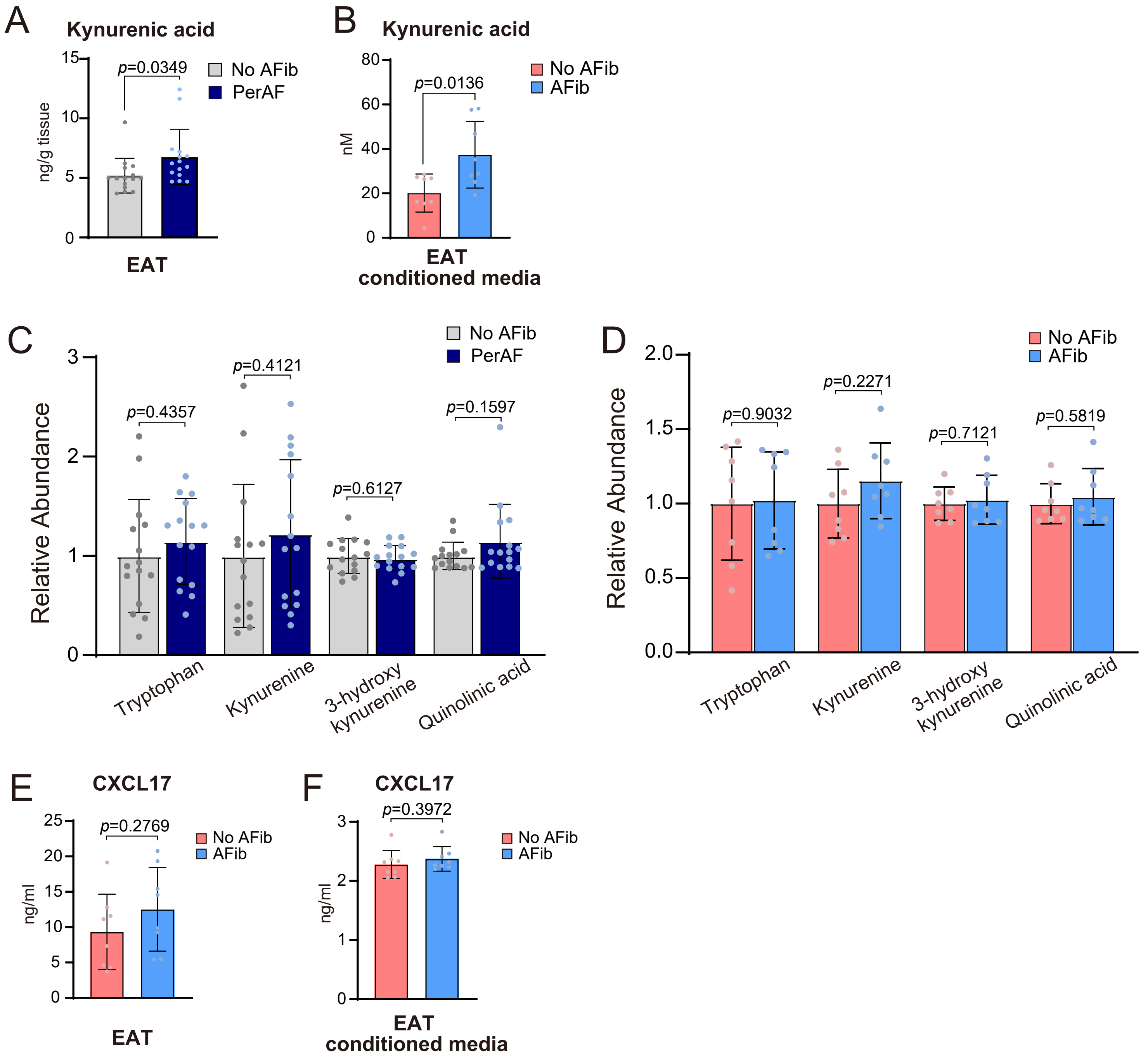


Supplemental Figure 10

**Supplemental Figure 10.**

**A.** Kynurenic acid concentrations in EAT from No AFib or PerAF patients. $n = 15$ for both groups, biologically independent samples. Data are mean ± SD.; $p$ value was determined by two-tailed unpaired Student's $t$-test.

**B.** Kynurenic acid concentrations in No AFib or AFib-EAT conditioned media. n = 8 for both groups, biologically independent samples. Data are mean ± SD.; $p$ value was determined by two-tailed unpaired Student's $t$-test.

**C.** Relative abundance of Kynurenine pathway metabolites in EAT from No AFib or PerAF patients. $n = 15$ for both groups, biologically independent samples. Data are mean ± SD.; $p$ value was determined by two-tailed unpaired Student's $t$-test.

**D.** Relative abundance of Kynurenine pathway metabolites in No AFib or AFib-EAT conditioned media. $n = 8$ for both groups, biologically independent samples. Data are mean ± SD.; $p$ value was determined by two-tailed unpaired Student's $t$-test.

**E.** Concentration of CXCL17 in EAT from patients with No AFib and AFib. $n = 8$ for both groups. Data are mean ± SD.; $p$ value was determined by two-tailed unpaired Student's $t$-test.

**F.** Concentration of CXCL17 in No AFib or AFib-EAT conditioned media. $n = 8$ for both groups. Data are mean ± SD.; $p$ value was determined by two-tailed unpaired Student's $t$-test.

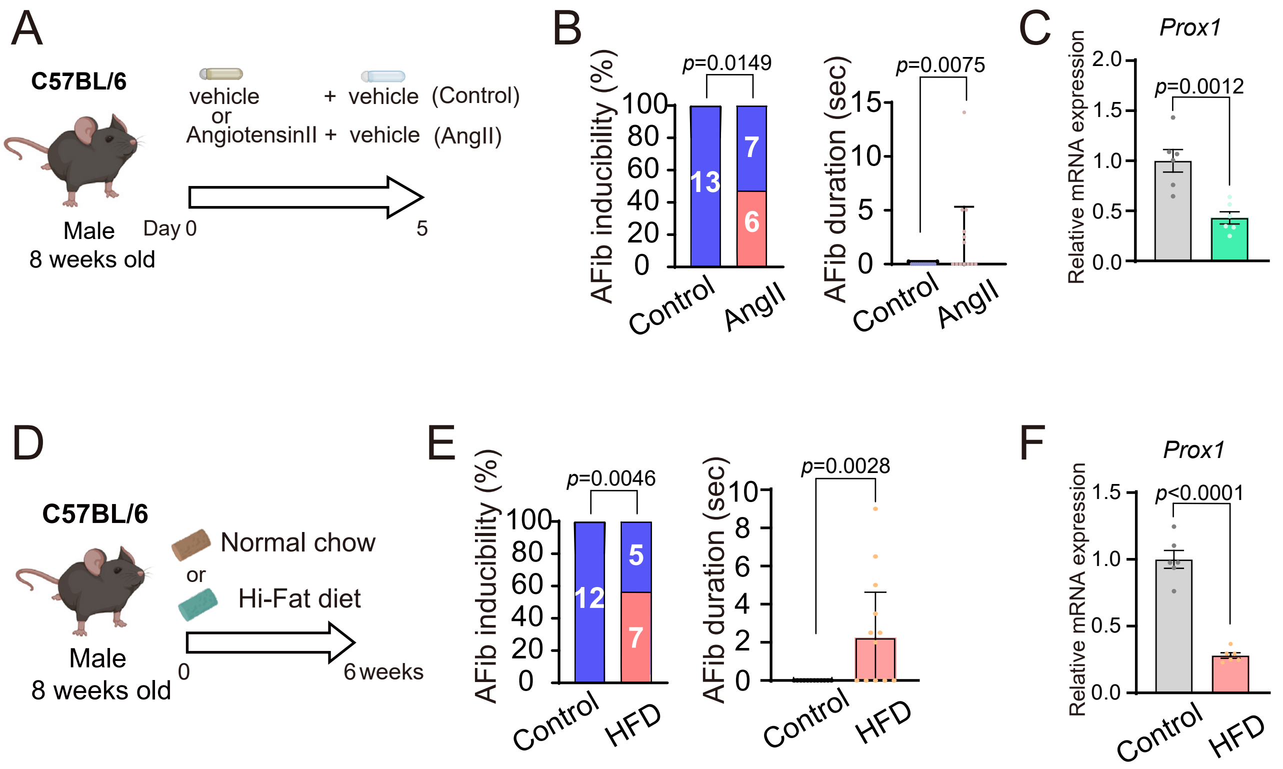


Supplemental Figure 11

**Supplemental Figure 11.**

**A.** Schematic illustration of the experiment procedure in mice. Male C57BL/6J mice at 8 weeks old received vehicle and/or Angiotensin II by osmotic pump for 5 days. Created in BioRender. Takahashi, M. (2026) https://BioRender.com/tvx9vui

**B.** Left: Atrial fibrillation inducibility by transesophageal burst pacing. $n$ = 13 *per* group analysed by Fisher's exact test. Right: Atrial fibrillation duration induced by transesophageal pacing. $n$ = 13 *per* group. Data are median (IQR).; $p$ value was determined by Mann Whitney U test.

**C.** Relative mRNA levels of *Prox1* in left atria from vehicle and/or Angiotensin II treated mice for 5 days. $n$ = 6 *per* group, biologically independent mice. Data are mean ± SEM.; *p value* was determined by two-tailed unpaired Student's $t$-test.

**D.** Schematic illustration of the experiment for HFD-induced obese mice. Male C57BL/6J mice at 8 weeks old were given access to normal chow or Hi-fat diet (60 % fat) for 6 weeks. Created in BioRender. Takahashi, M. (2026) https://BioRender.com/4jepm08

**E.** Left: Atrial fibrillation inducibility by transesophageal burst pacing. $n$ = 12 *per* group analysed by Fisher's exact test. Right: Atrial fibrillation duration induced by transesophageal pacing. $n$ = 12 *per* group. Data are median (IQR).; $p$ value was determined by Mann Whitney U test.

**F.** Relative mRNA levels of *Prox1* in left atria from mice fed normal chow (NFD) or hi-fat diet (HFD). $n$ = 6 *per* group, biologically independent mice. Data are mean ± SEM.; *p value* was determined by two-tailed unpaired Student's $t$-test.

**Table S1 (related to Figure 1A):** Subject information for experiments of isolated left atrial tissue in human.

| | No AFib (*n*=16) | Paroxysmal AFib (*n*=13) | Persistent AFib (*n*=13) | *p* value |
|---|---|---|---|---|
| Age (years) | 73.1 ± 10.4 | 69.8 ± 12.6 | 71.2 ± 6.5 | 0.68 |
| Sex | | | | |
| Male | 13 (81) | 6 (46) | 6 (46) | 0.20 |
| Female | 3 (19) | 7 (54) | 7 (54) | 0.20 |
| BMI ($kg/m^2$) | 23.1 (21.9-25.1) | 23.7(20.5-25.6) | 21.4 (20.6-28.3) | 0.84 |
| History of present and past illness | | | | |
| Hypertension | 10 (63) | 8 (62) | 7 (54) | 0.88 |
| Diabetes mellitus | 5 (31) | 3 (23) | 3 (23) | 0.84 |
| Dyslipidemia | 10 (63) | 7 (54) | 4 (31) | 0.22 |
| Coronary artery disease | 10 (63) | 4 (31) | 1 (8) | <0.01 |
| Cerebral infarction | 1 (6) | 3 (23) | 5 (38) | 0.11 |
| Smoking | 9 (56) | 4 (31) | 3 (23) | 0.74 |
| Medications | | | | |
| ACE inhibitors/ARB/ARNI | 7 (44) | 1 (8) | 6 (46) | 0.06 |
| β-blockers | 8 (50) | 10 (77) | 7 (54) | 0.22 |
| Calcium channel blockers | 5 (31) | 5 (38) | 4 (31) | 0.89 |
| Digoxin | 0 (0) | 1 (8) | 2 (15) | 0.28 |
| Diuretics | 1 (6) | 9 (69) | 10 (77) | <0.01 |
| Warfarin | 1 (6) | 4 (31) | 6 (46) | 0.05 |
| DOAC | 0 (0) | 5 (38) | 7 (54) | <0.01 |
| Statin | 10 (63) | 6 (46) | 3 (23) | 0.11 |
| SGLT2i | 2 (12) | 0 (0) | 0 (0) | 0.18 |
| $CHADS_2$ score | 1.8 ± 1.1 | 2.7 ± 1.1 | 3.0 ± 1.5 | 0.03 |
| $CHA_2DS_2$-VASc score | 3.3 ± 1.4 | 4.2 ± 1.2 | 4.4 ± 1.4 | 0.09 |
| Surgical procedure | | | | |
| Valve replacement/repair | 1 (6) | 8 (62) | 9 (69) | <0.01 |
| Aorta replacement | 4 (25) | 0 (0) | 1 (8) | 0.10 |
| CABG | 9 (56) | 0 (0) | 1 (8) | <0.01 |
| more than 2 procedures | 2 (12) | 5 (38) | 2 (15) | 0.19 |
| Hb (g/dL) | 13.4 ± 1.6 | 11.8 ± 1.9 | 12.9 ± 1.7 | 0.05 |
| CRP (mg/dl) | 0.15 (0.07-0.61) | 0.05 (0.04-0.49) | 0.12 (0.02-0.12) | 0.40 |

| | | | | |
|---|---|---|---|---|
| LDL-chol (mg/dl) | 103 ± 38 | 94 ± 19 | 94 ± 25 | 0.66 |
| HDL-chol (mg/dl) | 47 ± 13 | 53 ± 16 | 52 ± 12 | 0.50 |
| TG (mg/dl) | 105 (65-207) | 91 (55-151) | 99 (58-125) | 0.58 |
| Cr (mg/dl) | 0.85 (0.75-0.98) | 0.84 (0.74-1.24) | 1.18 (0.87-1.42) | 0.11 |
| eGFR (ml/min/1.73 $m^2$) | 65 (57-78) | 53 (46-66) | 48 (33-55) | <0.01 |
| BNP (pg/ml) | 42 (17-86) | 247 (128-599) | 198 (149-258) | <0.01 |
| HbA1c (%) | 5.85 (5.50-6.40) | 5.8 (5.45-6.18) | 6.0 (5.75-6.50) | 0.537 |
| LAD (mm) | 36 (34-39) | 48 (39-53) | 52 (47-59) | <0.01 |
| LVDd (mm) | 47 (42-51) | 50 (46-55) | 52 (46-66) | 0.45 |
| LVEF (%) | 62 (56-68) | 68 (59-70) | 50 (42-67) | 0.19 |
| E/e' | 9.9 (8.5-14.7) | 21.4 (17.2-29.9) | 22.1 (13.5-33.4) | <0.01 |

Data are given as mean ± SD, median (IQR) or *n* (%). *p* value was determined by two-tailed Fisher's exact test, one-way ANOVA and the Kruskal-Wallis test, as appropriate. ACE; angiotensin converting enzyme, ARB; angiotensin receptor blocker, ARNI; angiotensin receptor-neprilysin inhibitor, BMI; body mass index, BNP; brain natriuretic peptide, CABG; coronary artery bypass grafting, CRP; C-reactive protein, DOAC; direct oral anticoagulant, eGFR; estimated glomerular filtration rate, Hb; hemoglobin, HbA1c; hemoglobin A1c, HDL; high-density lipoprotein, LAD; left atrial diameter, LDL; low-density lipoprotein, LVDd; left ventricular diastolic diameter, LVEF; left ventricular ejection fraction, SGLT2i; sodium-glucose cotransporter-2 inhibitor, TG; triglycerides.

**Table S2 (related to Figure 1N):** Subject information for organo-culture experiments by using isolated epicardial adipose tissue in human.

| | No AFib ($n$=5) | Persistent AFib ($n$=11) | $p$ value |
|---|---|---|---|
| Age (years) | 82.2 ± 6.1 | 72 ± 5.8 | <0.01 |
| Sex | | | |
| Male | 3 (60) | 10 (91) | 0.21 |
| Female | 2 (40) | 1 (9) | 0.21 |
| BMI (kg/m$^2$) | 22.4 ± 1.8 | 22.6 ± 3.3 | 0.89 |
| History of present and past illness | | | |
| Hypertension | 4 (80) | 6 (55) | 0.59 |
| Diabetes mellitus | 2 (40) | 2 (18) | 0.55 |
| Dyslipidemia | 3 (60) | 4 (36) | 0.60 |
| Coronary artery disease | 3 (60) | 2 (18) | 0.25 |
| Cerebral infarction | 1 (20) | 1 (9) | 1.00 |
| Smoking | 1 (20) | 4 (36) | 1.00 |
| Medications | | | |
| ACE inhibitors/ARB/ARNI | 3 (60) | 10 (91) | 0.21 |
| β-blockers | 2 (40) | 7 (64) | 0.60 |
| Calcium channel blockers | 3 (60) | 3 (27) | 0.30 |
| Digoxin | 0 (0) | 0 (0) | 1.00 |
| Diuretics | 0 (0) | 6 (55) | 0.09 |
| Warfarin | 0 (0) | 1 (9) | 1.00 |
| DOAC | 0 (0) | 8 (73) | 0.03 |
| Statin | 3 (60) | 2 (18) | 0.25 |
| SGLT2i | 0 (0) | 0 (0) | 1.00 |
| CHADS$_2$ score | 2.8 ± 0.8 | 2.0 ± 1.1 | 0.17 |
| Surgical procedure | | | |
| Valve replacement/repair | 1 (20) | 5 (45) | 0.59 |
| Aorta replacement | 1 (20) | 0 (0) | 1.00 |

| | | | |
|---|---|---|---|
| CABG | 3 (60) | 1 (9) | 0.06 |
| more than 2 procedures | 0 (0) | 5 (45) | 0.12 |
| Hb (g/dL) | 13.1 ± 1.5 | 13.3 ± 1.4 | 0.78 |
| CRP (mg/dl) | 0.15 (0.05-2.64) | 0.05 (0.01-0.08) | 0.15 |
| LDL-chol (mg/dl) | 94 ± 30 | 99 ± 36 | 0.77 |
| HDL-chol (mg/dl) | 53 ± 16 | 59 ± 16 | 0.48 |
| TG (mg/dl) | 133 (64-305) | 66 (60-169) | 0.38 |
| Cr (mg/dl) | 0.76 (0.48-0.94) | 0.92 (0.68-1.15) | 0.04 |
| eGFR (ml/min/1.73 $m^2$) | 75.4 ± 18.7 | 56.8 ± 12.3 | 0.03 |
| HbA1c (%) | 6.18 ± 0.87 | 6.1 ± 0.47 | 0.82 |
| LAD (mm) | 35 (31-37) | 53 (47-57) | <0.01 |
| LVDd (mm) | 42 (37-46) | 51 (50-56) | <0.01 |
| LVEF (%) | 66 ± 2.9 | 69 ± 7.9 | 0.53 |
| E/e' | 9.7 (8.7-21.6) | 16.6 (13.2-24.2) | 0.12 |

Data are given as mean ± SD, median (IQR) or *n* (%). *p* value was determined by two-tailed Fisher's exact test, two-tailed unpaired Student's *t*-test and two-tailed Mann-Whitney U test, as appropriate.

ACE; angiotensin converting enzyme, ARB; angiotensin receptor blocker, ARNI; angiotensin receptor-neprilysin inhibitor, BMI; body mass index, BNP; brain natriuretic peptide, CABG; coronary artery bypass grafting, CRP; C-reactive protein, DOAC; direct oral anticoagulant, eGFR; estimated glomerular filtration rate, Hb; hemoglobin, HbA1c; hemoglobin A1c, HDL; high-density lipoprotein, LAD; left atrial diameter, LDL; low-density lipoprotein, LVDd; left ventricular diastolic diameter, LVEF; left ventricular ejection fraction, SGLT2i; sodium-glucose cotransporter-2 inhibitor, TG; triglycerides.

**Table S3 (related to Figure 2A):** Subject information for lymphatic endothelial cell experiments by using isolated epicardial adipose tissue in human.

| | No AFib ($n$=8) | Persistent AFib ($n$=8) | $p$ value |
|---|---|---|---|
| Age (years) | 72 ± 9.7 | 74 ± 8.1 | 0.64 |
| Sex | | | |
| Male | 4 (50) | 1 (13) | 0.28 |
| Female | 4 (50) | 7 (88) | 0.28 |
| BMI (kg/m$^2$) | 24.6 ± 2.6 | 21.9 ± 3.7 | 0.11 |
| History of present and past illness | | | |
| Hypertension | 2 (25) | 4 (50) | 0.61 |
| Diabetes mellitus | 6 (75) | 6 (75) | 1.00 |
| Dyslipidemia | 6 (75) | 2 (25) | 0.13 |
| Coronary artery disease | 4 (50) | 2 (25) | 0.61 |
| Cerebral infarction | 0 (0) | 0 (0) | 1.00 |
| Smoking | 3 (38) | 5 (63) | 0.62 |
| Medications | | | |
| ACE inhibitors/ARB/ARNI | 5 (63) | 7 (88) | 0.57 |
| β-blockers | 2 (25) | 5 (63) | 0.32 |
| Calcium channel blockers | 4 (50) | 2 (25) | 0.61 |
| Digoxin | 0 (0) | 0 (0) | 1.00 |
| Diuretics | 3 (38) | 7 (88) | 0.12 |
| Warfarin | 0 (0) | 1 (12) | 1.00 |
| DOAC | 0 (0) | 6 (75) | <0.01 |
| Statin | 5 (63) | 1 (12) | 0.12 |
| SGLT2i | 1 (13) | 2 (25) | 1.00 |
| $CHADS_2$ score | 1.75 ± 0.9 | 2.3 ± 1.2 | 0.35 |
| Surgical procedure | | | |
| Valve replacement/repair | 4 (50) | 7 (88) | 0.28 |
| Aorta replacement | 3 (38) | 0 (0) | 0.20 |

| | | | |
|---|---|---|---|
| CABG | 4 (50) | 2 (25) | 0.61 |
| more than 2 procedures | 3 (38) | 2 (25) | 1.00 |
| Hb (g/dL) | 12.2 ± 1.8 | 12.7 ± 1.7 | 0.54 |
| CRP (mg/dl) | 0.20 (0.06-0.55) | 0.05 (0.02-0.31) | 0.16 |
| LDL-chol (mg/dl) | 91 ± 41 | 77 ± 21 | 0.40 |
| HDL-chol (mg/dl) | 48 ± 12 | 60 ± 19 | 0.16 |
| TG (mg/dl) | 180 (93-225) | 62 (45-77) | <0.01 |
| Cr (mg/dl) | 1.34 ± 0.73 | 1.27 ± 0.32 | 0.791 |
| eGFR (ml/min/1.73 $m^2$) | 44.0 ± 18.4 | 42.7 ± 12.7 | 0.875 |
| HbA1c (%) | 6.18 ± 0.57 | 6.0 ± 0.59 | 0.612 |
| LAD (mm) | 45 (37-45) | 50 (47-53) | <0.01 |
| LVDd (mm) | 49 ± 8.2 | 56 ± 10.3 | 0.15 |
| LVEF (%) | 61 ± 8.4 | 54 ± 11.2 | 0.16 |
| E/e' | 15.3 ± 4.9 | 16.5 ± 5.9 | 0.66 |

Data are given as mean ± SD, median (IQR) or *n* (%). *p* value was determined by two-tailed Fisher's exact test, two-tailed unpaired Student's *t*-test and two-tailed Mann-Whitney U test, as appropriate.

ACE; angiotensin converting enzyme, ARB; angiotensin receptor blocker, ARNI; angiotensin receptor-neprilysin inhibitor, BMI; body mass index, BNP; brain natriuretic peptide, CABG; coronary artery bypass grafting, CRP; C-reactive protein, DOAC; direct oral anticoagulant, eGFR; estimated glomerular filtration rate, Hb; hemoglobin, HbA1c; hemoglobin A1c, HDL; high-density lipoprotein, LAD; left atrial diameter, LDL; low-density lipoprotein, LVDd; left ventricular diastolic diameter, LVEF; left ventricular ejection fraction, SGLT2i; sodium-glucose cotransporter-2 inhibitor, TG; triglycerides.

**Table S4 (related to Figure 3C):** Blood pressure, heart rate, and echocardiographic analysis for parameters of the cardiac function in mice.

| | vehicle | AngII | AngII + VEGFC |
|---|---|---|---|
| SBP (mmHg) | 104 ± 1.80 | 145 ± 2.50**** | 144 ± 6.06**** |
| DBP (mmHg) | 67.3 ± 2.68 | 97.8 ± 3.51*** | 96.2 ± 5.16*** |
| HR (bpm) | 508 ± 6.65 | 506 ± 13.4 | 498 ± 11.3 |
| LVEF (%) | 69.8± 1.94 | 70.0 ± 1.92 | 70.3 ± 1.95 |
| LVPWT (mm) | 0.66 ± 0.02 | 0.93 ±0.04**** | 0.91 ± 0.03**** |
| LVDd (mm) | 3.49 ± 0.09 | 3.51 ± 0.09 | 3.49 ± 0.10 |
| LVDs (mm) | 2.40 ± 0.08 | 2.39 ± 0.09 | 2.40 ± 0.09 |
| E/A | 1.49 ± 0.07 | 1.22 ± 0.07* | 1.22 ± 0.08* |
| E/e' | 24.6 ± 1.91 | 34.7 ± 2.93* | 34.6 ± 3.25* |
| IVRT | 22.8 ± 0.82 | 23.7 ± 1.03 | 23.4 ± 1.01 |
| IVCT | 18.9 ± 0.80 | 19.1 ± 0.68 | 19.1 ± 0.80 |
| LAD (mm) | 2.01 ± 0.04 | 2.03 ± 0.05 | 2.02 ± 0.05 |

Data are mean ± SEM. $n = 12$ *per* group.; * $p < 0.05$, *** p < 0. 001, **** p < 0. 0001, *vs* vehicle by one-way ANOVA followed by the Tukey's *post hoc* test.

DBP; diastolic blood pressure, HR; heart rate, IVCT; isovolumic contraction time, IVRT; isovolumic relaxation time, LAD; left atrial diameter, LVDd; left ventricular diastolic diameter, LVDs; left ventricular systolic diameter, LVEF; left ventricular ejection fraction, LVPWT; left ventricular posterior wall thickness, SBP; systolic blood pressure, VEGFC; vascular endothelial growth factor C.

**Table S5 (related to Figure 5A):** Echocardiographic analysis for parameters of the cardiac function in mice.

| | HFD | HFD+LY3437943 |
|---|---|---|
| LVDd (mm) | 3.39 ± 0.12 | 3.32 ± 0.07 |
| LVDs (mm) | 2.18 ± 0.09 | 2.12 ± 0.05 |
| LVEF (%) | 72.2 ± 0.93 | 72.0 ± 0.90 |
| IVST (mm) | 0.73 ± 0.02 | 0.75 ± 0.02 |
| LVPWT (mm) | 0.98 ± 0.03 | 0.98 ± 0.04 |
| LAD (mm) | 2.18 ± 0.06 | 2.18 ± 0.07 |

Data are mean ± SEM. $n$ = 6 *per* group. $p$ value was determined by two-tailed unpaired Student's $t$-test.

IVST; interventricular septal thickness, LAD; left atrial diameter, LVDd; left ventricular diastolic diameter, LVDs; left ventricular systolic diameter, LVEF; left ventricular ejection fraction, LVPWT; left ventricular posterior wall thickness.

**Table S6 (related to Figure S11A):** Echocardiographic analysis for parameters of the cardiac function in mice.

| | vehicle | AngII |
|---|---|---|
| LVEF (%) | 70.0 ± 2.05 | 70.7 ± 2.01 |
| LVPWT (mm) | 0.66 ± 0.02 | 0.68 ± 0.02 |
| LVDd (mm) | 3.51 ± 0.09 | 3.57 ± 0.11 |
| LVDs (mm) | 2.41 ± 0.09 | 2.40 ± 0.09 |
| E/A | 1.43 ± 0.08 | 1.40 ± 0.08 |
| E/e’ | 25.3 ± 2.02 | 25.9 ± 2.03 |
| IVRT (msec) | 22.5 ± 0.91 | 22.9 ± 0.89 |
| IVCT (msec) | 18.7 ± 0.75 | 19.0 ± 0.76 |
| LAD (mm) | 2.04 ± 0.06 | 2.07 ± 0.06 |

Data are mean ± SEM. $n$ = 12 *per* group. $p$ value was determined by two-tailed unpaired Student’s $t$-test.

IVCT; isovolumic contraction time, IVRT; isovolumic relaxation time, LAD; left atrial diameter, LVDd; left ventricular diastolic diameter, LVDs; left ventricular systolic diameter, LVEF; left ventricular ejection fraction, LVPWT; left ventricular posterior wall thickness.

**Table S7 (related to Figure S11D):** Echocardiographic analysis for parameters of the cardiac function in mice.

| | vehicle | HFD |
|---|---|---|
| LVEF (%) | 70.4 ± 2.06 | 70.9 ± 2.15 |
| LVPWT (mm) | 0.66 ± 0.02 | 0.66 ± 0.02 |
| LVDd (mm) | 3.52 ± 0.08 | 3.55 ± 0.08 |
| LVDs (mm) | 2.43 ± 0.08 | 2.45 ± 0.08 |
| E/A | 1.49 ± 0.09 | 1.46 ± 0.10 |
| E/e’ | 25.8 ± 2.02 | 25.9 ± 2.36 |
| IVRT (msec) | 23.3 ± 0.79 | 23.1 ± 0.81 |
| IVCT (msec) | 18.8 ± 0.87 | 18.4 ± 0.99 |
| LAD (mm) | 2.05 ± 0.06 | 2.06 ± 0.06 |

Data are mean ± SEM. $n$ = 13 *per* group. $p$ value was determined by two-tailed unpaired Student’s $t$-test.

IVCT; isovolumic contraction time, IVRT; isovolumic relaxation time, LAD; left atrial diameter, LVDd; left ventricular diastolic diameter, LVDs; left ventricular systolic diameter, LVEF; left ventricular ejection fraction, LVPWT; left ventricular posterior wall thickness.
