# Supplementary material for: Kynurenic acid mediates epicardial fat-induced lymphatic metabolic dysfunction in atrial fibrillation
Source: Nat Commun. 2026 May 19;17:6616. doi: 10.1038/s41467-026-72974-9 (PMC13381822; doi:10.1038/s41467-026-72974-9)
Supplement: Supplementary file 2 — Reporting summary [file 41467_2026_72974_MOESM2_ESM.pdf]

nature portfolio

Corresponding author(s): Ichitao Abe

Last updated by author(s): Dec 28, 2025

Reporting Summary

Nature Portfolio wishes to improve the reproducibility of the work that we publish. This form provides structure for consistency and transparency in reporting. For further information on Nature Portfolio policies, see our Editorial Policies and the Editorial Policy Checklist.

Statistics

For all statistical analyses, confirm that the following items are present in the figure legend, table legend, main text, or Methods section.

n/a	Confirmed	
☐	☒	The exact sample size (*n*) for each experimental group/condition, given as a discrete number and unit of measurement
☐	☒	A statement on whether measurements were taken from distinct samples or whether the same sample was measured repeatedly
☐	☒	The statistical test(s) used AND whether they are one- or two-sided<br>*Only common tests should be described solely by name; describe more complex techniques in the Methods section.*
☐	☒	A description of all covariates tested
☐	☒	A description of any assumptions or corrections, such as tests of normality and adjustment for multiple comparisons
☐	☒	A full description of the statistical parameters including central tendency (e.g. means) or other basic estimates (e.g. regression coefficient) AND variation (e.g. standard deviation) or associated estimates of uncertainty (e.g. confidence intervals)
☐	☒	For null hypothesis testing, the test statistic (e.g. *F*, *t*, *r*) with confidence intervals, effect sizes, degrees of freedom and *P* value noted<br>*Give P values as exact values whenever suitable.*
☒	☐	For Bayesian analysis, information on the choice of priors and Markov chain Monte Carlo settings
☒	☐	For hierarchical and complex designs, identification of the appropriate level for tests and full reporting of outcomes
☐	☒	Estimates of effect sizes (e.g. Cohen's *d*, Pearson's *r*), indicating how they were calculated

Our web collection on statistics for biologists contains articles on many of the points above.

Software and code

Policy information about availability of computer code

Data collection	PCR: LightCycler 96<br>Seahorse: Wave 2.6<br>LC-TOFMS: MasterHands<br>GC/MS: Smart Metabolites Database Ver. 3<br>single cell RNA-seq: R (v4.4.1), Seurat package (v5.1.0)<br>Electron microscopy: JEM-1200EXII Transmission electron microscope
Data analysis	GraphPad Prism 9.0 (GraphPad Software); SPSS statistical software (version 26.0); Image J software 1.53a

For manuscripts utilizing custom algorithms or software that are central to the research but not yet described in published literature, software must be made available to editors and reviewers. We strongly encourage code deposition in a community repository (e.g. GitHub). See the Nature Portfolio guidelines for submitting code & software for further information.

Data

Policy information about availability of data

All manuscripts must include a data availability statement. This statement should provide the following information, where applicable:

- Accession codes, unique identifiers, or web links for publicly available datasets
- A description of any restrictions on data availability
- For clinical datasets or third party data, please ensure that the statement adheres to our policy

The datasets generated and analyzed during the current study are not publicly available due to patient privacy regulations but are available from the corresponding author on reasonable request and with appropriate institutional review board approval. Clinical data cannot be shared due to ethical restrictions regarding patient confidentiality.

Research involving human participants, their data, or biological material

Policy information about studies with human participants or human data. See also policy information about sex, gender (identity/presentation), and sexual orientation and race, ethnicity and racism.

Reporting on sex and gender	Sex (biological attribute) was determined based on medical records and administrative data. Gender identity was not specifically assessed in this study design. Sex-disaggregated analyses were not performed due to insufficient sample size. The primary focus of this study was on tissue-level molecular mechanisms rather than sex-based differences in cardiovascular outcomes. Individual-level sex data consent was obtained as part of the informed consent process, and sex distribution is reported in the characteristics.
Reporting on race, ethnicity, or other socially relevant groupings	All study participants were Japanese individuals of Asian ethnicity, reflecting the patient population served by Oita University Hospital. Given the homogeneous ethnic composition of the study population, race and ethnicity were not used as analytical variables.
Population characteristics	The study population consisted of adult patients with cardiovascular disease (valvular disease, coronary artery disease, or aortic disease) requiring elective open-heart surgery at Oita University Hospital. Detailed demographic characteristics including age, comorbidities, surgical risk factors, and cardiovascular disease categories are provided in the Table.
Recruitment	Patients were consecutively recruited from those scheduled for cardiac surgery at the Department of Cardiovascular Surgery, Oita University Hospital. Eligible participants included adult patients (≥18 years) undergoing elective open-heart surgery for cardiovascular disease. Inclusion criteria were: (1) valvular disease requiring surgical intervention, (2) coronary artery disease requiring surgical revascularization, or (3) aortic disease requiring surgical repair. Exclusion criteria was patients deemed inappropriate by the research investigators based on clinical judgment. Patients with conditions that could be adversely affected by additional blood sampling (such as severe anemia) were excluded from participation. All eligible patients were approached for participation and provided detailed written and verbal explanations of the study procedures. Written informed consent was obtained from all participants prior to enrollment, following approval by the Oita University Faculty of Medicine Ethics Committee. No incentives or compensation were provided for participation. The recruitment strategy aimed to minimize selection bias through consecutive enrollment of all eligible patients during the study period.
Ethics oversight	The study protocol was approved by the Ethics Committee of Oita University Hospital (approval number: 794 and 1797) and was conducted in accordance with the tenets of the Declaration of Helsinki. This study was registered in the University Hospital Medical Information Network Clinical Trials Registry (UMIN000042229). Left atrial appendage (LAA), subcutaneous adipose tissue (SAT) and epicardial adipose tissue (EAT) were collected under the Oita University Committee on Clinical Investigations. Potential subjects were recruited from the operating room rosters of Oita University Hospital and Oita Oka Hospital. Subjects undergoing elective open-heart surgery were included and provided written informed consent preoperatively.

Note that full information on the approval of the study protocol must also be provided in the manuscript.

Field-specific reporting

Please select the one below that is the best fit for your research. If you are not sure, read the appropriate sections before making your selection.

☒ Life sciences ☐ Behavioural & social sciences ☐ Ecological, evolutionary & environmental sciences

For a reference copy of the document with all sections, see nature.com/documents/nr-reporting-summary-flat.pdf

Life sciences study design

All studies must disclose on these points even when the disclosure is negative.

Sample size	Sample size was defined by the retrieved studies based on our literature search (PMID: 14581396., PMID: 26239655., PMID: 23525094., PMID: 28096344). Thus, sample size calculation was not carried out.
Data exclusions	No data were excluded from analyses after enrollment.

Replication	As human data is a clinical observational study, with limited human sample collection, reproducibility of our findings were based on the use of different technical approaches to confirm the initial findings. In vivo and In vitro assays were performed with biological replicates in two or three experimental repeats.
Randomization	No randomization was applied as was not applicable to the human study. Mice were randomly assigned at the time of purchase or weaning to minimize any potential bias.
Blinding	Patients samples were labeled with nonidentifying numbers and sample analysis was performed blindly by two investigators.<br>All mass spectrometry analysis were performed by technical staffs at the Oita University core who were blinded to the experimental groups.<br>Mice and in vitro analyses were performed by the authors who were blinded to the experimental groups.

Behavioural & social sciences study design

All studies must disclose on these points even when the disclosure is negative.

Study description	*Briefly describe the study type including whether data are quantitative, qualitative, or mixed-methods (e.g. qualitative cross-sectional, quantitative experimental, mixed-methods case study).*
Research sample	*State the research sample (e.g. Harvard university undergraduates, villagers in rural India) and provide relevant demographic information (e.g. age, sex) and indicate whether the sample is representative. Provide a rationale for the study sample chosen. For studies involving existing datasets, please describe the dataset and source.*
Sampling strategy	*Describe the sampling procedure (e.g. random, snowball, stratified, convenience). Describe the statistical methods that were used to predetermine sample size OR if no sample-size calculation was performed, describe how sample sizes were chosen and provide a rationale for why these sample sizes are sufficient. For qualitative data, please indicate whether data saturation was considered, and what criteria were used to decide that no further sampling was needed.*
Data collection	*Provide details about the data collection procedure, including the instruments or devices used to record the data (e.g. pen and paper, computer, eye tracker, video or audio equipment) whether anyone was present besides the participant(s) and the researcher, and whether the researcher was blind to experimental condition and/or the study hypothesis during data collection.*
Timing	*Indicate the start and stop dates of data collection. If there is a gap between collection periods, state the dates for each sample cohort.*
Data exclusions	*If no data were excluded from the analyses, state so OR if data were excluded, provide the exact number of exclusions and the rationale behind them, indicating whether exclusion criteria were pre-established.*
Non-participation	*State how many participants dropped out/declined participation and the reason(s) given OR provide response rate OR state that no participants dropped out/declined participation.*
Randomization	*If participants were not allocated into experimental groups, state so OR describe how participants were allocated to groups, and if allocation was not random, describe how covariates were controlled.*

Ecological, evolutionary & environmental sciences study design

All studies must disclose on these points even when the disclosure is negative.

Study description	*Briefly describe the study. For quantitative data include treatment factors and interactions, design structure (e.g. factorial, nested, hierarchical), nature and number of experimental units and replicates.*
Research sample	*Describe the research sample (e.g. a group of tagged Passer domesticus, all Stenocereus thurberi within Organ Pipe Cactus National Monument), and provide a rationale for the sample choice. When relevant, describe the organism taxa, source, sex, age range and any manipulations. State what population the sample is meant to represent when applicable. For studies involving existing datasets, describe the data and its source.*
Sampling strategy	*Note the sampling procedure. Describe the statistical methods that were used to predetermine sample size OR if no sample-size calculation was performed, describe how sample sizes were chosen and provide a rationale for why these sample sizes are sufficient.*
Data collection	*Describe the data collection procedure, including who recorded the data and how.*
Timing and spatial scale	*Indicate the start and stop dates of data collection, noting the frequency and periodicity of sampling and providing a rationale for these choices. If there is a gap between collection periods, state the dates for each sample cohort. Specify the spatial scale from which the data are taken*
Data exclusions	*If no data were excluded from the analyses, state so OR if data were excluded, describe the exclusions and the rationale behind them, indicating whether exclusion criteria were pre-established.*
Reproducibility	*Describe the measures taken to verify the reproducibility of experimental findings. For each experiment, note whether any attempts to repeat the experiment failed OR state that all attempts to repeat the experiment were successful.*

Randomization | *Describe how samples/organisms/participants were allocated into groups. If allocation was not random, describe how covariates were controlled. If this is not relevant to your study, explain why.*

Blinding | *Describe the extent of blinding used during data acquisition and analysis. If blinding was not possible, describe why OR explain why blinding was not relevant to your study.*

Did the study involve field work? ☐ Yes ☐ No

Field work, collection and transport

Field conditions | *Describe the study conditions for field work, providing relevant parameters (e.g. temperature, rainfall).*

Location | *State the location of the sampling or experiment, providing relevant parameters (e.g. latitude and longitude, elevation, water depth).*

Access & import/export | *Describe the efforts you have made to access habitats and to collect and import/export your samples in a responsible manner and in compliance with local, national and international laws, noting any permits that were obtained (give the name of the issuing authority, the date of issue, and any identifying information).*

Disturbance | *Describe any disturbance caused by the study and how it was minimized.*

Reporting for specific materials, systems and methods

We require information from authors about some types of materials, experimental systems and methods used in many studies. Here, indicate whether each material, system or method listed is relevant to your study. If you are not sure if a list item applies to your research, read the appropriate section before selecting a response.

Materials & experimental systems

n/a	Involved in the study	
☐	☒	Antibodies
☐	☒	Eukaryotic cell lines
☒	☐	Palaeontology and archaeology
☐	☒	Animals and other organisms
☐	☒	Clinical data
☒	☐	Dual use research of concern
☒	☐	Plants

Methods

n/a	Involved in the study	
☒	☐	ChIP-seq
☐	☒	Flow cytometry
☒	☐	MRI-based neuroimaging

Antibodies

Antibodies used | The following antibodies were used in this study: anti LYVE-1 antibody (1:300 dilution in immunoprecipitation. Catalog no. AF2089 R&D Systems), anti LYVE-1 antibody (1:100 dilution in immunoprecipitation, Catalog no. PA1-16635 Invitrogen), anti PROX1 antibody (1:250 dilution in immunoblotting, and 5µg/mL in immunoprecipitation, Catalog no. AF2727 R&D Systems), anti TAGLN antibody (1:500 in immuno blotting, and 1:100 dilution in immunoprecipitation,Catalog no. #40471 Cell Signaling), anti F4/80 antibody (1:100 dilution in immunoprecipitation, Catalog no. ab16911 Abcam), anti β-actin (1:1000 dilution in immunoblotting, Catalog no. #4967 Cell Signaling Technology), anti NLRP3 (1:250 dilution in immunoblotting, Catalog no. NBP2-12446 Novus Biologicals), anti ASC (1:250 dilution in immunoblotting, Catalog no. SC-514414 Santa Cruz), anti Caspase1 (1:500 dilution in immunoblotting, Catalog no. SC-56036 Santa Cruz), anti IL-1β (1:500 dilution in immunoblotting, Catalog no. ab9722 Abcam), anti Malondialdehyde (1:500 dilution in immunoblotting, Catalog no. ab27642 Abcam), anti oxidized-CaMKII (Met281/282) (1:1000 dilution in immunoblotting, Catalog no. 07-1387 Sigma-Aldrich), anti CaMKII (1:1000 dilution in immunoblotting, Catalog no. ab52476 Abcam), anti p-RyR2 (Ser2814) (1:250 dilution in immunoblotting, Catalog no. A010-31AP Badrilla), anti RyR2 (1:500 dilution in immunoblotting, Catalog no. PA5-104444 Thermo Fisher), anti α/β-Tubulin (1:1000 dilution in immunoblotting, Catalog no. #2148 Cell Signaling Technology), anti p-SMAD2 (Ser465/467)/SMAD3 (Ser423/425) (1:500 dilution in immunoblotting, Catalog no. #8828 Cell Signaling Technology), anti SMAD2/3 (1:1000 dilution in immunoblotting, Catalog no. #8685 Cell Signaling Technology), anti SMAD4 (1:200 dilution in immunoblotting, Catalog no. SC-7966 Santa Cruz), anti p-TAK1 (Thr184/187) (1:500 dilution in immunoblotting, Catalog no. #4508 Cell Signaling Technology), anti TAK1 (1:500 dilution in immunoblotting, Catalog no. #5206 Cell Signaling Technology), anti p-Erk1/2 (1:500 dilution in immunoblotting, Catalog no. #9101 Cell Signaling Technology), anti Erk1/2 (1:1000 dilution in immunoblotting, Catalog no. #4695 Cell Signaling Technology), anti p-p38MAPK (1:1000 dilution in immunoblotting, Catalog no. #4511 Cell Signaling Technology), anti p38MAPK (1:1000 dilution in immunoblotting, Catalog no. #8690 Cell Signaling Technology), rabbit anti GPR35 antibody (1:100 Catalog no. 55248-1-AP Proteintech)anti Podoplanin-PE (1:80, Catalog no.127407, Biolegend), and anti CD31-APC (1:300, Catalog no.160209, Biolegend).

Validation | Antibodies were validated based on the size of band in western blotting (molecular weight), specificity/selectivity assessed by using samples from humans/mice/rats/cells, and reproducibility of the results. All antibodies were purchased directly from a commercial supplier. Validation data can be found on the manufacture's website.
anti LYVE-1 (AF2089): Fig.1H

anti LYVE-1 (PA1-16635):Fig.1P, 3F, 4C
anti PROX1 : Supplemental Fig. 3H, 3J, 4D, 4F
anti TAGLN : Supplemental Fig. 3H, 3J, 4D, 4F
anti F4/80 : Supplemental Fig. 7C
anti β-actin : Supplemental Fig. 1A, 7F, 7H, 8B, 8H
anti NLRP3 : Supplemental Fig. 1A, 7H, 8B, 8H
anti ASC : Supplemental Fig. 1A, 7H, 8B, 8H
anti Caspase1 : Supplemental Fig. 1A, 7H, 8B, 8H
anti IL-1β : Supplemental Fig. 1A, 7H, 8B, 8H
anti Malondialdehyde : Supplemental Fig. 7F
anti oxidized-CaMKII : Supplemental Fig. 6D, 7F
anti CaMKII : Supplemental Fig.6D, 7F
anti p-RyR2 (Ser2814) : Supplemental Fig.6D, 7F
anti RyR2 : Supplemental Fig.6D, 7F
anti α/β-Tubulin : Supplemental Fig. 3J, 4F, 4H
anti p-SMAD2 (Ser465/467)/SMAD3 (Ser423/425) : Supplemental Fig. 3H
anti SMAD2/SMAD3 : Supplemental Fig. 3H
anti SMAD4 : Supplemental Fig. 3H
anti p-TAK1 (Thr184/187): Supplemental Fig. 3H
anti TAK1 : Supplemental Fig. 3H
anti p-Erk1/2 : Supplemental Fig. 3H
anti Erk1/2 : Supplemental Fig. 3H
anti p-p38MAPK : Supplemental Fig. 3H
anti GPR35 antibody: Supplemental Fig. 5B
anti p38MAPK: Supplemental Fig. 3H
anti Podoplanin-PE: Fig.3H, Supplemental Fig. 6E
anti CD31-APC: Fig.3H, Supplemental Fig. 6E
anti LYVE-1 (AF2089): https://www.rndsystems.com/products/human-lyve-1-biotinylated-antibody_baf2089
anti LYVE-1 (PA1-16635): https://www.thermofisher.com/antibody/product/LYVE1-Antibody-Polyclonal/PA1-16635
anti PROX1 : https://www.rndsystems.com/products/human-prox1-antibody_af2727
anti TAGLN : https://www.cellsignal.com/products/primary-antibodies/transgelin-tagln-antibody/40471
anti F4/80 : https://www.abcam.com/en-us/products/primary-antibodies/f4-80-antibody-bm8-ab16911
anti β-actin : https://www.cellsignal.com/products/primary-antibodies/b-actin-antibody/4967
anti NLRP3 : https://www.novusbio.com/products/nlrp3-nalp3-antibody_nbp2-12446
anti ASC : https://www.scbt.com/p/asc-antibody-b-3anti Caspase1 : https://www.scbt.com/p/caspase-1-antibody-14f468
anti IL-1β : https://www.abcam.co.jp/products/primary-antibodies/il-1-beta-antibody-ab9722.html
anti Malondialdehyde : https://www.abcam.co.jp/products/primary-antibodies/malondialdehyde-antibody-ab27642.html
anti oxidized-CaMKII : https://www.merckmillipore.com/JP/ja/product/Anti-oxidized-CaM-Kinase-II-Met281-282-Antibody,MM_NF-07-1387
anti CaMKII : https://www.abcam.co.jp/products/primary-antibodies/camkii-antibody-ep1829y-ab52476.html
anti p-RyR2 (Ser2814) : https://badrilla.com/product/ryanodine-receptor-2-ryr2-pser2814-pab/
anti RyR2 : https://www.thermofisher.com/antibody/product/RyR2-Antibody-Polyclonal/PA5-104444
anti α/β-Tubulin : https://www.cellsignal.com/products/primary-antibodies/a-b-tubulin-antibody/2148
anti p-SMAD2 (Ser465/467)/SMAD3 (Ser423/425) : https://www.cellsignal.com/products/primary-antibodies/phospho-smad2-ser465-467-smad3-ser423-425-d27f4-rabbit-mab/8828
anti SMAD2/SMAD3 : https://www.cellsignal.com/products/primary-antibodies/smad2-3-d7g7-xp-rabbit-mab/8685
anti SMAD4 : https://www.scbt.com/ja/p/smad4-antibody-b-8
anti p-TAK1 (Thr184/187): https://www.cellsignal.com/products/primary-antibodies/phospho-tak1-thr184-187-90c7-rabbit-mab/4508anti TAK1 : https://www.cellsignal.com/products/primary-antibodies/tak1-d94d7-rabbit-mab/5206
anti p-Erk1/2 : https://www.cellsignal.com/products/primary-antibodies/phospho-p44-42-mapk-erk1-2-thr202-tyr204-antibody/9101
anti Erk1/2 : https://www.cellsignal.com/products/primary-antibodies/p44-42-mapk-erk1-2-137f5-rabbit-mab/4695
anti p-p38MAPK : https://www.cellsignal.com/products/primary-antibodies/phospho-p38-mapk-thr180-tyr182-d3f9-xp-rabbit-mab/4511
anti p38MAPK: https://www.cellsignal.com/products/primary-antibodies/p38-mapk-d13e1-xp-rabbit-mab/8690
anti GPR35 antibody: https://www.ptglab.co.jp/Products/GPR35-Antibody-55248-1-AP.htm
anti Podoplanin-PE: https://www.biolegend.com/ja-jp/products/pe-anti-mouse-podoplanin-antibody-4882
anti CD31-APC: https://www.biolegend.com/ja-jp/products/apc-anti-mouse-cd31-pecam-1-21557

Eukaryotic cell lines

Policy information about cell lines and Sex and Gender in Research

Cell line source(s)	Human Lymphatic Endothelial Cells: adult donor
Authentication	The cells are tested for cell morphology and cell-type specific markers, e.g. CD31 and Podoplanin, using flow cytometric analyses.
Mycoplasma contamination	Cell lines were tested for mycoplasma contamination monthly
Commonly misidentified lines (See ICLAC register)	N/A

Palaeontology and Archaeology

Specimen provenance	*Provide provenance information for specimens and describe permits that were obtained for the work (including the name of the issuing authority, the date of issue, and any identifying information). Permits should encompass collection and, where applicable, export.*
Specimen deposition	*Indicate where the specimens have been deposited to permit free access by other researchers.*
Dating methods	*If new dates are provided, describe how they were obtained (e.g. collection, storage, sample pretreatment and measurement), where they were obtained (i.e. lab name), the calibration program and the protocol for quality assurance OR state that no new dates are provided.*

☐ Tick this box to confirm that the raw and calibrated dates are available in the paper or in Supplementary Information.

Ethics oversight	*Identify the organization(s) that approved or provided guidance on the study protocol, OR state that no ethical approval or guidance was required and explain why not.*

Note that full information on the approval of the study protocol must also be provided in the manuscript.

Animals and other research organisms

Policy information about studies involving animals; ARRIVE guidelines recommended for reporting animal research, and Sex and Gender in Research

Laboratory animals	C57BL/6J wild-type mice and Sprague-Dawley rats were obtained from KBT Oriental at 8 weeks of age and were kept in an animal room, one animal in each cage, with a chow and water at ambient temperature (22°C) under a 12 hr:12 hr light-dark cycle.
Wild animals	This study did not involve wild animals.
Reporting on sex	For mouse experiments, only males are reported. Male mice were used based on the fact that the development of metabolic disease in response to diet-induced-obesity develops faster and male mice develop a stronger phenotype.
Field-collected samples	No field collected samples.
Ethics oversight	All animal experiments were performed according to procedures approved by the guidelines of the Oita University Animal Ethics Committee, Japan, for the care and use of laboratory animals, which follow the guidelines established by the US National Institutes of Health.

Note that full information on the approval of the study protocol must also be provided in the manuscript.

Clinical data

Policy information about clinical studies

All manuscripts should comply with the ICMJE guidelines for publication of clinical research and a completed CONSORT checklist must be included with all submissions.

Clinical trial registration	UMIN000042229
Study protocol	The study protocol is provided with the publication.
Data collection	Seahorse assays were performed at the National Cerebral and Cardiovascular Center, Osaka, Japan, in 2025.<br>3D spheroid assays, migration assays, and EdU assays were performed at The University of Tokyo, Tokyo, Japan, in 2025.<br>All other data, including human source data, were collected at Oita University, Oita, Japan, between 2019 and 2025.
Outcomes	Primary outcomes were lymphangiogenic, fibrotic, and inflammatory condition in epicardial adipose tissue.<br>Secondary outcomes were metabolic function in atrium.

Dual use research of concern

Policy information about dual use research of concern

Hazards

Could the accidental, deliberate or reckless misuse of agents or technologies generated in the work, or the application of information presented in the manuscript, pose a threat to:

No | Yes

- [ ] [ ] Public health
- [ ] [ ] National security
- [ ] [ ] Crops and/or livestock
- [ ] [ ] Ecosystems
- [ ] [ ] Any other significant area

Experiments of concern

Does the work involve any of these experiments of concern:

No | Yes

- [ ] [ ] Demonstrate how to render a vaccine ineffective
- [ ] [ ] Confer resistance to therapeutically useful antibiotics or antiviral agents
- [ ] [ ] Enhance the virulence of a pathogen or render a nonpathogen virulent
- [ ] [ ] Increase transmissibility of a pathogen
- [ ] [ ] Alter the host range of a pathogen
- [ ] [ ] Enable evasion of diagnostic/detection modalities
- [ ] [ ] Enable the weaponization of a biological agent or toxin
- [ ] [ ] Any other potentially harmful combination of experiments and agents

Plants

Seed stocks: Not Applicable

Novel plant genotypes: Not Applicable

Authentication: Not Applicable

ChIP-seq

Data deposition

- [ ] Confirm that both raw and final processed data have been deposited in a public database such as GEO.
- [ ] Confirm that you have deposited or provided access to graph files (e.g. BED files) for the called peaks.

Data access links
May remain private before publication.: *For "Initial submission" or "Revised version" documents, provide reviewer access links. For your "Final submission" document, provide a link to the deposited data.*

Files in database submission: *Provide a list of all files available in the database submission.*

Genome browser session
(e.g. UCSC): *Provide a link to an anonymized genome browser session for "Initial submission" and "Revised version" documents only, to enable peer review. Write "no longer applicable" for "Final submission" documents.*

Methodology

Replicates: *Describe the experimental replicates, specifying number, type and replicate agreement.*

Sequencing depth: *Describe the sequencing depth for each experiment, providing the total number of reads, uniquely mapped reads, length of reads and whether they were paired- or single-end.*

Antibodies: *Describe the antibodies used for the ChIP-seq experiments; as applicable, provide supplier name, catalog number, clone name, and lot number.*

Peak calling parameters: *Specify the command line program and parameters used for read mapping and peak calling, including the ChIP, control and index files used.*

Data quality	*Describe the methods used to ensure data quality in full detail, including how many peaks are at FDR 5% and above 5-fold enrichment.*
Software	*Describe the software used to collect and analyze the ChIP-seq data. For custom code that has been deposited into a community repository, provide accession details.*

Flow Cytometry

Plots

Confirm that:

☒ The axis labels state the marker and fluorochrome used (e.g. CD4-FITC).

☒ The axis scales are clearly visible. Include numbers along axes only for bottom left plot of group (a 'group' is an analysis of identical markers).

☒ All plots are contour plots with outliers or pseudocolor plots.

☒ A numerical value for number of cells or percentage (with statistics) is provided.

Methodology

Sample preparation	Lymphatic endothelial cells isolation and sorting: Mouse atria were digested by collagenase D (1.5 U ml-1) and dispase II (2.5 U ml-1). MACS CD45 Micro beads for mice (1:10, Catalog no. 130-052-301, Miltenyi Biotec) and MACS LS columns (Miltenyi Biotec) were used to deplete lineage+ (Lin+) cells. The following antibodies were used for the isolation of mouse atrial lymphatic endothelial cells (Lin-: CD31+: Podoplanin+): Podoplanin-PE (1:80, Catalog no.127407, Biolegend) and CD31-APC (1:300, Catalog no.160209, Biolegend) in autoMACS Rising Solution (Miltenyi Biotec) containing 0.5% BSA in the dark at 4 °C for 15 minutes.<br>Cell proliferation assays: LECs were treated with No AFib or AFib-EAT conditioned media, with vehicle or kynurenic acid for 2 days, then incubated with 10 µM EdU (Click-iT Plus EdU Flow Cytometry Assay Kit, Thermo Fisher Scientific) for 2 h in serum-free medium. Subsequently, cells were fixed and processed according to the manufacturer's instructions.
Instrument	FACS Aria II equipped with 100 mm nozzle diameter, CytoFLEX, and FACS Melody
Software	FlowJo software (version 10.8.1)
Cell population abundance	Atrial lymphatic endothelial cells (Lin-: Cd31+: Pdpn+ cells) represent approximately 4-8% of the endothelial fraction of the atrium. EdU-positive LECs represent approximately 5-18% of the cultured LECs.
Gating strategy	For atrial lymphatic endothelial cells sorting, cells were first gated on FSC, SSC and singlets, then Cd31+, Pdpn+ cells.<br>For EdU assay: singlet cells are gated initially on FSC, SSC, then EdU+ cells.

☒ Tick this box to confirm that a figure exemplifying the gating strategy is provided in the Supplementary Information.

Magnetic resonance imaging

Experimental design

Design type	*Indicate task or resting state; event-related or block design.*
Design specifications	*Specify the number of blocks, trials or experimental units per session and/or subject, and specify the length of each trial or block (if trials are blocked) and interval between trials.*
Behavioral performance measures	*State number and/or type of variables recorded (e.g. correct button press, response time) and what statistics were used to establish that the subjects were performing the task as expected (e.g. mean, range, and/or standard deviation across subjects).*

Acquisition

Imaging type(s)	*Specify: functional, structural, diffusion, perfusion.*
Field strength	*Specify in Tesla*
Sequence & imaging parameters	*Specify the pulse sequence type (gradient echo, spin echo, etc.), imaging type (EPI, spiral, etc.), field of view, matrix size, slice thickness, orientation and TE/TR/flip angle.*
Area of acquisition	*State whether a whole brain scan was used OR define the area of acquisition, describing how the region was determined.*

Diffusion MRI ☐ Used ☐ Not used

Preprocessing

Preprocessing software	*Provide detail on software version and revision number and on specific parameters (model/functions, brain extraction, segmentation, smoothing kernel size, etc.).*
Normalization	*If data were normalized/standardized, describe the approach(es): specify linear or non-linear and define image types used for transformation OR indicate that data were not normalized and explain rationale for lack of normalization.*
Normalization template	*Describe the template used for normalization/transformation, specifying subject space or group standardized space (e.g. original Talairach, MNI305, ICBM152) OR indicate that the data were not normalized.*
Noise and artifact removal	*Describe your procedure(s) for artifact and structured noise removal, specifying motion parameters, tissue signals and physiological signals (heart rate, respiration).*
Volume censoring	*Define your software and/or method and criteria for volume censoring, and state the extent of such censoring.*

Statistical modeling & inference

Model type and settings	*Specify type (mass univariate, multivariate, RSA, predictive, etc.) and describe essential details of the model at the first and second levels (e.g. fixed, random or mixed effects; drift or auto-correlation).*
Effect(s) tested	*Define precise effect in terms of the task or stimulus conditions instead of psychological concepts and indicate whether ANOVA or factorial designs were used.*

Specify type of analysis: ☐ Whole brain ☐ ROI-based ☐ Both

Statistic type for inference<br>(See Eklund et al. 2016)	*Specify voxel-wise or cluster-wise and report all relevant parameters for cluster-wise methods.*
Correction	*Describe the type of correction and how it is obtained for multiple comparisons (e.g. FWE, FDR, permutation or Monte Carlo).*

Models & analysis

n/a	Involved in the study
☐	☐ Functional and/or effective connectivity
☐	☐ Graph analysis
☐	☐ Multivariate modeling or predictive analysis

Functional and/or effective connectivity	*Report the measures of dependence used and the model details (e.g. Pearson correlation, partial correlation, mutual information).*
Graph analysis	*Report the dependent variable and connectivity measure, specifying weighted graph or binarized graph, subject- or group-level, and the global and/or node summaries used (e.g. clustering coefficient, efficiency, etc.).*
Multivariate modeling and predictive analysis	*Specify independent variables, features extraction and dimension reduction, model, training and evaluation metrics.*
