# Supplementary material for: Kynurenic acid mediates epicardial fat-induced lymphatic metabolic dysfunction in atrial fibrillation
Source: Nat Commun. 2026 May 19;17:6616. doi: 10.1038/s41467-026-72974-9 (PMC13381822; doi:10.1038/s41467-026-72974-9)
Supplement: Supplementary file 3 — Transparent Peer Review file [file 41467_2026_72974_MOESM3_ESM.pdf]

nature portfolio

Peer Review File

Kynurenic Acid Mediates Epicardial Fat-induced Lymphatic Metabolic Dysfunction in Atrial Fibrillation

Corresponding Author: Dr Ichitaro Abe

This file contains all reviewer reports in order by version, followed by all author rebuttals in order by version.

Version 0:

Reviewer comments:

Reviewer #1

(Remarks to the Author)
Interesting study providing novel and clinically and theraputically relevant insights into AF pathophysiology.
Specific comments:
- Patients cohorts, table S1: AF causes atrial remodeling. The time from last documented pAF episodes should be added to the pAF group of patients. Both AF groups are dominated by valve disease patients, whereas the control groups consists mostly of CAD patients. Thus, the reported changes in lymphangionesis could be due to valve disease instead of AF per se. In addition, perAF patients had lower eGFR pointing to presence of CKD. The latter is known to cause AF by activation of the NLRP3 inflammasome and thus inflammation (PMID: 37581942). Again, the changes in lymphangionesis could be a result of activation of he NLRP3 system due to CKD in LA of these patients. The perAF patients have also larger LA. The authors should also perform two-way ANOVA to see whether there is an interaction effect between LA size and AF and eGFR and AF on lymphangionesis.
- Patients cohorts, tables S2,3: the patient characteristcs are imcomplete (comorbidities, etc, analog to table S1) and similar considerations apply as above. The type of AF was not specified.
- Fig. 1: Do patients with AF have a stronger activation of the NLRP3 inflammasome, as previously reported in RA of pAF, perAF patients (PMID: 29802206) and in aptients prone to postoperative AF (PMID: 32762493).
- AngII-induced AF model: The authors show that lymphangiogenesis is reduced, similarly to AF patients. It would be importnat to show whether the model recapitulates published alterations of the atria (PMID: 24030498): increased ROS generation, upregulated oxidized CaMKII, abnormal Ca2+-handling promoting triggered activity, etc. It is also key to check whether the changes in lymphangiogenesis precede the changes in CaMKII signaling and its consequences. This is particularly important, because VEGFC rescued the proarrhythmic phenotype, pointing to lymphangiogenesis as being potentially the primary effect. However, it is possible that VEGFC improves the phenotype by acting on CaMKII and Ca2+-handling.
- AngII-induced AF model: These mice have diastolic dysfunction pointing to a potential feedback of the ventricles on atrial function. The authors argue that lymphangiogenesis appears more important in the atria, but they ignore that other aspects of ventricular dysfuncton(ROS production, hemodynamic consequences, etc.) could determine the atrial phenotype. In addition, AngII activates the NLRP3 system in the ventricles as well (reviewed in PMID: 31441689).
- Obesity-induced AF model: Previous papers (PMID: 32654503; PMID: 33523143) showed that obesity in mice cause atrial electrical, structural and Ca2+-handling remodeling that promote AF. One key mechanism appears to be upregulation of the NLRP3 inflammasome system. Since genetic deletion of NLRP3 prevents the proarrhythmic substrate in obese mice, this could be interpreted as a potential mechanism upstream of impaired lymphangiogenesis. The authors claim that inflammatory remodeling does not contribute, but a comprehensive study of inflammatory signaling pathways and their time course were not demonstrated. Again, the effect of weight reduction on lymphangiogenesis and AF in the present obeity model could be a consequence of reduced inflammatory burden, with lymphangiogenesis being a bystander only. This reviewer did not find info on ventricular function in the obesity model.

Reviewer #2

(Remarks to the Author)
Manuscript ID: NCOMMS-24-78617-T by Masaki Takahashiet al.
Manuscript Title: Kynurenic Acid Mediates Epicardial Fat-induced Lymphatic Metabolic Dysfunction in Atrial Fibrillation

General: In this manuscript, authors identify defective atrial lymphangiogenesis as a crucial player in AFib pathogenesis. By using human samples and a broad in vivo, ex vivo and in vitro experimental battery, authors show that epicardial adipose tissue (EAT) from AFib patients generates kynurenic acid that impacts lymphatic endothelial cell metabolism and mitochondrial homeostasis through GPR35, finally inducing endothelial-to-mesenchymal transition. They also demonstrate how VEGFC treatment reduces AFib inducibility.

In general, this work is relevant in the heart and cardiac lymphatics fields and could have important/potential applications, but some clarifications and additional information should be provided by authors.

Major comments: Although the manuscript presents interesting data, this reviewer propose adding some clarifications and performing additional experiments to complete or add value to the results included in the manuscript in its current form:

1. Although the effect of kynurenic acid in the impaired lymphangiogenesis in AFib pathogenesis was not known yet, the role of lymphatic vessels in the pathophysiology of AFib has been already published by the same authors, fact that reduces the novelty of the present work. In a recent manuscript published in European Heart Journal in 2023 (Role of atrial lymphatics in atrial myocardial fibrosis and atrial fibrillation: a human and mouse study; M Takahashi, I Abe, H Yamasaki, T Harada, M Takano, H Kondo, Y Teshima, S Miyamoto, N Takahashi, European Heart Journal, Volume 44, Issue Supplement_2, ehad655.3171, https://doi.org/10.1093/eurheartj/ehad655.3171), the same authors describe the importance of lymphatic vessels in AFib. In that work, authors analysed human LAA samples from PAF and PeAF patients and showed that mRNA expression of lymphangiogenesis-related genes (LYVE1, PROX1, and VEGFR3) was significantly lower in the PeAF group compared to the PAF group. Moreover, they showed that the number of LYVE1-positive atrial lymphatic endothelial cells (LECs) was also lower in the PeAF group, and there was increased mRNA expression of fibrosis-related genes (Tgfb, Col1a1, and Col3a1). In mice, they use the same models used in this manuscript (transesophageal burst pacing and burst pacing in isolated perfused hearts using a Langendorff apparatus) and performed similar assays injecting mice with vehicle or AngII, and also, treating them with VEGFC. They saw that VEGFC treatment attenuated AngII-induced enhancement of vulnerability to AF in in vivo experiments.

Despite the existence of the mentioned results published in https://doi.org/10.1093/eurheartj/ehad655.3171, which are very closed to the data presented here, there is no reference to that manuscript in the present work.

Moreover, in the Abstract, authors mention “Here, we identify impaired atrial lymphangiogenesis as a critical determinant in AFib pathogenesis. Analysis of human left atrial appendage specimens reveals decreased lymphatic vessel density in AFib patients compared to those in sinus rhythm.” This sentence is not fully correct as this characterization was already done in a previous work of the same authors (already published). Also, in the Abstract, authors mention “In vivo studies utilizing angiotensin II-induced and high-fat diet mouse models confirm the critical role of lymphatic dysfunction in AFib susceptibility. Therapeutic interventions promoting lymphangiogenesis, either through VEGFC administration or weight loss intervention by LY3437943 (the novel triple GIP, GLP-1, and glucagon receptor agonist), significantly attenuate AFib inducibility. These findings establish lymphatic dysfunction as a novel pathogenic mechanism in AFib and highlight lymphatic vessel formation as a promising therapeutic target.” This sentence is not fully correct as these in vivo experiments (or part of them) were already performed in a previous authors´work (already published).

In the Introduction and Discussion sections, authors do not refer to the original manuscript published in European Heart Journal https://doi.org/10.1093/eurheartj/ehad655.3171), in which they identified the role of defective atrial lymphangiogenesis in AFib pathogenesis.

It is not clear for this reviewer which is the novel data related to this work in comparison with the manuscript already published. Please clarify.

2. In order to study the effect of conditioned media in the functional impairment of LECs, authors measured paracellular permeability and tube formation capacity. However, during the lymphangiogenic process, LECs also proliferate, migrate and invade the extracellular matrix, finally forming a new sprout and a nascent vessel. It would be great to evaluate the proliferation rate, migration and invasion properties and sprouting capacity of LECs by performing EdU assays, Boyden chamber assays, spheroid assays, etc. in presence of No AFib- or AFib-EAT conditioned media.

3. Although authors used liquid chromatography time-of-flight mass spectrometry to conduct a comprehensive metabolite profiling of No AFib-EAT and AFib-EAT conditioned media from human samples and identify the metabolite kynurenic acid, they do not measure the content of this metabolite in the in vivo models. It should be interesting to perform the same analysis in vivo to corroborate the data obtained by RTPCR about increased Ido1 expression and decreased Kat1 and Kat2 expression.

4. Authors claim that LECs in AFib had compromised mitochondrial homeostasis, evidenced by shortened and swollen mitochondria with reduced cristae density after performing transmission electron microscopy. This reviewer suggests performing additional tests to more deeply evaluate mitochondrial homeostasis, such as mitochondrial respiration by Seahorse, mitochondrial membrane potential, mitochondrial ROS, etc..

5. Authors claim that LECs in AFib undergo Endothelial-to-Mesenchymal Transition (EndoMT). However, they only analysed the expression of lymphatic and fibrosis-related genes, including collagen type I alpha 1 (Col1a1), collagen type III alpha 1 (Col3a1), and transforming growth factor beta (Tgfb) by RTPCR. It should be interesting to confirm this data by Western

blotting and immunostaining in LECs exposed to No AFib-EAT and AFib-EAT conditioned media and in the AFib in vivo models. Moreover, since EndoMT can be induced through the canonical and non-canonical TGF-β signaling pathway, it will be interesting to investigate which via undergo LECs during EndoMT in AFib.

6. How can authors explain that metabolic dysfunction in LECs promotes EndoMT? Can authors perform additional functional investigations to confirm the previous statement?

Minor comments:

- In Figure 1K, are these results statistically significant? If yes, include p-value.
- In Figure 3B, are these results statistically significant? If yes, include p-value.
- In Figure 5K, the labeling in the X-axis is confusing.
- The first paragraph of pag.10 sounds repetitive with the second paragraph of pag.11.

Reviewer #3

(Remarks to the Author)
Interesting and very comprehensive experimental analysis of the hypothesis that Epicardial Adipose Tissue-derived kynurenic acid induces endothelial cell dysfunction in lymphatic vessels in atrial fibrillation patients.

Additional information needed to properly interpret the presented data:

What is the degree of SVF differentiation achieved in vitro and for the different origins (i.e. No AFib vs AFib patients)? This is best shown using a low magnification microscopy image to show the whole well / plate stained with Oil Red O or Bodipy. Adipogenic factor gene expression is useful when comparing time course of differentiation, not so much comparing two isolated time points. If the differentiation degree is different between SVF, that would introduce a confounding factor in the comparison. Also since preadipocytes that don't differentiate often adopt pro-inflammatory profiles.

It would also be good to have some additional information about the differentiation conditions / media used for the SVF so we can better understand what cells we are getting in the end. Is there Rosiglitazone in the media (which is known to induced browning of the adipcytes)? What is the amount of glucose and insulin used (are these cells made insulin-resistance but the culture conditions)?

What is the concentration of Kynurenic acid in the EAT biopsies obtained from the different origins? Without knowing actual concentration it becomes very difficult to determine if those levels would indeed be sufficient to affect GPR35 or other receptors.

Actual concentrations should be shown for Kynurenic acid throughout the manuscript, not just qualitative comparisons. Not needed to do this for every metabolite shown, but definitely for those that the paper stands on.

The fact that Ido1 in increased in the EAT and Kat1 and Kat2 are reduced, would suggest a shift to high kynurenine and 3-hydroxykynurenine, possibly even quinolinic acid and not kynurenic acid. What are the levels of those metabolites in the EAT and in the SVF-derived adipocyte cultures? If Kynurenine is up (and that is a much more accepted cytotoxic metabolite than kynurenic acid), then kynurenic acid will be also be elevated due to higher substrate availability, but then the most common consequence is toxicity from high kynurenine. In addition, high kynurenine will be converted into 3-hydroxykynurenine (also a known cytotoxic compound), unless there is a deficit in the enzyme that mediates that conversion (KMO). That should e investigated and shown.

What happens to the levels of Kat3 and Kat4?

Another possible endogenous ligand for GPR35 is CXCL17. What are the levels of that chemokine in the EAT and in the SVF-derived adipocyte cultures.

Version 1:

Reviewer comments:

Reviewer #1

(Remarks to the Author)
The authors addressed only partially my concerns and some critical issues remain:
- In response to my concerns that ventricular dysfunction could contribute to the observed arrhythmogenic phenotype, eventually being the primary mechanism of the increased AF susceptibility, the authors only assessed the components of

the NLRP3 system in the atria (new Fig. R1-4). However, this does not address my question. The is no functional assessment of the ventricular function in the individual mouse groups (echo, ventricular pressure, etc.), demonstrating absence of overt ventricular dysfunction feedbacking the atria independent of atrial lymphoangiogenesis.
- Fig. R1-3: The number of mice per group is very low, partuicularly in the Ang-II group. In D, right panel there is strong trend of increased RyR2 phosphorylation which is not significant because of lack of statistical power. This result does not support the claim of the authors that the lymphoangiogenesis changes precede the development of Ca2+-dependent triggered activity. This part need further experimentation with larger sample sizes to definitely address this issue.

Reviewer #2

(Remarks to the Author)
The authors have correctly addressed and clarified the points addressed by this reviewer. The manuscript has been improved with the additional experiments.

Reviewer #4

(Remarks to the Author)

1. In response to reviewer 3's comments, the authors provided satisfactory replies to comments #1, #2, and #7 by conducting additional experiments.

2. Regarding comment #3, the authors performed mass spectrometry-based analyses to determine the actual concentrations of kynurenic acid (KYNA) in both epicardial adipose tissue (EAT) and EAT-conditioned media from the No AFib and AFib groups. However, the reviewer specifically requested that "actual concentrations should be shown for kynurenic acid throughout the manuscript." The authors did not provide the actual KYNA concentrations in the contexts of AngII-induced or obesity-induced impaired lymphangiogenesis.The authors demonstrated that 50 nM and 500 nM KYNA upregulate GPR35 mRNA levels in lymphatic endothelial cells (LECs) by approximately 1.5-fold. Nevertheless, they did not report the actual GPR35 expression levels in LECs from the No AFib or AFib groups.

3. In response to comment #5, the authors showed that KYNA levels are elevated in AFib EAT tissue compared to No AFib controls. However, these findings appear inconsistent with the fact that Ido1 is increased in epicardial adipose tissue (EpiWAT), while Kat1 and Kat2 are reduced. The authors did not adequately address this discrepancy. Additionally, the relative abundance of kynurenine pathway metabolites was assessed in EAT from No AFib or permanent AF (PerAF) patients, with n=12 per group—a sample size that may be insufficient for robust conclusions.

Version 2:

Reviewer comments:

Reviewer #1

(Remarks to the Author)
Thank you for properly addressing my comments.

Reviewer #4

(Remarks to the Author)
The authors have addressed the points addressed by reviewer 3. The manuscript has been improved with the additional experiments and can be accepted.

Open Access This Peer Review File is licensed under a Creative Commons Attribution 4.0 International License, which permits use, sharing, adaptation, distribution and reproduction in any medium or format, as long as you give appropriate credit to the original author(s) and the source, provide a link to the Creative Commons license, and indicate if changes were made.
In cases where reviewers are anonymous, credit should be given to 'Anonymous Referee' and the source.
The images or other third party material in this Peer Review File are included in the article's Creative Commons license, unless indicated otherwise in a credit line to the material. If material is not included in the article's Creative Commons license and your intended use is not permitted by statutory regulation or exceeds the permitted use, you will need to obtain permission directly from the copyright holder.
To view a copy of this license, visit https://creativecommons.org/licenses/by/4.0/

Point-by-Point Responses to the Reviewers

__**Referee #1**__

Interesting study providing novel and clinically and theraputically relevant insights into AF pathophysiology.

Comment #1: *Patients cohorts, table S1: AF causes atrial remodeling. The time from last documented pAF episodes should be added to the pAF group of patients.*

Response: We thank the reviewer for this thoughtful comment. We recognize that the timing from last documented paroxysmal AFib episodes to sample collection could potentially affect tissue characteristics. However, accurate determination of this interval was challenging due to the frequent asymptomatic nature of pAF episodes, which may occur undetected between documented occurrences. Given these challenges in accurately determining the time from last documented pAF episodes, we discussed this issue as a limitation in this study (see Page 26).

Comment #2-1: *Both AF groups are dominated by valve disease patients, whereas the control groups consists mostly of CAD patients. Thus, the reported changes in lymphangionesis could be due to valve disease instead of AF per se.*

Response: Thank you for highlighting the possibility that lymphangiogenesis may be influenced by underlying valve disease. As many patients in the AFib group had valvular disease, we agree this may act as a confounding factor. Accordingly, we discussed this issue as a limitation in this study (see Page 26).

Comment #2-2: *In addition, perAF patients had lower eGFR pointing to presence of CKD. The latter is known to cause AF by activation of the NLRP3 inflammasome and thus inflammation (PMID: 37581942). Again, the changes in lymphangionesis could be a result of activation of he NLRP3 system due to CKD in LA of these patients. The perAF patients have also larger LA. The authors should also perform two-way ANOVA to see whether there is an interaction effect between LA size and AF and eGFR and AF on lymphangionesis.*

Response: We appreciate the insightful comment regarding the potential involvement of the NLRP3 pathway in lymphangiogenesis. As noted, both chronic kidney disease (CKD) and left atrial (LA) enlargement may activate inflammatory pathways, including the NLRP3 inflammasome, which could influence lymphangiogenesis. However, given the relatively small sample size ($n = 42$), the statistical power to detect interaction effects using two-way ANOVA or generalized linear models is limited. We believe that such interaction analyses may not yield reliable conclusions under these conditions.

Nevertheless, recognizing the importance of this suggestion, we performed exploratory analyses to examine the relationships between lymphangiogenesis and (1) eGFR, (2) AFib status (No AFib, PAF, PerAF), and (3) their interaction using generalized linear models. The results of the analyses are shown below.

Table R1: The relationship between LEC density and AF / eGFR

	parameter estimate±standard error	*p* value
PAF	26.9±19.3	0.1706
PerAF	-51.9±21.3	0.0197
PAF × eGFR	0.6±1.0	0.5442
PerAF × eGFR	-0.7±1.1	0.5330
eGFR	0.3±0.7	0.6489

PAF × eGFR: interaction term of PAF and eGFR

PerAF × eGFR: interaction term of PerAF and eGFR

The interaction terms were not statistically significant. A generalized linear model including AFib status, LA size, and their interaction term as explanatory variables was not conducted due to multicollinearity concerns, given the strong correlation between AFib status and LA size, which could compromise the validity of parameter estimates.
Accordingly, we discussed this issue as a limitation in this study (see Page 26).

Comment #3: *Patients cohorts, tables S2,3: the patient characteristcs are imcomplete (comorbidities, etc, analog to table S1) and similar considerations apply as above. The type of AF was not specified.*

Response: We thank the reviewer for pointing out the incomplete patient characteristics in Tables S2 and S3. We agree that comprehensive patient information, including comorbidities and AFib type specification, is important for proper interpretation of the results.
We have now updated Tables S2 and S3 to include more complete patient characteristics analogous to those presented in Table S1, including relevant comorbidities. Regarding AFib type, we have clarified that all AFib patients in these cohorts had persistent atrial fibrillation, and this has been explicitly stated in the revised tables.

.

Comment #4: *Fig. 1: Do patients with AF have a stronger activation of the NLRP3 inflammasome, as previously reported in RA of pAF, perAF patients (PMID:29802206) and in patients prone to postoperative AF (PMID:32762493).*

Response: As suggested, we have measured NLRP3, ASC, Casp1-p20, pro-Casp1, pro-IL1β, and IL-1β protein levels in the atrium of No AFib and PerAFib patients (**Fig. R1-1A**, below). We found that protein expression of NLRP3, ASC, and Casp1-p20/pro-Casp1 ratio was significantly higher in PerAFib patients compared to No AFib patients (**Fig. R1-1B, C**, below), consistent with previous reports (Yao et al. 2018; Heijman et al. 2020).

We have cited these important studies in our revised manuscript and these results are now shown in **Supplementary Figure S1A**.

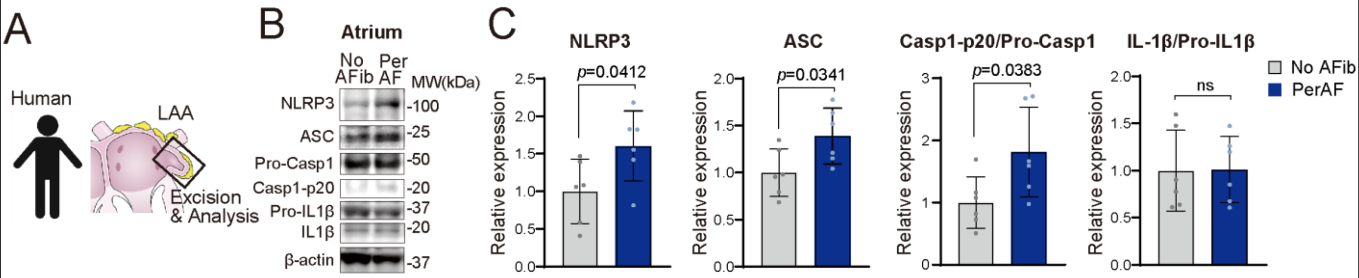

Fig. R1-1. A) Schematic illustration of the experiments in human left atrial appendage (LAA). LAA, left atrial appendage. **B)** Immunoblotting of NLRP3, ASC, Casp1-p20, Pro-Casp1, IL1β, and Pro-IL1β in the LAA of patients with No AFib and PerAF. β-actin was used as a loading control. Molecular weight (kDa) is shown on the right. **C)** Quantification of NLRP3, ASC, Casp1-p20, Pro-Casp1, IL1β, and Pro-IL1β protein in (B). $n = 6$ for No AFib or PerAF, biologically independent samples. Data are mean ± SD.; p value was determined by two-tailed unpaired Student's *t*-test. ns, not significant. NLRP3, NLR family pyrin domain containing 3. ASC, apoptosis-associated speck-like protein containing a CARD. Casp1, caspase1. IL1β, interleukine1β.

Comment #5: *AngII-induced AF model: The authors show that lymphangiogenesis is reduced, similarly to AF patients. It would be importnat to show whether the model recapitulates published alterations of the atria (PMID: 24030498): increased ROS generation, upregulated oxidized CaMKII, abnormal Ca2+-handling promoting triggered activity, etc.*

Response: As suggested, we conducted DHE staining and western blot analyses for MDA, oxidized CaMKII, and phosphorylated RyR2 in the atrium of control, AngII, and AngII+VEGFC mice (**Fig. R1-2A**, below). We found that ROS generation assessed by DHE staining and protein expression of MDA, ox-CaMKII, and p-RyR2 were significantly higher in AngII mice compared to control mice (**Fig. R1-2B-E**, below). These results confirm that our AngII-induced AFib model recapitulates the key pathophysiological alterations described in the literature, including increased oxidative stress and abnormal calcium handling. The data are now shown in **Supplementary Figure S7D-G**.

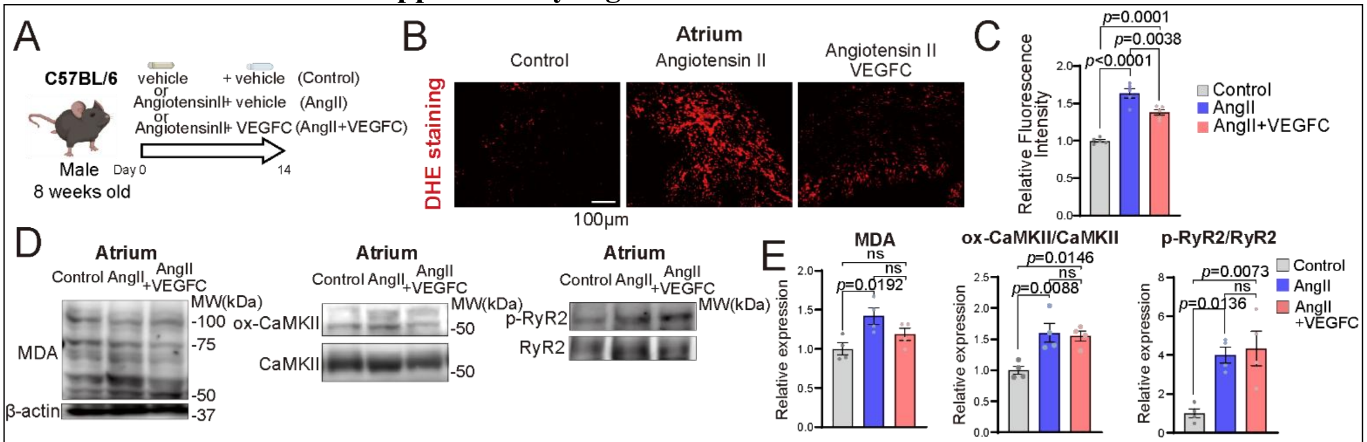

Fig. R1-2. A) Schematic illustration of the experiment procedure in mice. **B)** Representative DHE fluorescent staining in left atria. Scale bar, 100 μm. DHE, dihydroethidium. **C)** Quantification of fluorescence intensity of DHE in left atria from vehicle, Angiotensin II and/or VEGFC treated mice. n = 5 per group, biologically independent mice. Data are mean ± SEM.; p value was determined by one-way ANOVA followed by the Tukey-Kramer's *post hoc* test. **D)** Immunoblotting of MDA, oxidized-CaMKII, CaMKII, p-RyR2(Ser2814) and RyR2 in left atria Molecular weight (kDa) is shown on the right. **E)** Quantification of MDA, oxidized-CaMKII, CaMKII, p-RyR2 and RyR2 protein in (D). n = 4 per group, biologically independent mice. Data are mean ± SEM.; p value was determined by one-way ANOVA followed by the Tukey-Kramer's *post hoc* test. ns, not significant.

Comment #6: *It is also key to check whether the changes in lymphangiogenesis precede the changes in CaMKII signaling and its consequences. This is particularly important, because VEGFC rescued the proarrhythmic phenotype, pointing to lymphangiogenesis as being potentially the primary effect. However, it is possible that VEGFC improves the phenotype by acting on CaMKII and Ca2+-handling.*

Response: We appreciate this important question regarding the temporal relationship between lymphangiogenesis changes and CaMKII and Ca^{2+}-handling alterations. To address this question, we examined the expression of ox-CaMKII and p-RyR2 by western blot analysis at 1 week of AngII infusion, the same time point where we had previously demonstrated decreased lymphangiogenesis-related gene expression. We found that at this early time point (1 week), while lymphangiogenesis-related gene expression was already significantly reduced, there were no detectable changes in ox-CaMKII and p-RyR2 protein levels compared to control mice (**Fig. R1-3A-D**, next page). These results suggest that alterations in lymphangiogenesis precede the changes in CaMKII signaling and calcium handling abnormalities, supporting lymphangiogenesis as a potentially primary effect in AFib pathophysiology rather than a secondary consequence. The data are now shown in **Supplementary Figure S6B-D**. Furthermore, as shown in our response to **Comment #5**, VEGFC treatment did not significantly affect the levels of ox-CaMKII and p-RyR2 at 2 weeks (**Fig. R1-2**, above), despite its protective effects against AFib inducibility. This finding indicates that VEGFC's anti-arrhythmic effects are not mediated through direct modulation of CaMKII signaling or calcium handling, but rather through its primary effects on lymphangiogenesis.

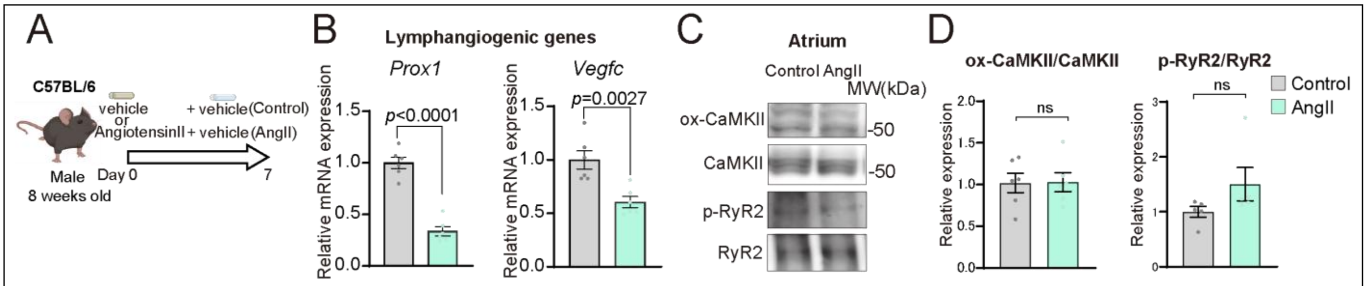

Fig. R1-3. A) Schematic illustration of the experiment procedure in mice. Male C57BL/6 mice at 8 weeks old received vehicle and/or Angiotensin II by osmotic pump for 7 days (Original Fig.S4A). **B)** Relative mRNA levels of lymphangiogenic genes in left atria from vehicle and/or Angiotensin II treated mice for 7 days. *n*= 6 per group, biologically independent mice. Data are mean ± SEM.; *p* value was determined by two-tailed unpaired Student's *t*-test (Original Fig.S4B). **C)** Immunoblotting of oxidized-CaMKII, CaMKII, p-RyR2 and RyR2 in left atria from vehicle or Angiotensin II treated mice. Molecular weight (kDa) is shown on the right. **D)** Quantification of oxidized-CaMKII, CaMKII, p-RyR2 and RyR2 protein in (C). *n* = 6 per group, biologically independent mice. Data are mean ± SEM.; *p* value was determined by two-tailed unpaired Student's *t*-test. ns, not significant.

Comment #7: *AngII-induced AF model: These mice have diastolic dysfunction pointing to a potential feedback of the ventricles on atrial function. The authors argue that lymphangiogenesis appears more important in the atria, but they ignore that other aspects of ventricular dysfuncton(ROS production, hemodynamic consequences, etc.) could determine the atrial phenotype. In addition, AngII activates the NLRP3 system in the ventricles as well (reviewed in PMID: 31441689).*

Response: We appreciate the reviewer's important point regarding the potential influence of ventricular dysfunction on atrial phenotype in our AngII model. To address this concern, we examined NLRP3 inflammasome activation in the atrium by measuring NLRP3, ASC, Casp1-p20, pro-Casp1, pro-IL1β, and IL-1β protein levels in the atrium of control, AngII, and AngII+VEGFC mice at 2 weeks of treatment (**Fig. R1-4A**, below). We found that AngII infusion significantly increased atrial NLRP3 inflammasome activation, but VEGFC treatment did not affect these atrial inflammatory markers (**Fig. R1-4B, C**, below). This suggests that while VEGFC effectively rescues the atrial AFib phenotype, the mechanism may not be directly through modulation of NLRP3 inflammasome signaling, supporting the importance of lymphangiogenesis as a distinct pathway in atrial pathophysiology. The data are now shown in **Supplementary Figure S7H, I**.

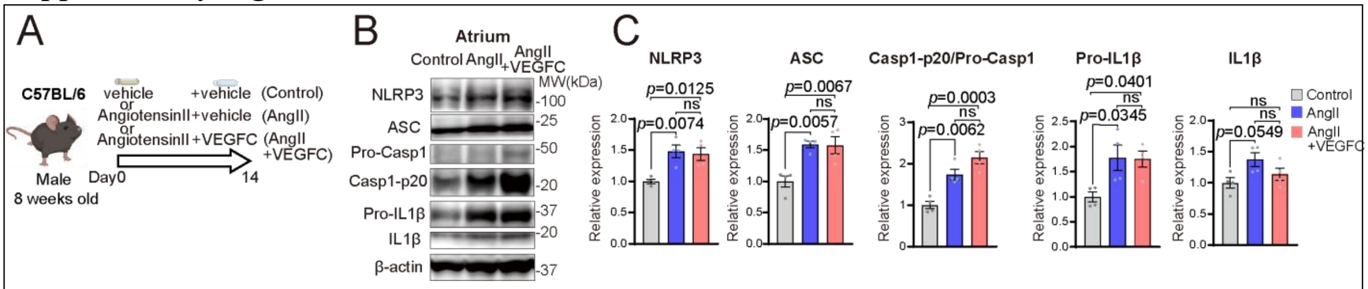

Fig. R1-4. A) Schematic illustration of the experiment procedure in mice. Male C57BL/6 mice at 8 weeks old received vehicle, Angiotensin II and/or VEGFC by osmotic pump for 14 days. **B)** Immunoblotting of NLRP3, ASC, Casp1-p20, Pro-Casp1, IL1β, and Pro-IL1β in left atria from vehicle, Angiotensin II and/or VEGFC treated mice. β-actin was used as a loading control. Molecular weight (kDa) is shown on the right. **C)** Quantification of NLRP3, ASC, Casp1-p20, Pro-Casp1, IL1β, and Pro-IL1β protein in (B). *n* = 4 per group, biologically independent mice. Data are mean ± SEM.; *p* value was determined by one-way ANOVA followed by the Tukey-Kramer's *post hoc* test. ns, not significant.

However, we acknowledge that we cannot completely exclude the potential influence of ventricular dysfunction and its hemodynamic consequences on the observed atrial phenotype. Accordingly, we discussed this issue as a limitation in this study (see Page 26).

Comment #8: *Obesity-induced AF model: Previous papers (PMID: 32654503; PMID: 33523143) showed that obesity in mice cause atrial electrical, structural and Ca2+-handling remodeling that promote AF. One key mechanism appears to be upregulation of the NLRP3 inflammasome system. Since genetic deletion of NLRP3 prevents the proarrhythmic substrate in obese mice, this could be interpreted as a potential mechanism upstream of impaired lymphangiogenesis. The authors claim that inflammatory remodeling does not contribute, but a comprehensive study of inflammatory signaling pathways and their time course were not demonstrated. Again, the effect of weight reduction on lymphangiogenesis and AF in the present obeity model could be a consequence of reduced inflammatory burden, with lymphangiogenesis being a bystander only. This reviewer did not find info on ventricular function in the obesity model.*

Response: We are grateful for the reviewer's thoughtful comments and suggestions. To address the reviewer's question, we have performed the following new experiments:

1) **Hi-Fat diet (13 weeks) + vehicle or LY3437943 (3 weeks) model:** We examined NLRP3 inflammasome activation in the atrium by measuring NLRP3, ASC, Casp1-p20, pro-Casp1, pro-IL1β, and IL-1β protein levels in the atrium of HFD-vehicle and HFD-LY3437943 mice (**Fig. R1-5A**, below). Interestingly, we found that the weight loss intervention did not lead to a significant difference in protein expression of NLRP3, ASC, Casp1-p20/pro-Casp1, and IL-1β/pro-IL1β ratio (**Fig. R1-5B, C**, below). These results are now shown in **Supplementary Figure S8A-C**.

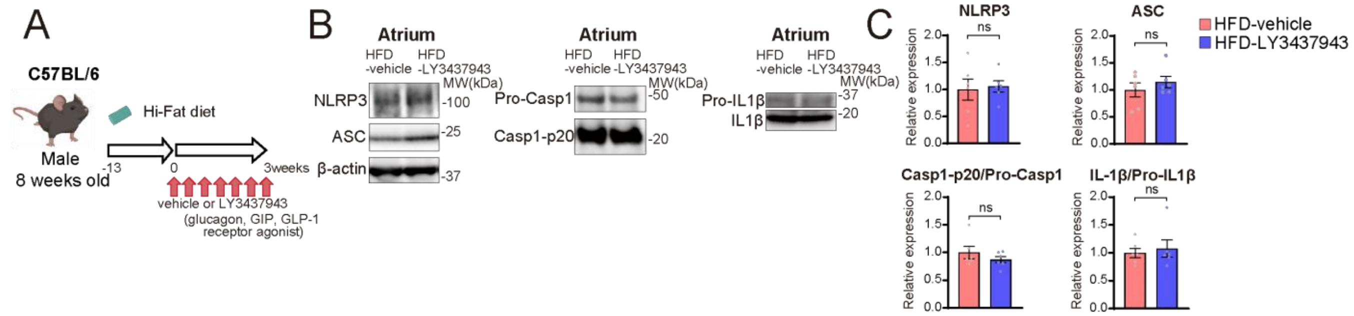

Fig. R1-5. A) Schematic illustration of the experiment for HFD-induced obese mice followed by administration of LY3437943. Male C57BL/6J mice at 8 weeks old were given access to Hi-fat diet (60 % fat) for 13 weeks. A subset of mice was then injected LY3437943 intraperitoneally every third day for 3 weeks. **B)** Immunoblotting of NLRP3, ASC, Casp1-p20, Pro-Casp1, IL1β, and Pro-IL1β in left atria from HFD-induced obese mice with or without LY3437943 treatment. β-actin was used as a loading control. Molecular weight (kDa) is shown on the right. **C)** Quantification of NLRP3, ASC, Casp1-p20, Pro-Casp1, IL1β, and Pro-IL1β protein in (B). *n* = 6 per group, biologically independent mice. Data are mean ± SEM.; *p* value was determined by two-tailed unpaired Student's *t*-test. ns, not significant.

2) **Hi-Fat diet (13 weeks) + vehicle or LY3437943 (1 week) model:** To examine the temporal relationship between lymphangiogenesis and inflammatory signaling, we analyzed both lymphangiogenesis-related and NLRP3-related gene expression at an earlier time point (1 week of LY3437943 treatment). We found that lymphangiogenesis-related gene expression was already significantly increased at this early time point, while NLRP3-related genes showed no detectable changes (**Fig. R1-6A, B**, below). These results are now shown in **Supplementary Figure S8D, E**.

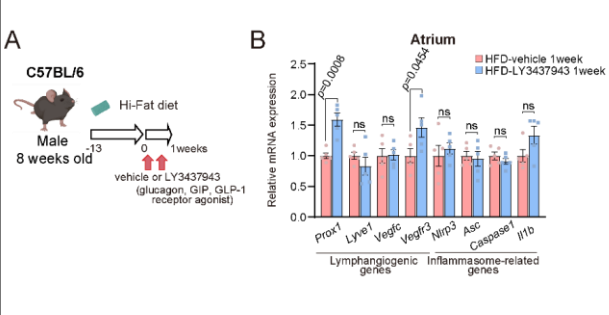

Fig. R1-6. A) Schematic illustration of the experiment. Male C57BL/6J mice were given access to Hi-fat diet for 13 weeks. A subset of mice was injected LY3437943 intraperitoneally every third day for 1 week. **B)** Relative mRNA levels of lymphangiogenic and inflammasome-related genes in left atria from obese mice with or without LY3437943 treatment. *n* = 5 per group, biologically independent mice. Data are mean ± SEM.; *p* value was determined by two-tailed unpaired Student's *t*-test. ns, not significant.

3) **Normal-Fat diet or Hi-Fat diet (4 weeks) model:** To further examine the early changes in obesity-induced AFib, we compared normal fat diet and HFD groups at 4 weeks (**Fig. R1-7A**, below). We found that lymphangiogenesis-related gene expression was already significantly decreased in HFD mice compared to normal chow controls, while NLRP3 inflammasome-related gene expression showed no detectable changes (**Fig. R1-7B**, below). Consistent with the gene expression data, Western blot analysis revealed no differences in NLRP3, ASC, Casp1-p20/pro-Casp1, and IL-1β/pro-IL1β protein levels between the groups at this early time point (**Fig. R1-7C, D**, below). These results are now shown in **Supplementary Figure S8F-I**.

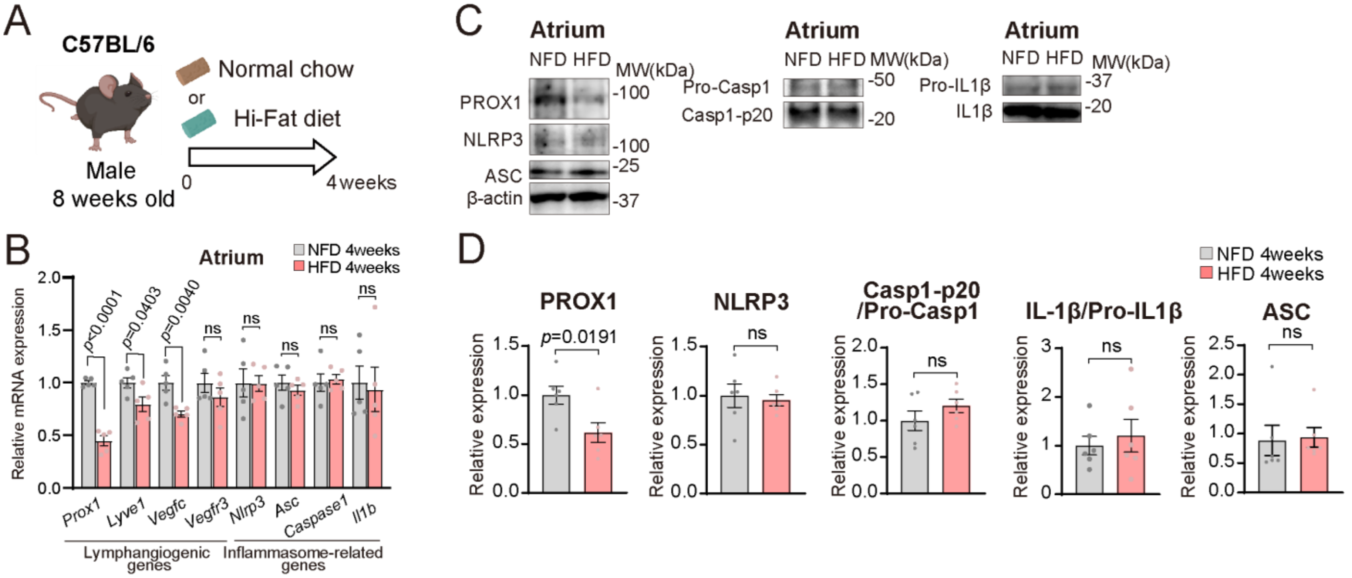

Fig. R1-7. A) Schematic illustration of the experiment for HFD-induced obese mice. Male C57BL/6J mice at 8 weeks old were given access to normal chow or Hi-fat diet (60 % fat) for 4 weeks. **B)** Relative mRNA levels of lymphangiogenic and inflammasome-related genes in left atria from mice fed normal chow (NFD) or hi-fat diet (HFD). $n = 5$ per group, biologically independent mice. Data are mean ± SEM.; p value was determined by two-tailed unpaired Student's t-test. ns, not significant. **C)** Immunoblotting of PROX1, NLRP3, ASC, Casp1-p20, Pro-Casp1, IL1β, and Pro-IL1β in left atria from mice fed NFD or HFD. β-actin was used as a loading control. Molecular weight (kDa) is shown on the right. **D)** Quantification of PROX1, NLRP3, ASC, Casp1-p20, Pro-Casp1, IL1β, and Pro-IL1β protein in (C). $n = 6$ per group, biologically independent mice. Data are mean ± SEM.; p value was determined by two-tailed unpaired Student's t-test. ns, not significant.

Collectively, these temporal analyses (experiments 1-3) demonstrate that alterations in lymphangiogenesis consistently precede NLRP3 inflammasome activation across different time points in our obesity model, supporting the concept that impaired lymphangiogenesis is a primary mechanism rather than a downstream consequence of inflammatory signaling in obesity-induced AFib pathophysiology.

Additionally, we have included ventricular function data assessed by echocardiography in **Supplementary Table S5** to address the reviewer's concern regarding ventricular function in the obesity model.

Referee #2

General: In this manuscript, authors identify defective atrial lymphangiogenesis as a crucial player in AFib pathogenesis. By using human samples and a broad in vivo, ex vivo and in vitro experimental battery, authors show that epicardial adipose tissue (EAT) from AFib patients generates kynurenic acid that impacts lymphatic endothelial cell metabolism and mitochondrial homeostasis through GPR35, finally inducing endothelial-to-mesenchymal transition. They also demonstrate how VEGFC treatment reduces AFib inducibility.
In general, this work is relevant in the heart and cardiac lymphatics fields and could have important/potential applications, but some clarifications and additional information should be provided by authors. Major comments: Although the manuscript presents interesting data, this reviewer propose adding some clarifications and performing additional experiments to complete or add value to the results included in the manuscript in its current form:

Comment #1: *Although the effect of kynurenic acid in the impaired lymphangiogenesis in AFib pathogenesis was not known yet, the role of lymphatic vessels in the pathophysiology of AFib has been already published by the same authors, fact that reduces the novelty of the present work. In a recent manuscript published in European Heart Journal in 2023 (Role of atrial lymphatics in atrial myocardial fibrosis and atrial fibrillation: a human and mouse study; M Takahashi, I Abe, H Yamasaki, T Harada, M Takano, H Kondo, Y Teshima, S Miyamoto, N Takahashi, European Heart Journal, Volume 44, Issue Supplement_2, ehad655.3171, https://doi.org/10.1093/eurheartj/ehad655.3171), the same authors describe the importance of lymphatic vessels in AFib. In that work, authors analysed human LAA samples from PAF and PeAF patients and showed that mRNA expression of lymphangiogenesis-related genes (LYVE1, PROX1, and VEGFR3) was significantly lower in the PeAF group compared to the PAF group. Moreover, they showed that the number of LYVE1-positive atrial lymphatic endothelial cells (LECs) was also lower in the PeAF group, and there was increased mRNA expression of fibrosis-related genes (Tgfb, Col1a1, and Col3a1). In mice, they use the same models used in this manuscript (transesophageal burst pacing and burst pacing in isolated perfused hearts using a Langendorff apparatus) and performed similar assays injecting mice with vehicle or AngII, and also, treating them with VEGFC. They saw that VEGFC treatment attenuated AngII-induced enhancement of vulnerability to AF in in vivo experiments.*
Despite the existence of the mentioned results published in https://doi.org/10.1093/eurheartj/ehad655.3171, which are very closed to the data presented here, there is no reference to that manuscript in the present work. Moreover, in the Abstract, authors mention "Here, we identify impaired atrial lymphangiogenesis as a critical determinant in AFib pathogenesis. Analysis of human left atrial appendage specimens reveals decreased lymphatic vessel density in AFib patients compared to those in sinus rhythm." This sentence is not fully correct as this characterization was already done in a previous work of the same authors (already published). Also, in the Abstract, authors mention "In vivo studies utilizing angiotensin II-induced and high-fat diet mouse models confirm the critical role of lymphatic dysfunction in AFib susceptibility. Therapeutic interventions promoting lymphangiogenesis, either through VEGFC administration or weight loss intervention by LY3437943 (the novel triple GIP, GLP-1, and glucagon receptor agonist), significantly attenuate AFib inducibility. These findings establish lymphatic dysfunction as a novel pathogenic mechanism in AFib and highlight lymphatic vessel formation as a promising therapeutic target." This sentence is not fully correct as these in vivo experiments (or part of them) were already performed in a previous authors´work (already published).
In the Introduction and Discussion sections, authors do not refer to the original manuscript published in European Heart Journal https://doi.org/10.1093/eurheartj/ehad655.3171), in which they identified the role of defective atrial lymphangiogenesis in AFib pathogenesis.

It is not clear for this reviewer which is the novel data related to this work in comparison with the manuscript already published. Please clarify.

Response: First of all, we thank the reviewer for bringing this important point to our attention. We would like to address the reviewer's concern regarding the referenced work (https://doi.org/10.1093/eurheartj/ehad655.3171) and clarify any potential confusion. We acknowledge that this citation refers to a meeting abstract presented at the ESC Congress 2023, not a peer-reviewed full manuscript.

While preliminary findings related to lymphangiogenesis in atrial fibrillation were presented in conference abstracts, the current manuscript represents the first comprehensive, peer-reviewed publication of this research with complete experimental details, statistical analyses, and mechanistic insights.

According to *Nature Communications* editorial policies, meeting abstracts do not constitute prior publication, and journals specifically allow publication of meeting abstracts before the full contribution is submitted. We have disclosed the existence of related meeting abstracts in our cover letter in accordance with journal requirements.

Comment #2: *In order to study the effect of conditioned media in the functional impairment of LECs, authors measured paracellular permeability and tube formation capacity. However, during the lymphangiogenic process, LECs also proliferate, migrate and invade the extracellular matrix, finally forming a new sprout and a nascent vessel. It would be great to evaluate the proliferation rate, migration and invasion properties and sprouting capacity of LECs by performing EdU assays, Boyden chamber assays, spheroid assays, etc. in presence of No AFib- or AFib-EAT conditioned media.*

Response: We are grateful for the reviewer's thoughtful comments and suggestions. As suggested, we have performed the following new experiments:

1) **EdU assays by Flow cytometry:** We assessed LEC proliferation using EdU incorporation assays analyzed by flow cytometry (**Fig. R2-1A**, below). AFib-EAT conditioned media significantly reduced LEC proliferation compared to No AFib-EAT conditioned media (**Fig. R2-1B, C**, below). Similarly, kynurenic acid treatment decreased LEC proliferation compared to vehicle control (**Fig. R2-1D, E**, below).

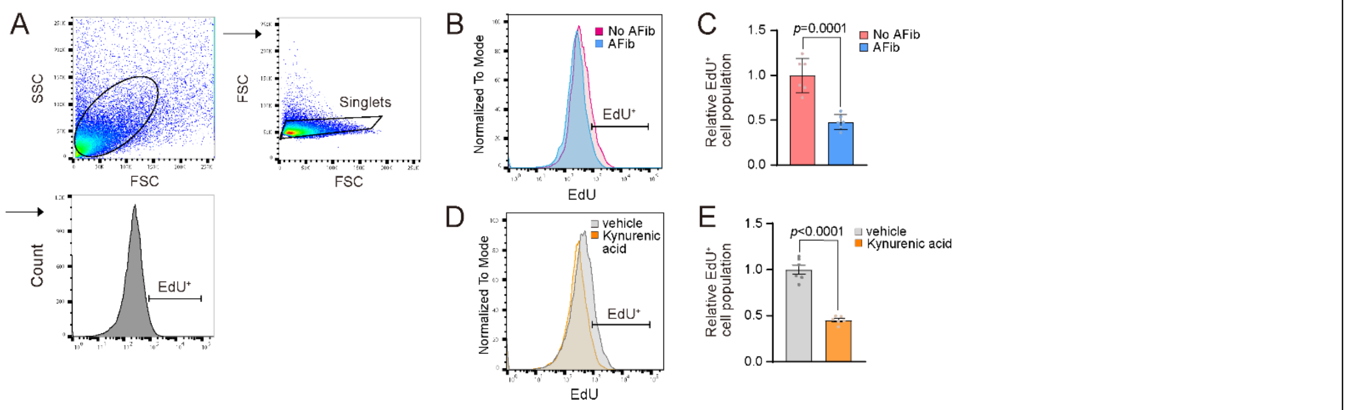

Fig. R2-1. A) Sequential gating strategy to discriminate EdU-positive LECs *in vitro*. We examined the proliferation of LECs based on the signal of EdU incorporation. **B)** Representative FACS histogram images of EdU^+ cell population in LECs treated with No AFib or AFib-EAT conditioned media. **C)** FACS-based quantification of EdU^+ cell population in LECs treated with No AFib or AFib-EAT conditioned media. $n = 6$ for each group, biologically independent samples. Data are mean ± SD.; p value was determined by two-tailed unpaired Student's t-test. **D)** Representative FACS histogram images of EdU^+ cell population in LECs treated with vehicle or Kynurenic acid. **E)** FACS-based quantification of EdU^+ cell population in LECs treated with vehicle or Kynurenic acid. $n = 6$ for each group, biologically independent samples. Data are mean ± SEM.; p value was determined by two-tailed unpaired Student's t-test. EdU, 5-ethynyl-2'-deoxyuridine.

2) **3D Spheroid Invasion assay:** We evaluated the invasive capacity of LECs using 3D spheroid assays. AFib-EAT conditioned media markedly impaired LEC invasion into the surrounding matrix compared to No AFib-EAT conditioned media (**Fig. R2-2A, B**, below). Kynurenic acid treatment also significantly reduced invasive capacity compared to vehicle control (**Fig. R2-2C, D**, below).

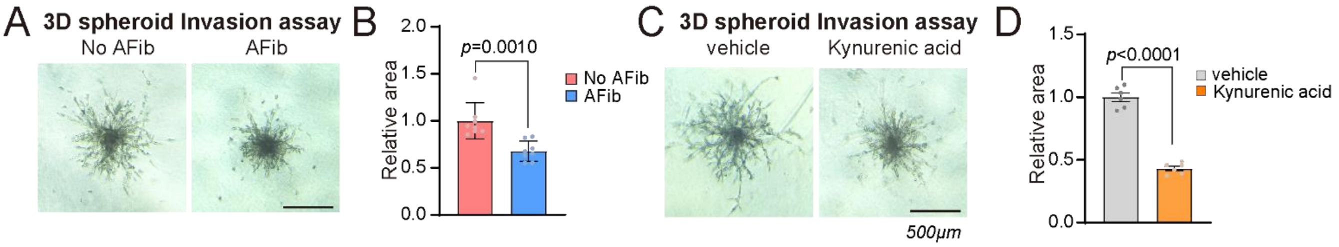

Fig. R2-2. A) Representative bright-field images of LECs spheroids embedded in invasion matrix treated with No AFib or AFib-EAT conditioned media. Scale bar, 500 µm. **B)** Quantitative analysis of area in (A). *n* = 8 for each group, biologically independent samples. Data are mean ± SD.; *p* value was determined by two-tailed unpaired Student's *t*-test. **C)** Representative bright-field images of LECs spheroids embedded in invasion matrix treated with vehicle or Kynurenic acid. Scale bar, 500 µm. **D)** Quantitative analysis of area in (C). *n* = 6 for each group, biologically independent samples. Data are mean ± SEM.; *p* value was determined by two-tailed unpaired Student's *t*-test.

3) **Migration assay:** We performed Boyden chamber migration assays to assess LEC migratory properties. AFib-EAT conditioned media substantially decreased LEC migration compared to No AFib-EAT conditioned media (**Fig. R2-3A**, below). Consistent with other functional parameters, kynurenic acid treatment reduced LEC migration compared to vehicle control (**Fig. R2-3B**, below).

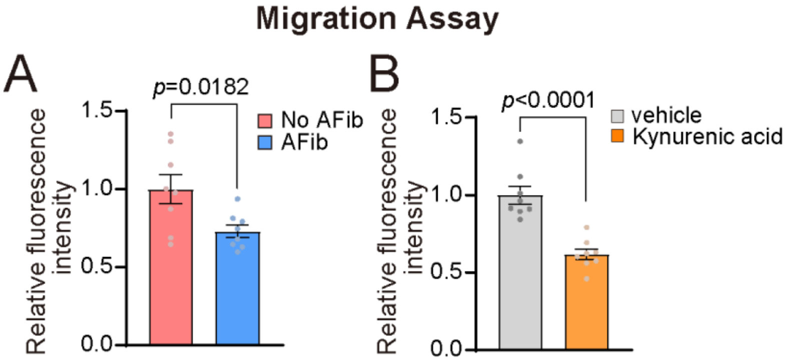

Fig. R2-3. A) Quantitative analysis of migrated LECs treated with No AFib or AFib-EAT conditioned media. Migrated cells were quantified using CyQuant® GR dye and measured by fluorescence (RFU). *n* = 8 for each group, biologically independent samples. Data are mean ± SD.; *p* value was determined by two-tailed unpaired Student's *t*-test. **B)** Quantitative analysis of migrated LECs treated with vehicle or Kynurenic acid. Migrated cells were quantified using CyQuant® GR dye and measured by fluorescence (RFU). *n* = 8 for each group, biologically independent samples. Data are mean ± SEM.; *p* value was determined by two-tailed unpaired Student's *t*-test.

These comprehensive functional assays demonstrate that AFib-EAT conditioned media impairs multiple aspects of lymphangiogenesis, including proliferation, invasion, and migration, supporting our hypothesis that AFib-associated factors comprehensively disrupt lymphatic vessel formation. The consistent effects of kynurenic acid across all assays further validate its role as a key mediator of lymphangiogenic dysfunction in AFib. These results are now shown in **Main Figure 2L, M**, **Supplementary Figure S3B-G, and S4A-C**.

Comment #3: *Although authors used liquid chromatography time-of-flight mass spectrometry to conduct a comprehensive metabolite profiling of No AFib-EAT and AFib-EAT conditioned media from human samples and identify the metabolite kynurenic acid, they do not measure the content of this metabolite in the in vivo models. It should be interesting to perform the same analysis in vivo to corroborate the data obtained by RTPCR about increased Ido1 expression and decreased Kat1 and Kat2 expression.*

Response: We appreciate the reviewer's excellent suggestion to validate our RT-PCR findings. As suggested, we performed mass spectrometry analysis to measure kynurenic acid and kynurenine levels in adipose tissue samples from our obesity model.
We found that LY3437943 treatment significantly decreased the kynurenic acid/kynurenine ratio in EpiWAT (visceral adipose tissue) compared to vehicle-treated controls (**Fig. R2-4**, below). Interestingly, this reduction was specific to visceral adipose tissue, as the kynurenic acid/kynurenine ratio in IngWAT (subcutaneous adipose tissue) remained unchanged following LY3437943 treatment.

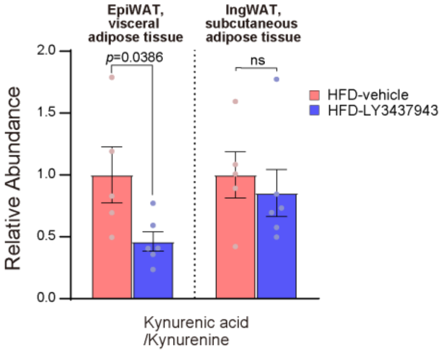

Fig. R2-4. Relative abundance of Kynurenic acid/Kynurenine ratio in epididymal white adipose tissue (EpiWAT, visceral adipose tissue) and inguinal white adipose tissue (IngWAT, subcutaneous adipose tissue) from obese mice with or without LY3437943 treatment. n = 5-6 for each group, biologically independent mice. Data are mean ± SEM.; p value was determined by two-tailed unpaired Student's t-test.

These results are consistent with our RT-PCR findings, which demonstrated that LY3437943 treatment led to a significant downregulation of *Kat1* and *Kat2* specifically in EpiWAT. Since these enzymes are responsible for converting kynurenine to kynurenic acid, the observed decrease in the kynurenic acid/kynurenine ratio provides direct biochemical validation of our gene expression data. The tissue-specific nature of these changes further supports the particular importance of visceral adipose tissue in AFib pathophysiology and lymphangiogenic dysfunction. These results are now shown in **Main Figure 5O**.

Comment #4: *Authors claim that LECs in AFib had compromised mitochondrial homeostasis, evidenced by shortened and swollen mitochondria with reduced cristae density after performing transmission electron microscopy. This reviewer suggests performing additional tests to more deeply evaluate mitochondrial homeostasis, such as mitochondrial respiration by Seahorse, mitochondrial membrane potential, mitochondrial ROS, etc..*

Response: We thank the reviewer for these valuable suggestions to further characterize mitochondrial dysfunction in our study. As suggested, we have performed the following comprehensive mitochondrial functional analyses:

1) **Mitochondrial respiration by Seahorse assay:** We assessed mitochondrial respiratory capacity using the Seahorse XF analyzer. LECs treated with AFib-EAT conditioned media showed significantly reduced oxygen consumption rate compared to those treated with No AFib-EAT conditioned media following FCCP treatment (**Fig. R2-5A**, next page). Furthermore, kynurenic acid treatment demonstrated a dose-dependent reduction in oxygen consumption rate at concentrations of 50 nM and 500 nM, confirming the direct impact of this metabolite on mitochondrial respiratory function (**Fig. R2-5B**, next page).

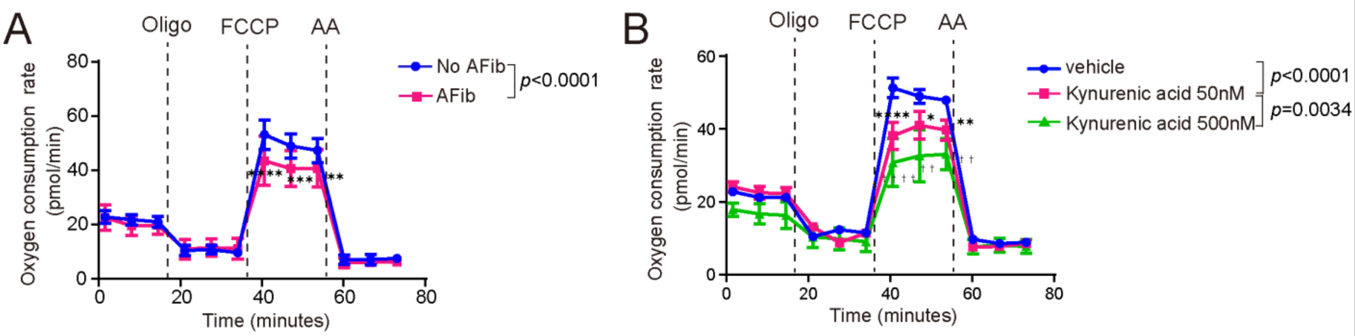

Fig. R2-5. A) Changes in oxygen consumption rate (OCR) in LECs treated with No AFib or AFib-EAT conditioned media. Cells were treated with 2.5 µM oligomycin followed by phenylhydrazone (FCCP, 2 µM), and antimycin (0.5 µM). $n = 9$ for both groups, biologically independent samples. Data are mean ± SD.; p value was determined by two-way repeated-measures ANOVA followed by Sidak's multiple comparison test. ** $p < 0.01$, *** $p < 0.001$, **** $p < 0.0001$ (*vs* No AFib). **B)** Changes in OCR in LECs treated with vehicle or Kynurenic acid at 50, and 500 nM. Cells were treated with 2.5 µM oligomycin followed by phenylhydrazone (FCCP, 2 µM), and antimycin (0.5 µM). $n = 3$ for both groups, biologically independent samples. Data are mean ± SEM.; p value was determined by two-way repeated-measures ANOVA followed by Sidak's multiple comparison test. * $p < 0.05$, ** $p < 0.01$, **** $p < 0.0001$ (*vs* vehicle), and $^{\dagger\dagger}p < 0.01$, $^{\dagger\dagger\dagger\dagger}p < 0.0001$ (*vs* vehicle).

2) **Mitochondrial membrane potential and mitochondrial ROS by JC-1 and mtSOX assays:** We evaluated mitochondrial membrane potential using JC-1 staining and mitochondrial ROS production using mtSOX assays. LECs treated with AFib-EAT conditioned media exhibited significantly decreased mitochondrial membrane potential and increased mitochondrial ROS production compared to No AFib-EAT conditioned media-treated controls (**Fig. R2-6A**, below). Similarly, kynurenic acid treatment resulted in reduced mitochondrial membrane potential and elevated mitochondrial ROS levels, providing direct evidence for kynurenic acid-mediated mitochondrial dysfunction (**Fig. R2-6B**, below).

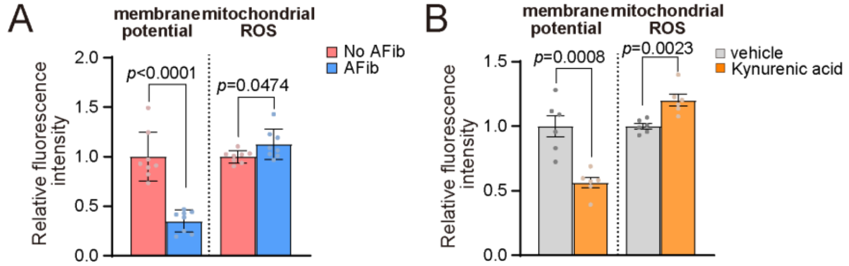

Fig. R2-6. A) Quantification of mitochondrial membrane potential measured as JC-1 red/green fluorescence intensity ratio, and mitochondrial superoxide levels measured as MitoBright ROS Deep Red fluorescence intensity in LECs treated with No AFib or AFib-EAT conditioned media. $n = 8$ for each group, biologically independent samples. Data are mean ± SD.; p value was determined by two-tailed unpaired Student's t-test. **B)** Quantification of mitochondrial membrane potential measured as JC-1 red/green fluorescence intensity ratio, and mitochondrial superoxide levels measured as MitoBright ROS Deep Red fluorescence intensity in LECs treated with vehicle or Kynurenic acid. $n = 6$ for each group, biologically independent samples. Data are mean ± SEM.; p value was determined by two-tailed unpaired Student's t-test.

These comprehensive mitochondrial functional assessments corroborate our transmission electron microscopy findings and establish that AFib-associated factors, particularly kynurenic acid, induce profound mitochondrial dysfunction in LECs through impaired respiratory capacity, compromised membrane potential, and increased oxidative stress. These results are now shown in **Main Figure 2F, Q, and Supplementary Figure S3L, S4M**.

Comment #5-1: *Authors claim that LECs in AFib undergo Endothelial-to-Mesenchymal Transition (EndoMT). However, they only analysed the expression of lymphatic and fibrosis-related genes, including collagen type I alpha 1 (Col1a1), collagen type III alpha 1 (Col3a1), and transforming growth factor beta (Tgfb) by RTPCR. It should be interesting to confirm this data by Western blotting and immunostaining in LECs exposed to No AFib-EAT and AFib-EAT conditioned media and in the AFib in vivo models.*

Response: This is a valid point. To address this concern, we have performed the following analyses:

1) ***In vitro* analyses for Immunofluorescence staining and western blot analyses:** We performed immunofluorescence staining to evaluate EndoMT markers in LECs. AFib-EAT conditioned media-treated LECs showed significantly increased TAGLN (transgelin) protein expression compared to No AFib-EAT conditioned media-treated LECs (**Fig. R2-7A, B**, below). Western blot analysis confirmed these findings, demonstrating reduced PROX1 (lymphatic endothelial marker) expression and increased TAGLN protein expression in LECs exposed to AFib-EAT conditioned media (**Fig. R2-7C, D**, below). Similarly, kynurenic acid treatment resulted in increased TAGLN protein expression by immunofluorescence staining and reduced PROX1 expression with elevated TAGLN protein levels by western blot analysis, confirming the direct role of this metabolite in promoting EndoMT (**Fig. R2-8A-D**, below). The results are now shown in **Supplementary Figure S3H-K, and S4D-G**.

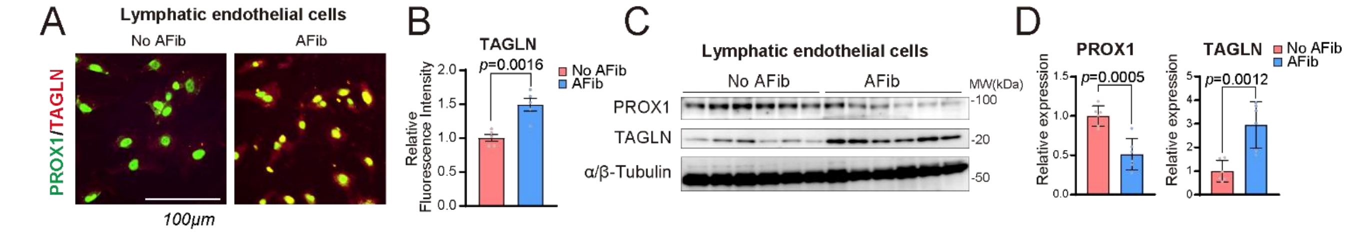

Fig. R2-7. A) Representative immunofluorescent staining for PROX1 (Green)/TAGLN (Red) of LECs treated with No AFib or AFib-EAT conditioned media. Scale bar, 100 µm. **B)** Quantification of fluorescence intensity of TAGLN in LECs treated with No AFib or AFib-EAT conditioned media. $n = 5$ per group. Data are mean ± SD.; p value was determined by two-tailed unpaired Student's t-test. **C)** Immunoblotting of PROX1, and TAGLN in LECs treated with No AFib or AFib-EAT conditioned media. α/β-Tubulin was used as a loading control. Molecular weight (kDa) is shown on the right. **D)** Quantification of PROX1, and TAGLN protein in (C). $n = 6$ per group, biologically independent samples. Data are mean ± SD.; p value was determined by two-tailed unpaired Student's t-test. PROX1, Prospero homeobox protein 1. TAGLN, Transgelin.

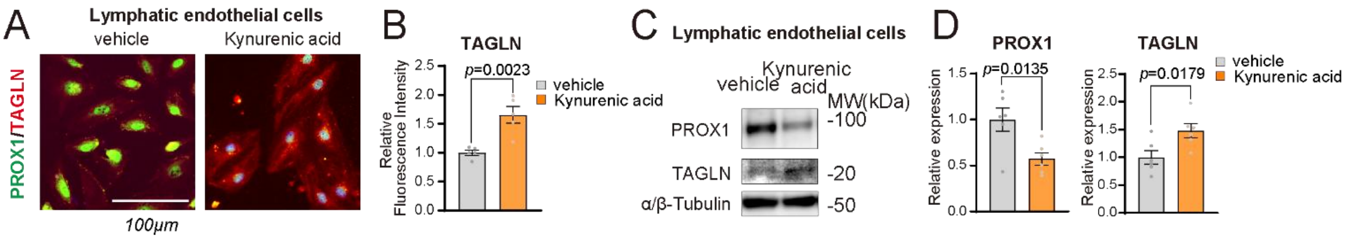

Fig. R2-8. A) Representative immunofluorescent staining for PROX1 (Green)/TAGLN (Red) of LECs treated with vehicle or Kynurenic acid. Scale bar, 100 µm. **B)** Quantification of fluorescence intensity of TAGLN in LECs treated with vehicle or Kynurenic acid. $n = 5$ per group. Data are mean ± SEM.; p value was determined by two-tailed unpaired Student's t-test. **C)** Immunoblotting of PROX1, and TAGLN in LECs treated with vehicle or Kynurenic acid. α/β-Tubulin was used as a loading control. Molecular weight (kDa) is shown on the right. **D)** Quantification of PROX1, and TAGLN protein in (C). $n = 6$ per group, biologically independent samples. Data are mean ± SEM.; p value was determined by two-tailed unpaired Student's t-test. PROX1, Prospero homeobox protein 1. TAGLN, Transgelin.

2) ***In vivo* analyses for immunostaining:** We performed immunofluorescence staining of atrial tissue from our mouse models to evaluate EndoMT *in vivo*. Angiotensin II-treated mice showed significantly increased PROX1 and TAGLN double-positive areas in atrium compared to control mice, indicating ongoing EndoMT (**Fig. R2-9A, B**, below). Importantly, LY3437943 treatment significantly reduced the PROX1 and TAGLN double-positive areas, demonstrating that weight loss intervention can reverse the EndoMT process in atrial lymphatic vessels (**Fig. R2-9C, D**, below).

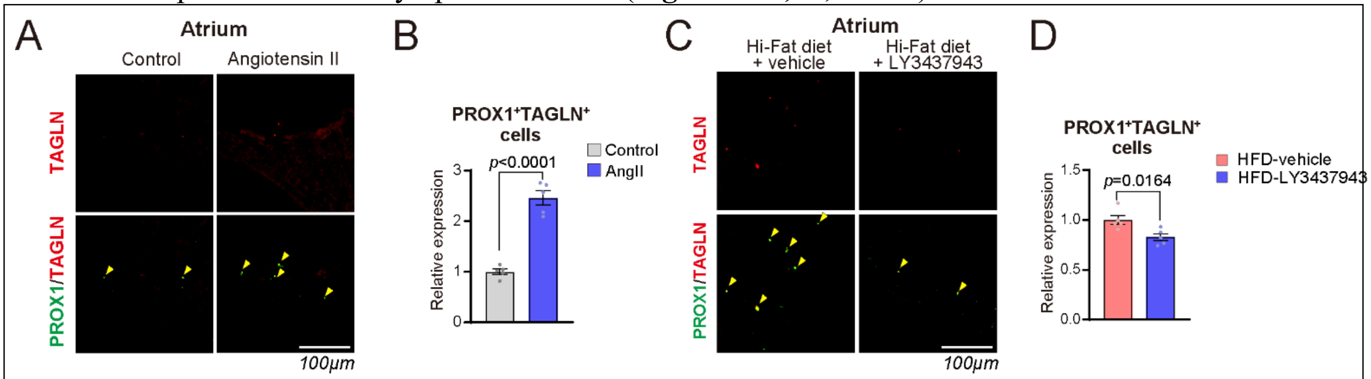

Fig. R2-9. A) Representative immunofluorescent staining for PROX1 (Green)/TAGLN (Red) in left atria from vehicle or Angiotensin II treated mice. Scale bar, 100 μm. **B)** Quantification of PROX1$^+$ TAGLN$^+$ cells in (A). $n = 5$ per group. Data are mean ± SEM.; p value was determined by two-tailed unpaired Student's *t*-test. **C)** Representative immunofluorescent staining for PROX1 (Green)/TAGLN (Red) in left atria from HFD-induced obese mice with or without LY3437943 treatment. Scale bar, 100 μm. **D)** Quantification of PROX1$^+$ TAGLN$^+$ cells in (C). $n = 5$ per group. Data are mean ± SEM.; p value was determined by two-tailed unpaired Student's *t*-test. PROX1, Prospero homeobox protein 1. TAGLN, Transgelin.

These comprehensive protein-level analyses confirm our gene expression findings and provide evidence for EndoMT occurring in atrial lymphatic endothelial cells both *in vitro* and *in vivo*.

Comment #5-2: *Moreover, since EndoMT can be induced through the canonical and non-canonical TGF-β signaling pathway, it will be interesting to investigate which via undergo LECs during EndoMT in AFib.*

Response: We appreciate this insightful suggestion to investigate the mechanistic pathways underlying EndoMT in our model. To address this question, we performed the following analyses:

1) **Western blot analysis of TGF-β signaling pathways:** We treated LECs with kynurenic acid and analyzed both canonical and non-canonical TGF-β signaling components by Western blot. Kynurenic acid treatment significantly increased the phosphorylation of SMAD2/3, TAK1, ERK1/2, and p38MAPK compared to vehicle control (**Fig. R2-10A, B**, below).

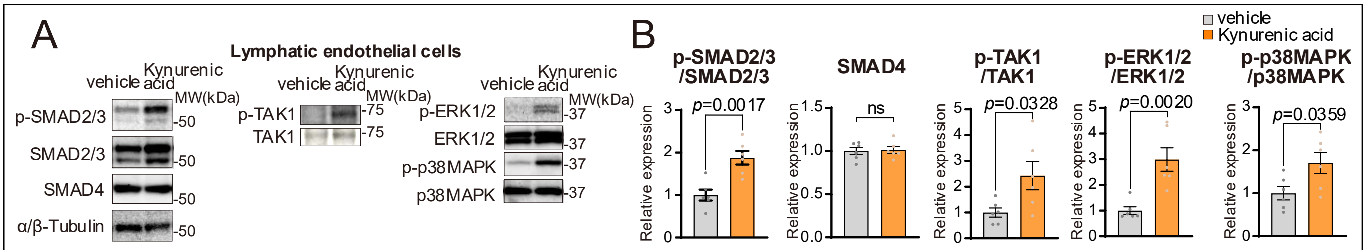

Fig. R2-10. A) Immunoblotting of p-SMAD2/3, SMAD2/3, SMAD4, p-TAK1, TAK1, p-ERK1/2, ERK1/2, p-p38MAPK, and p38MAPK in LECs treated with vehicle or Kynurenic acid. α/β-Tubulin was used as a loading control. Molecular weight (kDa) is shown on the right. **B)** Quantification of p-SMAD2/3, SMAD2/3, SMAD4, p-TAK1, TAK1, p-ERK1/2, ERK1/2, p-p38MAPK, and p38MAPK protein in (A). $n = 6$ per group, biologically independent samples. Data are mean ± SEM.; p value was determined by two-tailed unpaired Student's *t*-test. ns, not significant. SMAD2/3, SMAD family member 2/3. SMAD4, SMAD family member 4. TAK1, transforming growth factor beta-activated kinase 1. ERK1/2, extracellular signal-regulated kinase 1/2. p38MAPK, p38 mitogen-activated protein kinase.

2) **Pathway inhibition studies:** To determine the functional significance of these pathways, we co-treated LECs with kynurenic acid plus either SMAD2/3 inhibitor or TAK1 inhibitor and analyzed gene expression of EndoMT markers. SMAD2/3 inhibition effectively prevented kynurenic acid-induced downregulation of lymphatic markers (PROX1 and LYVE1) and suppressed the upregulation of the mesenchymal marker TAGLN (**Fig. R2-11**, below). In contrast, TAK1 inhibition did not prevent these kynurenic acid-induced changes in gene expression.

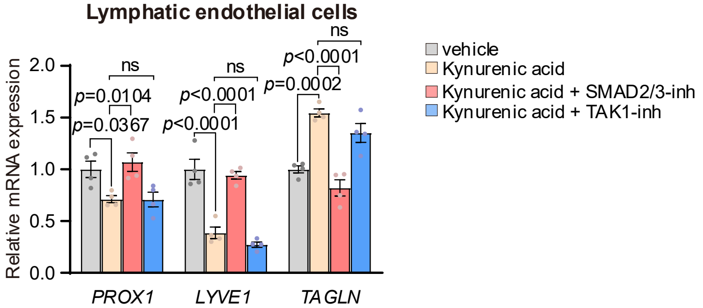

Fig. R2-11. Relative mRNA levels of lymphatic endothelial marker, and mesenchymal marker genes in LECs treated by vehicle and kynurenic acid with SMAD2/3-inhibitor (SB431542) or TAK1-inhibitor (Takinib). *n* = 4 per group, biologically independent mice. Data are mean ± SEM.; *p* value was determined by one-way ANOVA followed by the Dunnet's *post hoc* test. ns, not significant.

These results demonstrate that kynurenic acid promotes EndoMT in LECs primarily through the canonical TGF-β/SMAD2/3 signaling pathway, while the non-canonical TAK1 pathway appears to play a less critical role in this process. This mechanistic insight provides important therapeutic implications, as targeting the SMAD2/3 pathway may represent a specific approach to prevent lymphatic dysfunction in AFib. These results are now shown in **Supplementary Figure S4H-J**.

Comment #6: *How can authors explain that metabolic dysfunction in LECs promotes EndoMT? Can authors perform additional functional investigations to confirm the previous statement?*

Response: This is an excellent and important question that addresses a key mechanistic link in our study. To directly investigate whether metabolic dysfunction in LECs promotes EndoMT, we performed the following targeted experiments:

1) **BCAA depletion study:** We created a specialized culture medium depleted of branched-chain amino acids (BCAAs: valine, leucine, and isoleucine) and evaluated LECs at 12 and 24 hours to assess the direct impact of metabolic stress on EndoMT. At 12 hours of BCAA depletion, we observed significant downregulation of LYVE1 expression and upregulation of TAGLN expression (**Fig. R2-12**, below). By 24 hours, both LYVE1 and PROX1 showed significant downregulation, while TAGLN expression was significantly increased compared to complete medium controls.
2) **Time-course analysis:** The temporal analysis revealed that LYVE1 and PROX1 mRNA expression progressively decreased over time with BCAA depletion, while TAGLN expression showed progressive upregulation. This time-dependent pattern demonstrates a direct causal relationship between metabolic dysfunction and EndoMT initiation in LECs.

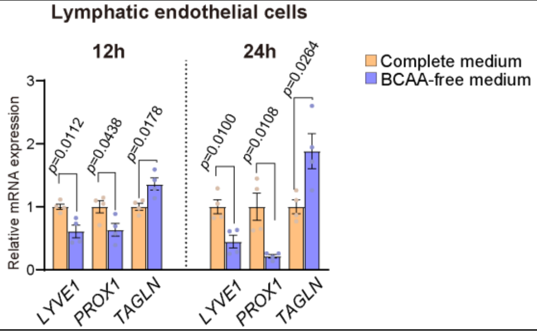

Fig. R2-12. Relative mRNA levels of lymphatic endothelial marker, and mesenchymal marker genes in LECs cultured in complete medium or branched chain amino acid (BCAA)-free medium for 12 or 24 hours. *n* = 4 per group, biologically independent samples. Data are mean ± SEM.; *p* value was determined by two-tailed unpaired Student's *t*-test.

These results provide compelling evidence that metabolic dysfunction, specifically BCAA depletion-induced metabolic stress, is sufficient to trigger EndoMT in LECs. This mechanistic insight explains how the metabolic alterations can directly promote the pathological transformation of lymphatic endothelial cells. These results are now shown in **Supplementary Figure S5A**.

Minor comments:

- In Figure 1K, are these results statistically significant? If yes, include p-value.
- In Figure 3B, are these results statistically significant? If yes, include p-value.
Response: Thank you for these important clarification questions. Both Figures represent visualizations of LEC population percentages identified within their respective UMAP plots, which show the combined data from all groups in each analysis. Since these represent cell population distribution rather than comparative data between groups, statistical significance testing is not applicable for these analyses.
To improve clarity and avoid confusion, we have modified the presentation to show only the UMAP plots themselves, with the percentage values now included in the Figure Legends. This approach more accurately represents the nature of the data as analyses of cell population composition within the single-cell RNA sequencing datasets.

- In Figure 5K, the labeling in the X-axis is confusing.
Response: We agree that the X-axis labeling in **Original Figure 5K** was confusing and appreciate the reviewer's feedback. We have revised the figure to provide clearer and more intuitive labeling that better represents the experimental conditions and enhances the readability of the data presentation.

- The first paragraph of pag.10 sounds repetitive with the second paragraph of pag.11.

Response: We thank the reviewer for this careful observation. Upon review, we identified an error in the text description that caused the repetitive appearance. In the second paragraph of page 11, the quantitative analysis of mitochondrial shape complexity (**Original Figure. 2M**, **Revised Figure. 2P**) was incorrectly described as comparing AFib-EAT conditioned media versus No AFib-EAT conditioned media, when it should have described the comparison between kynurenic acid treatment versus vehicle control, as correctly shown in the actual figure.
We have corrected this inconsistency in the manuscript text to accurately reflect that **Revised Figure. 2P** shows the mitochondrial form factor analysis comparing kynurenic acid-treated LECs versus vehicle-treated controls, which eliminates the repetitive content and ensures consistency between the text description and the figure presentation.

Referee #3

Interesting and very comprehensive experimental analysis of the hypothesis that Epicardial Adipose Tissue-derived kynurenic acid induces endothelial cell dysfunction in lymphatic vessels in atrial fibrillation patients. Additional information needed to properly interpret the presented data:

Comment #1: *What is the degree of SVF differentiation achieved in vitro and for the different origins (i.e. No AFib vs AFib patients)? This is best shown using a low magnification microscopy image to show the whole well / plate stained with Oil Red O or Bodipy. Adipogenic factor gene expression is useful when comparing time course of differentiation, not so much comparing two isolated time points. If the differentiation degree is different between SVF, that would introduce a confounding factor in the comparison. Also since preadipocytes that don't differentiate often adopt pro-inflammatory profiles.*

Response: We are grateful for the reviewer's positive comments and thoughtful suggestions. As suggested, we performed a comprehensive evaluation of SVF differentiation efficiency to address this important concern. We cultured SVF from both No AFib and AFib patients under identical adipogenic differentiation conditions and assessed differentiation using Oil Red O staining.

Low magnification microscopy images of the entire well showed comparable Oil Red O staining patterns between No AFib and AFib-derived SVF cultures (**Fig. R3-1A**, below). Quantitative analysis using spectrophotometric measurement of Oil Red O absorbance confirmed that there was no significant difference in adipogenic differentiation efficiency between SVF isolated from No AFib versus AFib patients (**Fig. R3-1B**, below). This demonstrates that both groups achieved similar degrees of differentiation under our experimental conditions. These results are now shown in **Supplementary Figure S2E, F**.

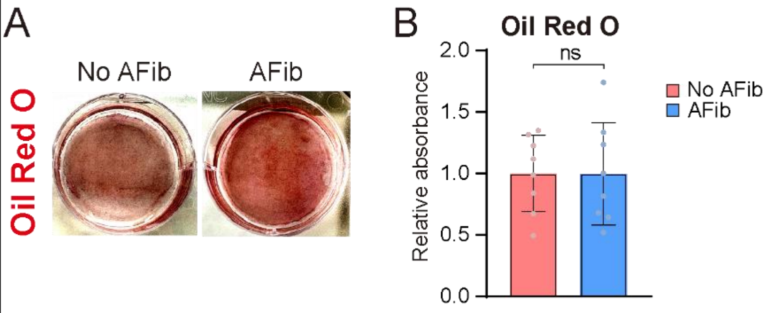

Fig. R3-1. A) Representative Oil Red O staining images of differentiated SVF from patients with No AFib and AFib. **B)** Quantitative analysis of lipid accumulation in differentiated SVF from the patients with No AFib and AFib. Optical density was measured by using a spectrophotometer. n = 8 per group, biologically independent samples. Data are mean ± SD.; *p* value was determined by two-tailed unpaired Student's *t*-test. ns, not significant.

Comment #2: *It would also be good to have some additional information about the differentiation conditions / media used for the SVF so we can better understand what cells we are getting in the end. Is there Rosiglitazone in the media (which is known to induced browning of the adipcytes)? What is the amount of glucose and insulin used (are these cells made insulin-resistance but the culture conditions)?*

Response: Thank you for this important question about our differentiation conditions. The differentiation medium contains the following components: d-Biotin (8 µg/ml), recombinant human insulin (0.5 µg/ml), dexamethasone (400 ng/ml), IBMX (44 µg/ml), L-Thyroxine (9 ng/ml), and ciglitazone (3 µg/ml). The nutrition medium contains recombinant human insulin (0.5 µg/ml). The glucose concentration in the media is 5.5 mM. Some of these concentrations, particularly insulin and the inclusion of ciglitazone, represent supraphysiological levels compared to normal circulating concentrations. These concentrations are standard for *in vitro* adipocyte differentiation protocols but exceed physiological ranges. Accordingly, we discussed this issue as a limitation in this study (see Page 26).

Comment #3: *What is the concentration of Kynurenic acid in the EAT biopsies obtained from the different origins? Without knowing actual concentration it becomes very difficult to determine if those levels would indeed be sufficient to affect GPR35 or other receptors. Actual concentrations should be shown for Kynurenic acid throughout the manuscript, not just qualitative comparisons. Not needed to do this for every metabolite shown, but definitely for those that the paper stands on.*

Response: We thank the reviewer for this important point regarding kynurenic acid concentrations. We have performed the following analyses to address this concern:

1. **Mass spectrometry-based analysis of kynurenic acid concentrations:** We performed mass spectrometry-based analysis to determine the actual kynurenic acid concentrations in both EAT and EAT-conditioned media from No AFib and AFib groups (**Fig. R3-2A, B**, below). These results are now shown in **Supplementary Figure S10A, B**. The measured concentrations in EAT-conditioned media were comparable to physiological blood concentrations reported in the literature (Favennec et al. 2016).

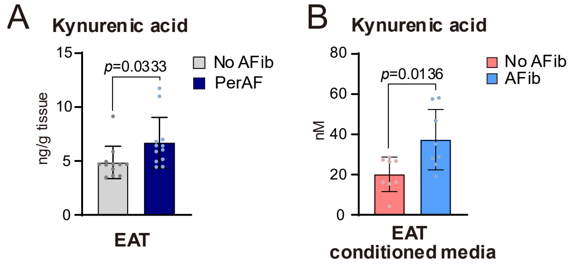

Fig. R3-2. A) Kynurenic acid concentrations in EAT from No AFib or PerAF patients. $n = 12$ per group, biologically independent samples. Data are mean ± SD.; p value was determined by two-tailed unpaired Student's t-test. **B)** Kynurenic acid concentrations in No AFib or AFib-EAT conditioned media. $n = 8$ per group, biologically independent samples. Data are mean ± SD.; p value was determined by two-tailed unpaired Student's t-test.

2. ***GPR35* expression is upregulated by AFib-EAT conditioned media:** We found that *GPR35* mRNA expression was significantly higher in lymphatic endothelial cells (LECs) treated with AFib-EAT conditioned media compared to those treated with No AFib-EAT conditioned media, suggesting enhanced sensitivity to kynurenic acid in the AFib setting (**Fig. R3-3A**, below).

3. **Chronic exposure effects on *GPR35* expression:** To assess the impact of prolonged kynurenic acid exposure, we treated lymphatic endothelial cells (LECs) with 5, 50, and 500 nM kynurenic acid for 4 days. While 5 nM showed no effect, both 50 nM and 500 nM significantly upregulated *GPR35* expression. This indicates that chronic exposure to kynurenic acid can enhance receptor expression, potentially creating a positive feedback loop that amplifies signaling sensitivity (**Fig. R3-3B**, below). These results are now shown in **Supplementary Figure S5B, C**.

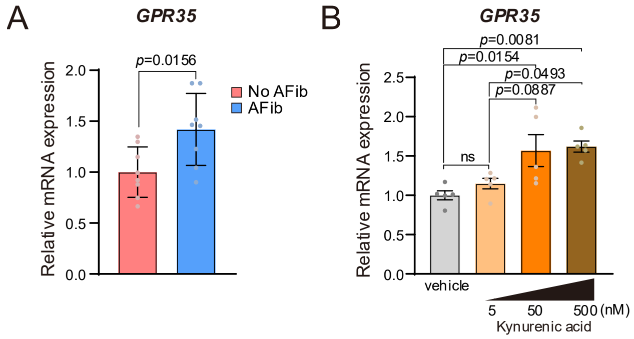

Fig. R3-3. A) Relative mRNA levels of *GPR35* in LECs treated with No AFIb or AFib-EAT conditioned media. $n = 8$ per group, biologically independent samples. Data are mean ± SD.; p value was determined by two-tailed unpaired Student's t-test. **B)** Relative mRNA levels of *GPR35* in LECs treated with vehicle or Kynurenic acid (5 nM, 50 nM, 500 nM). n = 5 per group, biologically independent samples. Data are mean ± SEM.; p value was determined by one-way ANOVA followed by the Tukey-Kramer's *post hoc* test.

While these concentrations appear lower than the reported EC50 values for GPR35 activation, our findings suggest that prolonged exposure to kynurenic acid concentrations, as would occur in the clinical setting, may have cumulative effects on GPR35 signaling through both direct receptor activation and enhanced receptor expression. This dual mechanism could explain why our GPR35 antagonist studies demonstrated significant effects on LECs. These mechanistic details represent important areas for future investigation. Accordingly, we have discussed this issue both in Discussion and limitation section (see Page 25, 26).

Comment #5: *The fact that Ido1 in increased in the EAT and Kat1 and Kat2 are reduced, would suggest a shift to high kynurenine and 3-hydroxykynurenine, possibly even quinolinic acid and not kynurenic acid. What are the levels of those metabolites in the EAT and in the SVF-derived adipocyte cultures? If Kynurenine is up (and that is a much more accepted cytotoxic metabolite than kynurenic acid), then kynurenic acid will be also be elevated due to higher substrate availability, but then the most common consequence is toxicity from high kynurenine. In addition, high kynurenine will be converted into 3-hydroxykynurenine (also a known cytotoxic compound), unless there is a deficit in the enzyme that mediates that conversion (KMO). That should e investigated and shown.*

Response: This is an excellent mechanistic question that addresses the complexity of the kynurenic acid pathway. We appreciate the reviewer's insight regarding the expected metabolic consequences. To address this important concern, we performed additional analyses to comprehensively evaluate kynurenine pathway metabolites in our samples. Our results revealed that while kynurenic acid levels were significantly elevated in both AFib EAT tissue and AFib EAT-conditioned media compared to No AFib controls (**Fig. R3-2, for Comment #3,** previous page), other key metabolites in the pathway including kynurenine, tryptophan, 3-hydroxykynurenine (3-HK), and quinolinic acid showed no significant differences between No AFib and AFib groups in both EAT and EAT conditioned media (**Fig. R3-4A, B**, below).

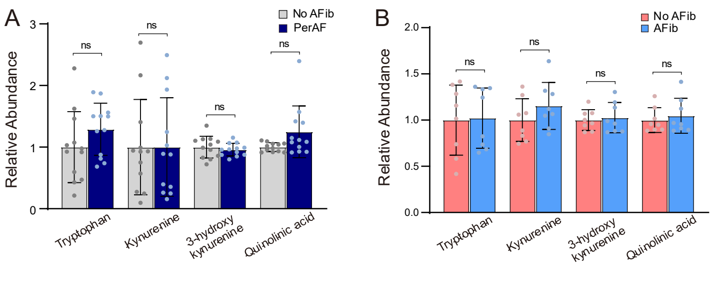

Fig. R3-4. A) Relative abundance of Kynurenine pathway metabolites in EAT from No AFib or PerAF patients. *n* = 12 per group, biologically independent samples. **B)** Relative abundance of Kynurenine pathway metabolites in No AFib or AFib-EAT conditioned media. *n* = 8 per group, biologically independent samples. Data are mean ± SD.; *p* value was determined by two-tailed unpaired Student's *t*-test.

These findings support the specific importance of kynurenic acid in AFib-associated EAT pathophysiology and suggest that the elevation of this metabolite is the key driver of the observed lymphatic endothelial dysfunction. Considering the enzyme mRNA expression results in mouse visceral adipose tissue, the underlying mechanisms of this kynurenic acid accumulation may differ between tissue types and species, and warrant further investigation in future studies. Accordingly, we discussed this issue as a limitation (see Page 25). These results are now shown in **Supplementary Figure S10C, D**.

Comment #6: *What happens to the levels of Kat3 and Kat4?*

Response: We are grateful for the reviewer's thoughtful comments and suggestions. To address the reviewer's question, we have performed the following new experiments:

1. **Analysis of *Kat3* and *Kat4* mRNA expression in both adipose depots**: We measured *Kat3* and *Kat4* expression levels in both EpiWAT (visceral adipose tissue) and IngWAT (subcutaneous adipose tissue) following LY3437943 treatment. In EpiWAT, LY3437943 treatment did not significantly alter *Kat3* and *Kat4* mRNA expression levels. Interestingly, in IngWAT, we observed a significant upregulation of both *Kat3* and *Kat4* mRNA expression following LY3437943 treatment, revealing a previously unrecognized depot-specific response pattern (**Fig. R3-5A**, next page).
2. **Validation through kynurenic acid/kynurenine ratio measurement**: To determine the metabolic significance of these transcriptional changes, we performed mass spectrometry-based analysis to measure the kynurenic acid/kynurenine ratio in both adipose depots. LY3437943 treatment significantly decreased the kynurenic acid/kynurenine ratio specifically in EpiWAT compared to vehicle-treated controls. Importantly, despite the upregulation of *Kat3* and *Kat4* in IngWAT, the kynurenic acid/kynurenine ratio in this depot remained unchanged following LY3437943 treatment (**Fig. R3-5B**, next page).

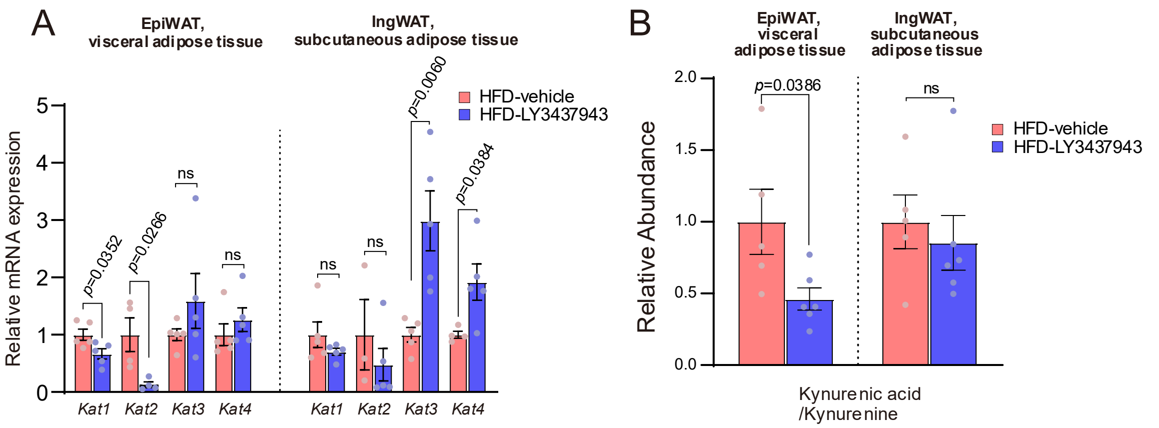

Fig. R3-5. A) Relative mRNA levels of kynurenic acid metabolism related genes in epididymal white adipose tissue (EpiWAT, visceral adipose tissue) and inguinal white adipose tissue (IngWAT, subcutaneous adipose tissue) from obese mice with or without LY3437943 treatment. $n = 3$-5 for both groups, biologically independent mice. Data are mean ± SEM.; p value was determined by two-tailed unpaired Student's t-test. ns, not significant. **B)** Relative abundance of Kynurenic acid/Kynurenine ratio in EpiWAT and IngWAT from obese mice with or without LY3437943 treatment. $n = 5$-6 for each group, biologically independent mice. Data are mean ± SEM.; p value was determined by two-tailed unpaired Student's t-test.

These findings provide important insights into the depot-specific regulation of kynurenine metabolism and confirm that the metabolic changes occur primarily in visceral adipose tissue. The upregulation of *Kat3* and *Kat4* in IngWAT, without corresponding changes in the kynurenic acid/kynurenine ratio, suggests potential compensatory mechanisms or differential post-transcriptional regulation between adipose depots. These results further support our conclusion that visceral adipose tissue represents a preferential therapeutic target for LY3437943-mediated metabolic modulation through the kynurenine pathway. Future studies will be needed to elucidate the molecular mechanisms underlying these depot-specific differences in kynurenine aminotransferase regulation and their potential clinical implications. Accordingly, we discussed this issue as a limitation in this study (see Page 25). These results are now shown in **Main Figure 5N, O, and Supplementary Figure S9C**.

Comment #7: *Another possible endogenous ligand for GPR35 is CXCL17. What are the levels of that chemokine in the EAT and in the SVF-derived adipocyte cultures.*

Response: Thank you for this insightful question regarding CXCL17 as an alternative GPR35 ligand. To address this important consideration, we performed ELISA analysis to evaluate CXCL17 levels in our samples. Our results showed no significant differences in CXCL17 concentrations between No AFib and AFib groups in both EAT and EAT conditioned media (**Fig. R3-6A, B**, below). These results are now shown in **Supplementary Figure S10E, F**.

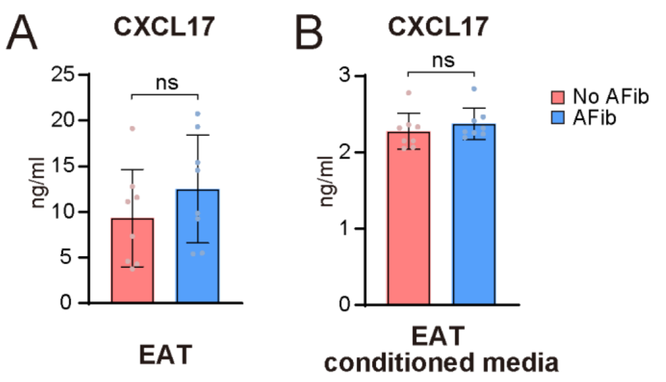

Fig. R3-6. A) Concentration of CXCL17 in EAT from patients with No AFib and AFib. $n = 8$ per group, biologically independent samples. Data are mean ± SD.; p value was determined by two-tailed unpaired Student's t-test. ns, not significant. **B)** Concentration of CXCL17 in No AFib or AFib-EAT conditioned media. $n = 8$ per group, biologically independent samples. Data are mean ± SD.; p value was determined by two-tailed unpaired Student's t-test. ns, not significant.

References cited in the point-by-point letter

Favennec, M., B. Hennart, M. Verbanck, M. Pigeyre, R. Caiazzo, V. Raverdy, H. Verkindt, A. Leloire, G. J. Guillemin, L. Yengo, D. Allorge, P. Froguel, F. Pattou, and O. Poulain-Godefroy. 2016. 'Post-Bariatric Surgery Changes in Quinolinic and Xanthurenic Acid Concentrations Are Associated with Glucose Homeostasis', *PLoS One*, 11: e0158051.

Heijman, J., A. P. Muna, T. Veleva, C. E. Molina, H. Sutanto, M. Tekook, Q. Wang, I. H. Abu-Taha, M. Gorka, S. Künzel, A. El-Armouche, H. Reichenspurner, M. Kamler, V. Nikolaev, U. Ravens, N. Li, S. Nattel, X. H. T. Wehrens, and D. Dobrev. 2020. 'Atrial Myocyte NLRP3/CaMKII Nexus Forms a Substrate for Postoperative Atrial Fibrillation', *Circ Res*, 127: 1036-55.

Yao, C., T. Veleva, L. Scott, Jr., S. Cao, L. Li, G. Chen, P. Jeyabal, X. Pan, K. M. Alsina, I. Dr Abu-Taha, S. Ghezelbash, C. L. Reynolds, Y. H. Shen, S. A. LeMaire, W. Schmitz, F. U. Müller, A. El-Armouche, N. Tony Eissa, C. Beeton, S. Nattel, X. H. T. Wehrens, D. Dobrev, and N. Li. 2018. 'Enhanced Cardiomyocyte NLRP3 Inflammasome Signaling Promotes Atrial Fibrillation', *Circulation*, 138: 2227-42.

Point-by-Point Responses to the Reviewers

Referee #1

Comment #1: *The authors addressed only partially my concerns and some critical issues remain:*
- In response to my concerns that ventricular dysfunction could contribute to the observed arrhythmogenic phenotype, eventually being the primary mechanism of the increased AF susceptibility, the authors only assessed the components of the NLRP3 system in the atria (new Fig. R1-4). However, this does not address my question. The is no functional assessment of the ventricular function in the individual mouse groups (echo, ventricular pressure, etc.), demonstrating absence of overt ventricular dysfunction feedbacking the atria independent of atrial lymphoangiogenesis.

Response: We appreciate the reviewer's important point regarding the potential influence of ventricular dysfunction on atrial phenotype in our mice model. To address this concern, we have performed the following new experiments:

1) **AngII model at early time point (5 days):** To examine the temporal relationship between atrial arrhythmogenic phenotype, lymphangiogenesis, and ventricular dysfunction, we analyzed these parameters at an earlier time point (5 days of AngII infusion). We found that lymphangiogenesis-related gene expression was already significantly decreased and AFib inducibility was significantly increased at this early time point (**Fig. R1-1A-C**, below), while ventricular function assessed by echocardiography showed no detectable changes (**Table. R1-1**, below). These results are now shown in **Supplementary Figure S11A-C, Supplementary Table S6**.

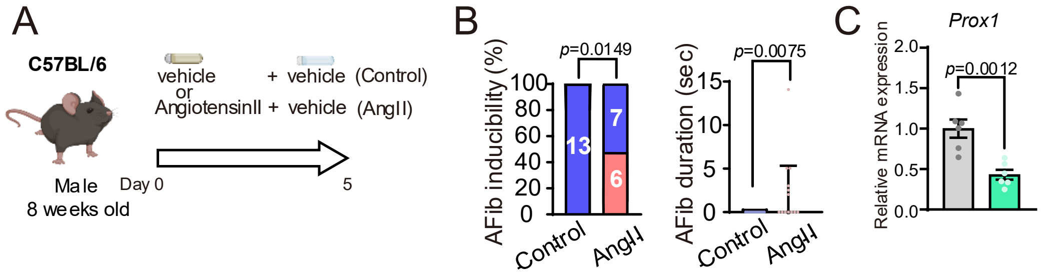

Fig. R1-1. A) Schematic illustration of the experiment procedure in mice. Male C57BL/6J mice at 8 weeks old received vehicle and/or Angiotensin II by osmotic pump for 5 days. **B)** Left: Atrial fibrillation inducibility by transesophageal burst pacing. n = 13 *per* group analysed by Fisher's exact test. Right: Atrial fibrillation duration induced by transesophageal pacing. n = 13 *per* group. Data are median (IQR).; p value was determined by Mann Whitney U test. **C)** Relative mRNA levels of *Prox1* in left atria from vehicle and/or Angiotensin II treated mice for 5 days. n = 6 *per* group, biologically independent mice. Data are mean ± SEM.; *p value* was determined by two-tailed unpaired Student's t-test.

Table R1-1: Echocardiographic analysis for parameters of the cardiac function in mice.

	vehicle	AngII
LVEF (%)	70.0±2.05	70.7±2.01
LVPWT (mm)	0.66±0.02	0.68±0.02
LVDd (mm)	3.51±0.09	3.57±0.11
LVDs (mm)	2.41±0.09	2.40±0.09
E/A	1.43±0.08	1.40±0.08
E/e'	25.3±2.02	25.9±2.03
IVRT (msec)	22.5±0.91	22.9±0.89
IVCT (msec)	18.7±0.75	19.0±0.76
LAD (mm)	2.04±0.06	2.07±0.06

Data are mean ± SEM. n = 13 *per* group. p value was determined by two-tailed unpaired Student's t-test. IVCT; isovolumic contraction time, IVRT; isovolumic relaxation time, LAD; left atrial diameter, LVDd; left ventricular diastolic diameter, LVDs; left ventricular systolic diameter, LVEF; left ventricular ejection fraction, LVPWT; left ventricular posterior wall thickness.

2) **AngII model at 2 weeks with VEGFC intervention:** We examined ventricular function by echocardiography in Control, AngII, and AngII+VEGFC mice at 2 weeks of treatment (**Fig.R1-2A**, below). AngII infusion significantly decreased ventricular diastolic function (**Table. R1-2**, below); however, VEGFC treatment did not affect ventricular diastolic function despite effectively rescuing the atrial AFib phenotype (**Fig.R1-2B, Table. R1-2**, below). This dissociation suggests that the beneficial effects of VEGFC on atrial arrhythmogenesis are mediated through lymphangiogenesis rather than through modulation of ventricular function. These data are now shown in **Supplementary Table S4**.

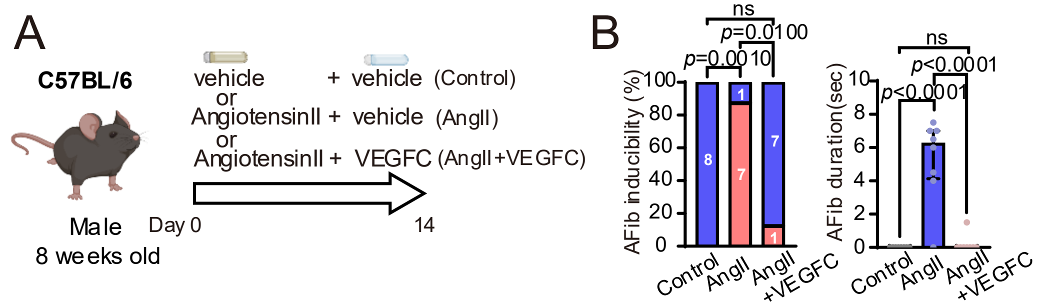

Fig. R1-2 (Original Fig. 3). A) Schematic illustration of the experiment procedure in mice. Male C57BL/6J mice at 8 weeks old received vehicle, Angiotensin II and/or VEGFC by osmotic pump for 14 days (Original Fig.3C). **B)** Left: Atrial fibrillation inducibility by transesophageal burst pacing. $n = 8$ per group, biologically independent mice, analysed by Fisher's exact test. ns, not significant. Right: Atrial fibrillation duration induced by transesophageal pacing. $n = 8$ per group, biologically independent mice. Data are median (IQR).; *p value* was determined by the Kruskal-Wallis test followed by the Dunn's *post hoc* test. ns, not significant (Original Fig.3M).

Table R1-2: Echocardiographic analysis for parameters of the cardiac function in mice.

	vehicle	AngII	AngII + VEGFC
LVEF (%)	69.8±1.94	70.0±1.92	70.3±1.95
LVPWT (mm)	0.66±0.02	0.93±0.04****	0.91±0.03****
LVDd (mm)	3.49±0.09	3.51±0.09	3.49±0.10
LVDs (mm)	2.40±0.08	2.39±0.09	2.40±0.09
E/A	1.49±0.07	1.22±0.07*	1.22±0.08*
E/e'	24.6±1.91	34.7±2.93*	34.6±3.25*
IVRT	22.8±0.82	23.7±1.03	23.4±1.01
IVCT	18.9±0.80	19.1±0.68	19.1±0.80
LAD (mm)	2.01±0.04	2.03±0.05	2.02±0.05

Data are mean ± SEM. $n = 12$ *per* group. * $p < 0.05$, *** $p < 0.001$, **** $p < 0.0001$, *vs* vehicle by one-way ANOVA followed by the Tukey's *post hoc* test. DBP; diastolic blood pressure, HR; heart rate, IVCT; isovolumic contraction time, IVRT; isovolumic relaxation time, LAD; left atrial diameter, LVDd; left ventricular diastolic diameter, LVDs; left ventricular systolic diameter, LVEF; left ventricular ejection fraction, LVPWT; left ventricular posterior wall thickness, SBP; systolic blood pressure, VEGFC; vascular endothelial

3) **HFD obesity model at early time point (6 weeks):** Similarly, we analyzed atrial arrhythmogenic phenotype, lymphangiogenesis-related gene expression, and ventricular function at an earlier time point (6 weeks of high-fat diet). Lymphangiogenesis-related gene expression was already significantly decreased and AFib inducibility was significantly increased, while ventricular function assessed by echocardiography remain unchanged (**Fig. R1-3A-C**, **Table R1-3**, below). These results are now shown in **Supplementary Figure S11D-F, Supplementary Table S7**.

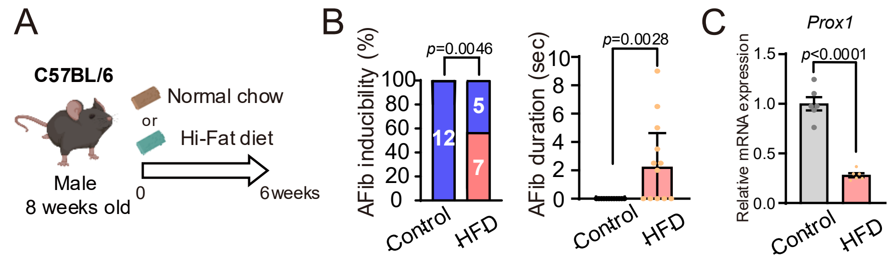

Fig. R1-3. A) Schematic illustration of the experiment for HFD-induced obese mice. Male C57BL/6J mice at 8 weeks old were given access to normal chow or Hi-fat diet (60 % fat) for 6 weeks. **B)** Left: Atrial fibrillation inducibility by transesophageal burst pacing. *n* = 12 *per* group analysed by Fisher's exact test. Right: Atrial fibrillation duration induced by transesophageal pacing. *n* = 12 *per* group. Data are median (IQR).; *p* value was determined by Mann Whitney U test. **C)** Relative mRNA levels of *Prox1* in left atria from mice fed normal chow (NFD) or hi-fat diet (HFD). *n* = 6 *per* group, biologically independent mice. Data are mean ± SEM.; *p value* was determined by two-tailed unpaired Student's *t*-test.

Table R1-3: Echocardiographic analysis for parameters of the cardiac function in mice.

	vehicle	HFD
LVEF (%)	70.4±2.06	70.9±2.15
LVPWT (mm)	0.66±0.02	0.66±0.02
LVDd (mm)	3.52±0.08	3.55±0.08
LVDs (mm)	2.43±0.08	2.45±0.08
E/A	1.49±0.09	1.46±0.10
E/e'	25.8±2.02	25.9±2.36
IVRT (msec)	23.3±0.79	23.1±0.81
IVCT (msec)	18.8±0.87	18.4±0.99
LAD (mm)	2.05±0.06	2.06±0.06

Data are mean ± SEM. *n* = 12 *per* group. *p* value was determined by two-tailed unpaired Student's *t*-test. IVCT; isovolumic contraction time, IVRT; isovolumic relaxation time, LAD; left atrial diameter, LVDd; left ventricular diastolic diameter, LVDs; left ventricular systolic diameter, LVEF; left ventricular ejection fraction, LVPWT; left ventricular posterior wall thickness.

Collectively, these temporal analyses (experiments 1-3) demonstrate that alterations in lymphangiogenesis consistently precede ventricular dysfunction across different time points in our mice model, supporting the concept that impaired lymphangiogenesis is a primary mechanism rather than a downstream consequence of diastolic dysfunction in AngII-, and obesity-induced AFib pathophysiology.

However, we acknowledge that we cannot completely exclude the potential influence of ventricular dysfunction and its hemodynamic consequences on the observed atrial phenotype. Accordingly, we discussed this issue as a limitation in this study (see Page 26).

Comment #2: - *Fig. R1-3: The number of mice per group is very low, partuicularly in the Ang-II group. In D, right panel there is strong trend of increased RyR2 phosphorylation which is not significant because of lack of statistical power. This result does not support the claim of the authors that the lymphoangiogenesis changes precede the development of Ca2+-dependent triggered activity. This part need further experimentation with larger sample sizes to definitely address this issue.*

Response: We thank the reviewer for this important comment regarding the statistical power of our experiments. We acknowledge that the initial sample size in the AngII group was limited, which may have compromised our ability to detect significant differences.
As suggested, we have repeated these experiments with increased sample sizes to address this issue. Despite the increased statistical power, we found that there were no detectable changes in ox-CaMKII and p-RyR2 protein levels compared to control mice at this early time point (1 week) (**Fig. R1-4A, B**). The data are now shown in **Supplementary Figure S6B-D**.

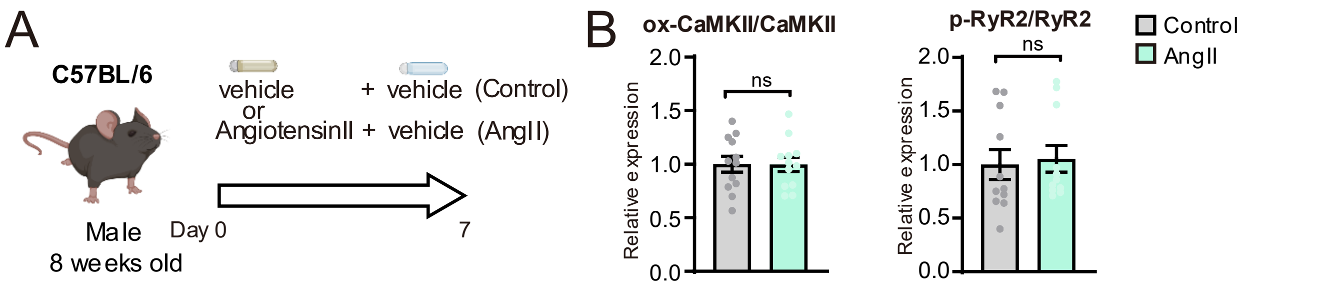

Fig. R1-4. A) Schematic illustration of the experiment procedure in mice. Male C57BL/6 mice at 8 weeks old received vehicle and/or Angiotensin II by osmotic pump for 7 days (Original Fig.S6B). **B)** Quantification of oxidized-CaMKII, CaMKII, p-RyR2 and RyR2 protein. n = 12 per group, biologically independent mice. Data are mean ± SEM.; p value was determined by two-tailed unpaired Student's t-test. ns, not significant.

Referee #2

The authors have correctly addressed and clarified the points addressed by this reviewer. The manuscript has been improved with the additional experiments.

Response: We sincerely thank the reviewer for the careful evaluation of our revised manuscript and for the constructive feedback that helped improve our work. We greatly appreciate the time and effort dedicated to reviewing our study.

Referee #4

In response to reviewer 3's comments, the authors provided satisfactory replies to comments #1, #2, and #7 by conducting additional experiments.

Comment #1-1: *Regarding comment #3, the authors performed mass spectrometry-based analyses to determine the actual concentrations of kynurenic acid (KYNA) in both epicardial adipose tissue (EAT) and EAT-conditioned media from the No AFib and AFib groups. However, the reviewer specifically requested that "actual concentrations should be shown for kynurenic acid throughout the manuscript." The authors did not provide the actual KYNA concentrations in the contexts of AngII-induced or obesity-induced impaired lymphangiogenesis.*

Response: First of all, we are grateful to Referee #4 for continuing the review process and for the careful assessment of our responses to the original comments from Referee #3. We appreciate the positive evaluation of our additional experiments. Regarding the current comment, we thank the reviewer for the opportunity to provide a more comprehensive presentation of kynurenic acid concentrations across all experimental models.

We would like to clarify that our original manuscript examined kynurenic acid-related metabolites only in human EAT samples, EAT-conditioned media, and the HFD-induced obesity model—not in the AngII-induced model, as this model was primarily designed to assess the functional effects of lymphangiogenesis modulation rather than metabolite quantification. However, recognizing the importance of the reviewer's request, we have now performed additional mass spectrometry analyses to determine the actual kynurenic acid concentrations in both mouse models.

The kynurenic acid concentrations in the AngII-induced model were: EpiWAT, median 7.13 (IQR 4.91–15.08) ng/g tissue; IngWAT, median 12.27 (IQR 5.10–26.1) ng/g tissue. In the HFD-induced obesity model, concentrations were: EpiWAT, median 11.04 (IQR 8.58–13.35) ng/g tissue; IngWAT, median 5.35 (IQR 4.01–6.46) ng/g tissue. These concentrations are consistent with those observed in our human EAT samples (**Original Supplementary Figure S10A**).

Notably, further analysis in the AngII-induced model revealed that AngII infusion substantially elevated kynurenic acid concentrations in EpiWAT compared to controls (Control: median 4.64, IQR 4.22–5.89 ng/g tissue; AngII: median 17.55, IQR 12.46–23.58 ng/g tissue), which was partially reversed by VEGFC administration (median 7.13, IQR 5.37–10.26 ng/g tissue). In contrast, in IngWAT, although AngII infusion similarly elevated kynurenic acid concentrations (Control: median 4.68, IQR 3.99–5.46 ng/g tissue; AngII: median 22.14, IQR 13.86–42.53 ng/g tissue), VEGFC administration did not significantly reverse this elevation (median 12.27, IQR 10.00–30.15 ng/g tissue). These findings suggest that the VEGFC-mediated modulation of kynurenic acid may occur preferentially in visceral adipose tissue. We are grateful that the reviewer's suggestion prompted these additional experiments, as these findings provide additional mechanistic support for the involvement of the kynurenic acid pathway in the AngII-induced model as well. These data have been added to our revised manuscript (see Page 26, 27).

Comment #1-2: *The authors demonstrated that 50 nM and 500 nM KYNA upregulate GPR35 mRNA levels in lymphatic endothelial cells (LECs) by approximately 1.5-fold. Nevertheless, they did not report the actual GPR35 expression levels in LECs from the No AFib or AFib groups.*

Response: We thank the reviewer for raising this point regarding GPR35 expression levels in LECs. We would like to respectfully clarify that we had in fact addressed this question in our original submission. As shown in **Fig. R4-1** (original **Supplementary Figure S5**), we demonstrated that *GPR35* mRNA expression was significantly higher in LECs treated with AFib patients-EAT conditioned media compared to those treated with No AFib patients-EAT conditioned media, indicating enhanced sensitivity to kynurenic acid in the AFib setting.

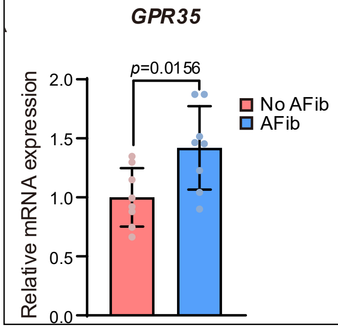

Fig. R4-1. Relative mRNA levels of *GPR35* in LECs treated with No AFib or AFib-EAT conditioned media. n = 8 per group, biologically independent samples. Data are mean ± SD.; p value was determined by two-tailed unpaired Student's t-test.

Nevertheless, recognizing the importance of the reviewer's point regarding direct *in vivo* evidence, we have performed additional immunohistochemical analyses on human left atrial appendage (LAA) tissue from No AFib and AFib patients. Co-immunofluorescence staining for LYVE1 (a lymphatic endothelial marker) and GPR35 revealed that the LYVE1/GPR35 double-positive area was significantly increased in AFib patients compared to No AFib controls (**Fig. R4-2A, B**, below). These results provide direct evidence that GPR35 expression is upregulated in atrial lymphatic endothelial cells in the AFib setting in vivo, consistent with our in vitro findings. These data are now shown in **Supplementary Figure S5B, C**.

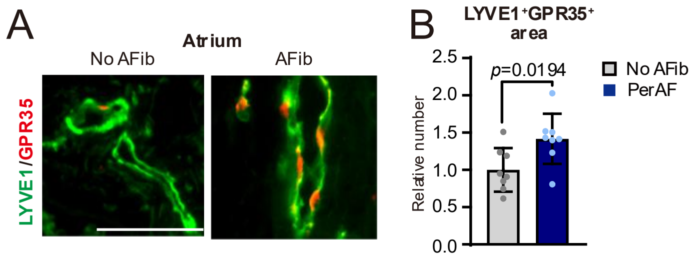

Fig. R4-2. A) Representative immunofluorescent staining for LYVE1 (Green)/GPR35 (Red) in LAA from the patients with No AFib or PerAF. Scale bar, 50 μm. **B)** Quantification of LYVE1$^+$ GPR35$^+$ area in (A). n = 8 for both groups. Data are mean ± SD.; p value was determined by two-tailed unpaired Student's t-test.

Comment #2-1: *In response to comment #5, the authors showed that KYNA levels are elevated in AFib EAT tissue compared to No AFib controls. However, these findings appear inconsistent with the fact that Ido1 is increased in epicardial adipose tissue (EpiWAT), while Kat1 and Kat2 are reduced. The authors did not adequately address this discrepancy.*

Response: We thank the reviewer for this comment and the opportunity to clarify this point. We would like to respectfully highlight an important distinction that may help address this concern: the changes in *Ido1*, *Kat1*, and *Kat2* expression in mouse EpiWAT, as described in **Original Figure 5M-O**, were observed following therapeutic intervention with LY3437943, which is a GLP-1/GIP/glucagon receptor triagonist that induces weight loss. These data represent the effects of a therapeutic weight loss intervention, not the pathological changes associated with obesity or disease progression.

With this context in mind, we believe that these findings may be interpreted as complementary and mutually supportive. The elevated kynurenic acid levels in human AFib EAT (**Supplementary Figure S10A, B**) represent the baseline pathological state in patients with atrial fibrillation. In contrast, following weight loss intervention with LY3437943, despite increased *Ido1* expression, the concurrent suppression of *Kat1* and *Kat2* resulted in a net decrease in the kynurenic acid/kynurenine ratio in visceral adipose tissue (**Fig. 5O**), suggesting that this therapeutic approach may effectively modulate kynurenine pathway metabolism.

Thus, the human data show pathologically elevated kynurenic acid in AFib EAT at baseline, while the mouse intervention data suggest that weight loss therapy may reverse this by modulating kynurenine pathway metabolism. We are grateful to the reviewer for raising this point, as it prompted us to clarify the relationship between these datasets more explicitly. We have discussed this point in the Discussion section of our revised manuscript (see Page 22-23).

Comment #2-2: *Additionally, the relative abundance of kynurenine pathway metabolites was assessed in EAT from No AFib or permanent AF (PerAF) patients, with n=12 per group—a sample size that may be insufficient for robust conclusions.*

Response: We thank the reviewer for this important comment regarding the sample size. We recognize that comprehensive profiling of multiple kynurenine pathway metabolites by mass spectrometry requires substantial tissue volume per sample, which presents a practical constraint on increasing the sample size given the limited availability of human EAT tissue. Nevertheless, to address this concern to the extent possible, we have performed additional experiments and increased the sample size for the analysis of kynurenine pathway metabolites in EAT from No AFib and PerAF patients.

The results with the expanded cohort were consistent with our original findings: kynurenic acid levels remained significantly elevated in AFib EAT compared to No AFib controls, while other key metabolites showed no significant differences between groups (**Fig. R4-3**, below).

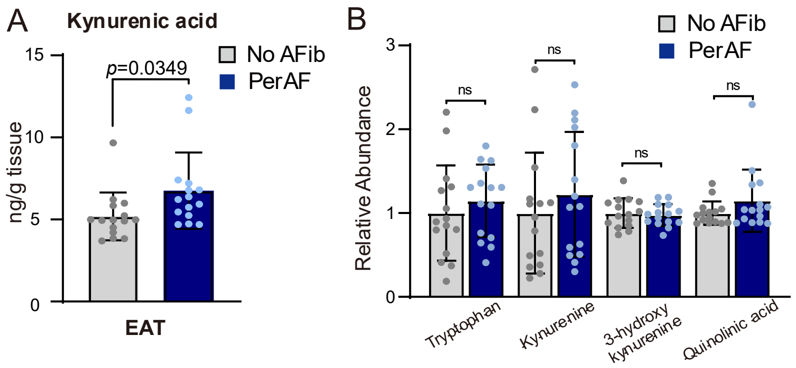

Fig. R4-3. A) Kynurenic acid concentrations in EAT from No AFib or PerAF patients. n = 15 per group, biologically independent samples. Data are mean ± SD.; p value was determined by two-tailed unpaired Student's t-test. **B)** Relative abundance of Kynurenine pathway metabolites in EAT from No AFib or PerAF patients. n = 15 per group, biologically independent samples.

We acknowledge that the sample size remains relatively limited, and we have addressed this issue as a limitation in our revised manuscript (see Page 26).
